# Machine-Learning-Accelerated Design of Ternary Carrier-Free Nanomedicine for Intranasal Therapy of Brain Metastatic Non-small-cell Lung Cancer

**DOI:** 10.34133/research.1180

**Published:** 2026-03-13

**Authors:** Changkun Peng, Gaozheng Li, Xinyue Yin, Annan Xu, Guiting You, Xiangxiang Cai, Mengru Quan, Junjie Zhang, Jie Zhou, Jingying Li, Huanghao Yang

**Affiliations:** ^1^ New Cornerstone Science Laboratory, MOE Key Laboratory for Analytical Science of Food Safety and Biology, College of Chemistry, Fuzhou University, Fuzhou 350108, P. R. China.; ^2^ College of Biological Science and Engineering, Fuzhou University, Fuzhou 350108, P. R. China.; ^3^ Department of Bioengineering and Biopharmaceutics, Fujian Key Laboratory of Natural Medicine Pharmacology, Fujian Key Laboratory of Drug Target Discovery and Structural and Functional Research, The School of Pharmacy, Fujian Medical University, Fuzhou, P. R. China.

## Abstract

Non-small-cell lung cancer (NSCLC) with brain metastases poses formidable therapeutic challenges due to acquired resistance and the inherent pharmacokinetic defects of traditional delivery. We developed an innovative lipoic acid-based self-assembled nanodrug (dabrafenib, trametinib, and lipoic acid self-assembly [DTL]) system, whose rational design was guided by a novel machine learning platform to overcome high-cost, empirical screening bottlenecks. Multifunctional lipoic acid, serving as a universal self-assembling molecule, enabled DTL’s robust assembly and enhanced penetration across mucosal and solid tumor barriers via its unique thiol-mediated exchange mechanism while simultaneously exerting distinct antitumor efficacy. Intranasal administration of DTL achieved efficient dual-targeted delivery to both primary NSCLC and established intracranial metastases. Furthermore, compared to conventional targeted combination therapies, DTL induced diverse, multimodal tumor cell death (apoptosis, pyroptosis, and ferroptosis) and profoundly remodeled the immune microenvironment. In vivo, DTL markedly inhibited tumor growth with reduced toxicity, offering a clinically translatable strategy for advanced NSCLC.

## Introduction

Non-small-cell lung cancer (NSCLC) is a leading cause of global cancer mortality worldwide, with brain metastases occurring in up to 50% of advanced cases, substantially worsening prognosis [1,2]. Current monotargeted therapies, like epidermal growth factor receptor (EGFR) tyrosine kinase inhibitors, face limitations due to acquired resistance, while traditional systemic delivery routes suffer from poor bioavailability and increased systemic toxicity [3,4]. Nanotechnology-based local drug delivery systems offer a promising solution with their targeting and controlled release capabilities [5–8].

Carrier-free nanodrugs (CFNDs) represent a promising paradigm, as they self-assemble solely through intermolecular forces, avoiding exogenous carriers [9]. The term “carrier-free” refers specifically to the exclusion of pharmacologically inert carriers (such as polyethylene glycol, poly(lactic-*co*-glycolic acid), or silica) that typically constitute the bulk of traditional nanomedicines but offer no therapeutic benefit. CFNDs enhance the solubility and stability of poorly soluble drugs while overcoming issues of low drug loading and potential toxicity common in carrier-based systems. CFNDs also enable synergistic multidrug co-delivery, thereby expanding their therapeutic potential [10]. However, CFND development is often hindered by empirical, inefficient screening due to drug molecule-dependent self-assembly, especially for multicomponent systems [11]. Machine learning (ML) offers a powerful solution to these high-cost, low-efficiency bottlenecks. Existing models, however, are primarily limited to 2-component systems and small training datasets, which restrict their application in complex multicomponent CFND design [12–15].

Rational design of combination drug delivery enables clinical patients to achieve synergistic therapeutic benefits beyond additive effects. Lipoic acid (LA), a naturally amphiphilic compound, enhances drug solubility, stability, and self-assembly [16]. Critically, LA’s unique disulfide pentane ring enables highly efficient transmembrane delivery, superior penetration across multiple biological barriers (e.g., mucosal and solid tumor barriers), and specific drug release in high-glutathione (high-GSH) tumor microenvironments via dynamic thiol–disulfide exchange, which is essential for treating both primary and secondary malignant tumor lesions [17–19]. Furthermore, LA exhibits a dual pharmacological activity—acting as an antioxidant in normal tissues and a pro-oxidant in the tumor microenvironment—which aids in immune suppression reversal [20]. LA also inhibits tumor progression and metastasis by affecting various pathways and epithelial–mesenchymal transition (EMT) [21]. Thus, LA offers unprecedented advantages for universal multicomponent nanomedicine strategies.

**Fig. 1.**
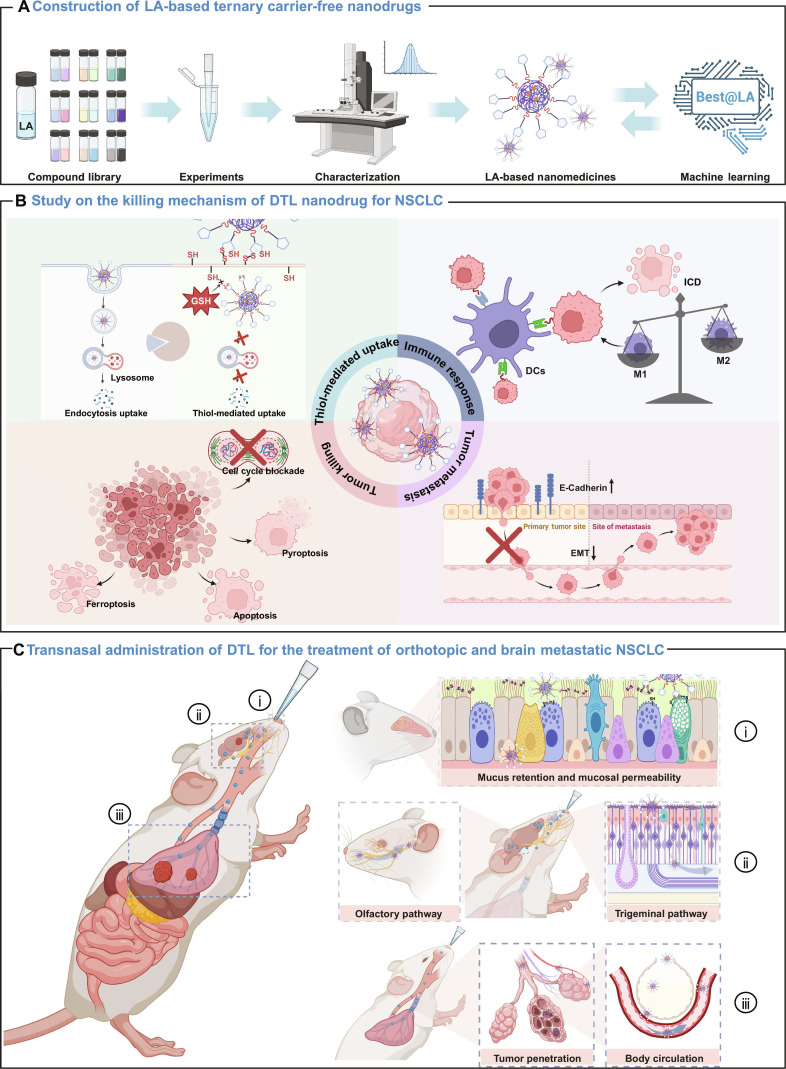
Machine-learning-guided assembly of lipoic acid (LA)-based ternary carrier-free nanodrugs (CFNDs) for the synergistic therapy of primary non-small-cell lung cancer (NSCLC) and established intracranial metastases. (A) Workflow of the integrated “experiment–computation–prediction” strategy that builds a machine learning model for the rational design of LA-based ternary CFNDs. (B) Mechanistic studies elucidate that dabrafenib, trametinib, and lipoic acid self-assembled nanodrug (DTL) exerts multimodal cytotoxicity in epidermal growth factor receptor (EGFR)-mutant PC9 cells, including cell cycle arrest, mitochondrial dysfunction, endoplasmic reticulum stress, and immune activation induced by multiple cell death pathways. (C) Intranasal treatment strategy for orthotopic and brain metastatic EGFR-mutant NSCLC using DTL. (i) DTL undergoes a dynamic thiol–disulfide exchange-mediated transport pathway, effectively penetrating the nasal mucosa. (ii) The nanodrug reaches the brain rapidly through the nasal–brain pathway (e.g., trigeminal and olfactory nerves), improving intracranial bioavailability. (iii) Simultaneously, a substantial fraction is absorbed in the lungs, concentrating at the primary tumor, while a smaller fraction enters the pulmonary vasculature to suppress distal metastases. This scheme was created with BioRender.com and is reproduced under a paid academic subscription. GSH, glutathione; DCs, dendritic cells; ICD, immunogenic cell death; EMT, epithelial–mesenchymal transition.

Based on the challenges of inefficient drug penetration across multiple physiological barriers, the complexities of multicomponent CFND assembly, and LA’s unique properties, our study proposes a tripartite innovative strategy for NSCLC and brain metastasis treatment. Firstly, we constructed a ternary co-assembly database (1,352 combinations) from 63 clinically used small-molecule drugs to train Best@LA—a highly accurate ML model integrating LA as a universal self-assembling module for ternary systems (Fig. 1A). Subsequently, to translate the predictive results into a therapeutic strategy for NSCLC, we selected the clinically approved melanoma drugs dabrafenib (Da) and trametinib (Tra) and co-assembled these agents with LA to form a CFND, dabrafenib, trametinib, and lipoic acid self-assembled nanodrug (DTL), for the treatment of both primary NSCLC and established intracranial metastases (Fig. 1B). Importantly, LA modulates multiple tumor progression pathways, including EGFR, mesenchymal–epithelial transition factor receptor (MET; a common EGFR resistance bypass), mesenchymal–epithelial transition factor receptor (MEK), and EMT, while its pro-oxidant capacity induces mitochondrial/endoplasmic reticulum (ER) stress and inflammatory cascades; Da/Tra targets the MEK signaling axis, effectively blocking EGFR resistance pathways. This multitarget, multimechanism therapeutic network collectively triggers diverse tumor cell death modalities (apoptosis, pyroptosis, ferroptosis, and immune-related cell death) while suppressing metastasis and remodeling the immune microenvironment. This drug-repurposing strategy not only expands the applications of existing clinical agents but also substantially shortens the conventional drug development timeline. Lastly, for NSCLC brain metastasis and low brain delivery efficiency, we employed intranasal administration of DTL (Fig. 1C). This noninvasive strategy enabled efficient DTL accumulation in lung tumors and established intracranial metastases through rapid penetration across mucosal and solid tumor barriers, markedly inhibiting tumor growth while minimizing chemotherapy-related side effects—demonstrating a distinct “reduced toxicity, enhanced efficacy” profile. Our work validates intranasal dual-targeted delivery for thoracic/cranial lesions, presenting a clinically viable strategy.

## Results

### ML-guided design and cellular uptake mechanism of LA-based CFNDs

**Fig. 2.**
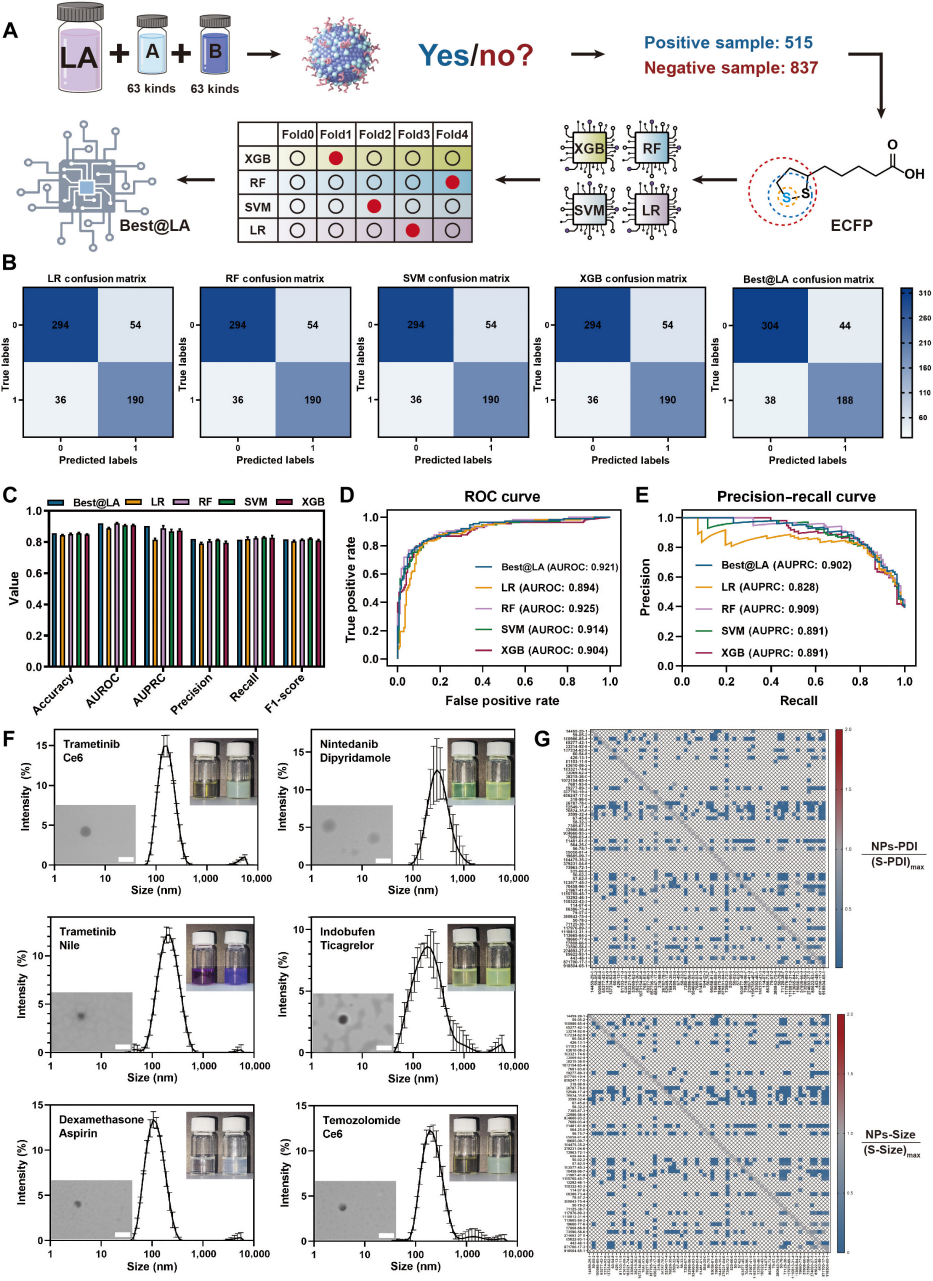
Construction and validation of a machine learning (ML) model for predicting LA-based CFND self-assembly. (A) Schematic diagram illustrating the experimental–computational–predictive workflow for constructing a comprehensive self-assembly database and applying an ML model for the rational design of LA-based CFNDs. (B) Confusion matrix heatmaps comparing the predictive performance of the 5 evaluated ML models on the test set: logistic regression (LR), support vector machine (SVM), random forest (RF), extreme gradient boosting (XGB), and the custom “Best@LA” model. (C) Performance comparison across the 5 models, showing key metrics including F1-score, precision, recall, accuracy, area under the receiver operating characteristic (ROC) curve (AUROC), and area under the precision–recall curve (AUPRC). (D) ROC curves for the 5 ML models, demonstrating their diagnostic accuracy in distinguishing between assemblable and non-assemblable drug combinations. (E) Precision–recall curves for the 5 ML models, further evaluating their classification performance, with a focus on a high proportion of true positives. (F) Dynamic light scattering (DLS) histograms and transmission electron microscopy (TEM) images showing the morphology and particle size distribution of a representative self-assembling nanomedicine. The left sample bottle shows a physical mixture solution of 2 drugs, while the right bottle contains the final, self-assembled 3-drug nanodrug formulation. (G) Heatmap analysis depicting changes in hydrated particle size and polydispersity index (PDI) for all self-assembling CFNDs in the database after the nanoparticle preparation process, highlighting the consistency of the assembly. ECFP, extended-connectivity fingerprint.

To address the bottleneck in screening LA-based co-delivery nanodrugs (CFNDs), we established a systematic “experiment–computation–prediction” intelligent platform (Fig. 2A). First, 63 approved small-molecule drugs were pairwise combined with LA (Table S1). The 2 small-molecule drugs were then assembled at a 1:1 molar feed ratio to ensure their co-encapsulation and synchronous delivery at a fixed equimolar proportion. While maintaining constant amounts of both drugs, the assembly outcomes were systematically evaluated across a gradient concentration range of LA. The results showed that within a combinatorial library of 1,352 LA-containing ternary self-assemblies, 515 formulations could form stable and homogeneous nanoassemblies via nanoprecipitation over the tested LA concentration range, while 837 formulations failed to assemble effectively (Tables S2 and S3). Subsequently, the structural features of each drug pair were digitized using extended-connectivity fingerprints (ECFPs). The hyperparameters of 4 ML algorithms—extreme gradient boosting, random forest, support vector machine, and logistic regression—were systematically optimized via 5-fold cross-validation, and the resulting base learners were integrated through hard voting to construct an ensemble model named Best@LA. On the held-out test set, Best@LA demonstrated superior performance in terms of area under the receiver operating characteristic curve, area under the precision–recall curve, and other key metrics (Fig. 2B to E). To evaluate the practical utility of the model, the remaining 601 uncharacterized combinations were scored, and the 10 highest-ranked formulations were selected for experimental validation. All 10 successfully formed stable nanoassemblies, yielding a positive-predictive value of 100%, which fully confirms the reliability and practicality of the platform for high-throughput screening of LA-based CFNDs (Table S4).

The LA-based nano-delivery system markedly enhanced the aqueous solubility and stability of the small-molecule payloads, both of which are critical for bioavailability (Fig. 2F and G and Table S2). Mechanistic studies on 9 representative LA-based CFNDs revealed that *N*-ethylmaleimide (NEM) pretreatment produced the most pronounced inhibition of cellular uptake (Fig. S1), indicating that thiol-mediated transmembrane delivery is a common internalization pathway. Furthermore, we observed that nanoparticles with a higher proportion of LA in the assembly system exhibited substantially enhanced cellular uptake efficiency (Fig. S2). This finding suggests that the amount of LA, serving as the assembly core, is a key determinant of both system stability and functionality.

### Construction and characterization of DTL nanodrugs

To further explore the potential of LA-based ternary nanodrugs, we targeted the clinical challenge of EGFR-mutated NSCLC. In this context, acquired resistance to EGFR tyrosine kinase inhibitors is driven by a complex network of mechanisms, including secondary EGFR mutations (e.g., T790M and C797S), activation of bypass signaling (e.g., MET/HER2 amplification and BRAF mutation), and the persistent activation of downstream effectors such as PI3K/AKT and RAF–MEK–ERK [3]. Consequently, effective therapeutic strategies must simultaneously intercept these diverse escape pathways. Leveraging our assembly library, we identified DTL (comprising LA, Da, and Tra) as a lead candidate for co-delivery. The design of DTL is grounded in a “multipathway synergistic inhibition” rationale: the clinically approved combination of Da and Tra exerts a dual vertical blockade on the MAPK (RAF–MEK–ERK) pathway, effectively abrogating downstream signaling regardless of upstream mutations (Fig. S3). In parallel, LA functions as a multitarget modulator to inhibit bypass signaling (e.g., MET) and suppress EMT, thereby addressing the resistance mechanisms that escape MAPK inhibition alone [16].

**Fig. 3.**
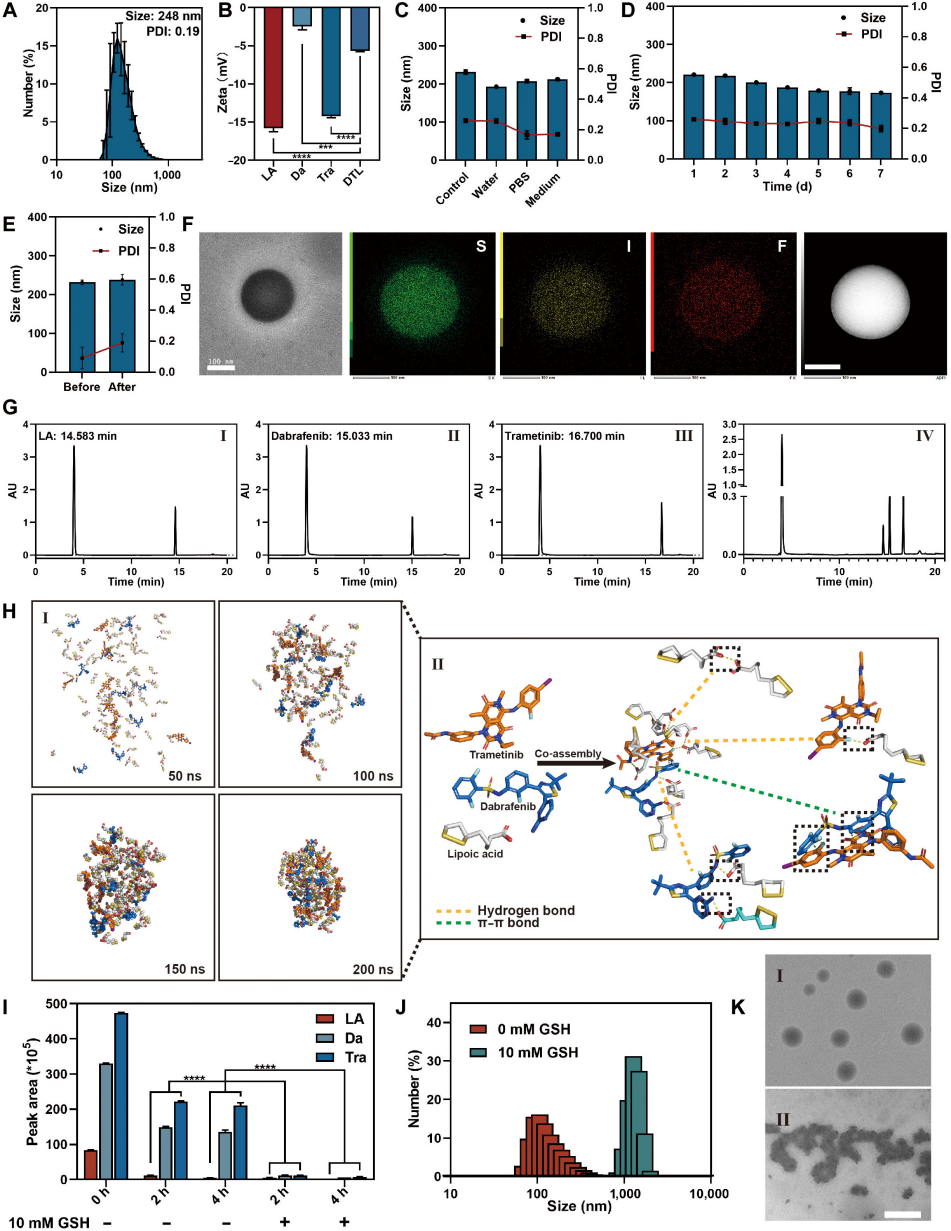
Characterization of DTL nanodrugs and their assembly/release mechanisms. (A) DLS histogram showing the particle size distribution and PDI of purified DTL. (B) Zeta potential measurements of individual small molecules (LA, dabrafenib [Da], and trametinib [Tra]) and the assembled DTL. (C) DLS measurements of DTL’s hydrated particle size and PDI to evaluate its dilution stability in various physiological media. (D) Long-term stability of DTL assessed by DLS monitoring of hydrated particle size and PDI over 7 d. (E) DLS assessment of changes in DTL particle size and PDI before and after concentration. (F) High-resolution TEM image showing DTL morphology (left) and elemental mapping of S, I, and F (right), demonstrating uniform elemental distribution. Scale bar: 100 nm. (G) In the high-performance liquid chromatography (HPLC) analysis, the retention times of the individual components are as follows: (I) corresponds to LA, (II) corresponds to Da, and (III) corresponds to Tra; (IV) shows the HPLC retention times of the various components in the DTL nanoparticles. All analyses were performed under identical chromatographic conditions. (H) Molecular dynamics simulations investigating intermolecular interactions within DTL: (I) Snapshots illustrating the nanoparticle self-assembly process over time (0, 50, 100, 150, and 200 ns). (II) Schematic representation of classical noncovalent interactions (hydrogen bonds and π–π interactions) between LA, Tra, and Da. (I) HPLC analysis quantifying the release of Da and Tra from DTL under conditions with and without GSH, demonstrating GSH-responsive drug release. (J) DLS histogram showing changes in DTL nanoparticle size under GSH-reducing conditions. (K) Transmission electron microscopy (TEM) images illustrating changes in DTL nanoparticle morphology under GSH-reducing conditions. (I) represents the TEM morphology of DTL in aqueous solution; (II) represents the TEM morphology in the presence of 10 mM glutathione (GSH). Scale bar: 200 nm. Data are expressed as mean ± standard deviation (*n* = 3); *t* test; ***P < 0.001; ****P < 0.0001. PBS, phosphate-buffered saline.

Physicochemical characterization revealed that purified DTL nanodrugs possess a uniform spherical morphology with a diameter of ~200 nm, a low polydispersity index (PDI < 0.2), and a negative surface charge (Fig. 3A and B). The nanodrugs exhibited remarkable colloidal stability, preserving their structural integrity against dilution and prolonged incubation in physiological media (Fig. 3C to E). Elemental mapping via transmission electron microscopy (TEM) confirmed the homogeneous co-assembly of Da, Tra, and LA (Fig. 3F), with high-performance liquid chromatography (HPLC) analysis determining an optimized molar ratio of 1:1:22 (Fig. 3G). This specific stoichiometry, established through rigorous screening, reflects a thermodynamic equilibrium that balances high drug loading with assembly stability. To decipher the molecular driving forces, molecular dynamics simulations were employed (Fig. 3H). The assembly is fundamentally driven by the hydrophobic effect, where strong π–π stacking interactions between the aromatic payloads (Da and Tra) generate a compact hydrophobic core. LA acts as an amphiphilic stabilizer: its hydrophobic alkyl chains insert into the core, while its hydrophilic carboxyl groups orient toward the aqueous interface, establishing a hydrogen-bonding network and providing electrostatic repulsion. Most critically, the simulations highlight a unique interfacial architecture where a fraction of the dithiolane rings remains surface exposed. These accessible disulfide moieties do not merely maintain structural cohesion but endow the nanoparticles with dynamic covalent responsiveness, which is essential for thiol–disulfide exchange-mediated mucosal penetration and cellular uptake. Thus, a synergistic network of π–π stacking, hydrophobic interactions, hydrogen bonding, and electrostatic forces orchestrates the self-assembly of DTL into stable and bioactive nanoparticles.

Leveraging the profound physiological GSH gradient, where intracellular concentrations (2 to 10 mM) exceed extracellular levels (~2 to 20 μM) by approximately 1,000-fold, we evaluated the redox-triggered disassembly of DTL. Upon exposure to 10 mM GSH (mimicking the cytosolic environment), DTL displayed a distinct “burst-like” release profile, reaching completion within 2 h (Fig. 3I). Mechanistically, this rapid release is driven by structural disintegration: dynamic light scattering (DLS) monitoring showed a surge in particle size to ~1,000 nm (indicative of aggregation/swelling), and TEM visualization confirmed the rupture of the nanostructure and subsequent payload leakage (Fig. 3J and K). Collectively, these data demonstrate that the intracellular reducing microenvironment effectively cleaves the structural disulfide cross-links of the LA matrix, triggering bulk disassembly and ensuring efficient intracellular drug delivery.

### Endosomal escape mechanisms and mucosal penetration capacities of DTL

**Fig. 4.**
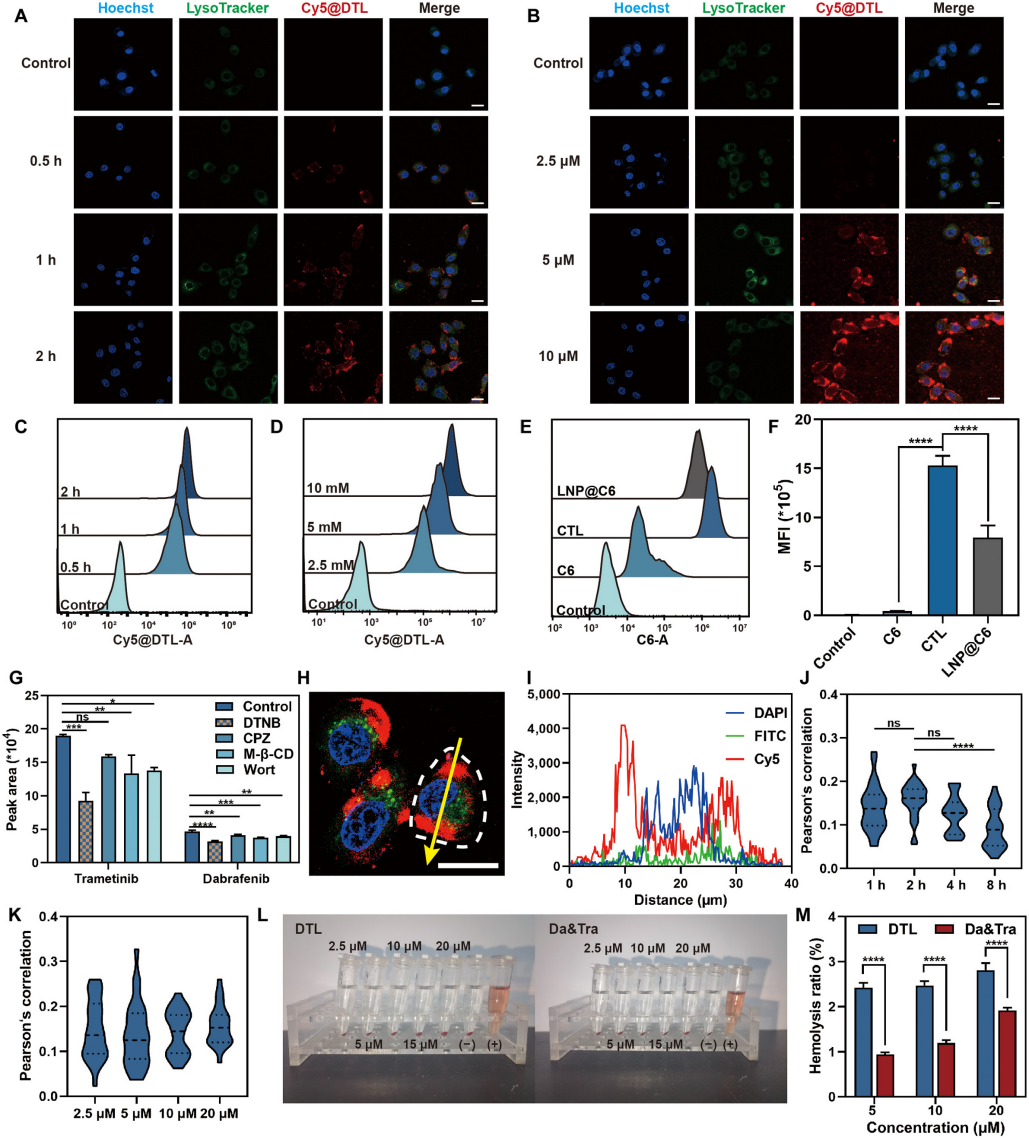
Cellular uptake characterization of DTL in PC9 cells. (A) Confocal laser scanning microscopy (CLSM) images showing the time-dependent cellular uptake of Cy5-Mal-labeled DTL (Cy5@DTL) in PC9 cells. Scale bar: 25 μm (blue: nuclear dye; green: lysosomal dye; red: Cy5@DTL). (B) CLSM images illustrating the concentration-dependent cellular uptake of Cy5@DTL in PC9 cells. Scale bar: 25 μm (blue: nuclear dye; green: lysosomal dye; red: Cy5@DTL). (C) Flow cytometry (FCM) histograms demonstrating the time-dependent cellular uptake of CTL nanodrugs (LA-based nanodrugs with coumarin 6 [C6]) in PC9 cells. (D) FCM histograms showing the concentration-dependent cellular uptake of CTL nanodrugs by PC9 cells. (E) FCM histograms comparing the cellular uptake efficiency of free C6, LA-based nanodrug CTL, and lipid nanoparticles loaded with C6 (LNP@C6) in PC9 cells. (F) Quantitative analysis of the mean fluorescence intensity (MFI) from panel (E), demonstrating the superior uptake of LA-based nanodrugs. (G) Liquid chromatography-tandem mass spectrometry (LC–MS/MS) quantification of endocytosis inhibition efficiency: 5,5′-dithiobis-(2-nitrobenzoic acid) (DTNB), chlorpromazine (CPZ), methyl-β-cyclodextrin (M-β-CD), and wortmannin on DTL uptake. (H) CLSM images showing Cy5@DTL colocalization with endo/lysosomes. Blue: Hoechst 33342 (nuclei); green: LysoTracker Green (lysosomes); red: Cy5@DTL. Scale bar: 15 μm. (I) Fluorescence intensity line profiles across the linear region of interest (ROI) indicated in (B). (J) Time-dependent Pearson’s correlation coefficients for Cy5@DTL and lysosomal/endosomal markers. (K) Concentration-dependent Pearson’s correlation coefficients for Cy5@DTL and lysosomal/endosomal marker. (L) Hemolytic activity assessment: red blood cell suspensions incubated with DTL or Da&Tra at graded concentrations. (M) Quantified hemolysis rates across treatment groups. Data are presented as mean ± standard deviation (*n* = 3); *t* test; ns, no significant difference; *P < 0.05; **P < 0.01; ***P < 0.001; ****P < 0.0001. DAPI, 4′,6-diamidino-2-phenylindole; FITC, fluorescein isothiocyanate.

Cellular uptake assays revealed that Cy5-labeled DTL (Cy5@DTL) follows time- and concentration-dependent kinetics (Fig. 4A to D and Fig. S4). To exclude potential interference from the fluorophore, we cross-validated the uptake efficiency using structurally equivalent LA-based analogs (e.g., CTL). Significantly, these LA-based nanodrugs outperformed both the nondisulfide lipid nanoparticle (LNP) control (LNP@C6) and free coumarin 6 (C6) in PC9 cells (Fig. 4E and F), attributing the superior internalization to the unique surface chemistry of the LA scaffold. Mechanistic profiling further confirmed that while multiple pathways are involved, the uptake of DTL is predominantly driven by thiol-mediated exchange, distinct from classical endocytosis (e.g., clathrin-mediated endocytosis, lipid raft/caveola-mediated endocytosis, and macrophocytosis) (Fig. 4G). Collectively, we propose a spatiotemporally regulated mechanism for DTL transport. In the low-thiol extracellular/mucosal microenvironment, thiol–disulfide exchange is kinetically confined to the nanoparticle interface. This “surface-limited” reaction acts as a molecular anchor for adhesion and transmembrane entry without triggering premature disassembly. Upon cytosolic entry, the steep elevation in GSH concentration functions as a thermodynamic switch, triggering the comprehensive reductive cleavage of the cross-linked network. This results in “burst-like” structural disintegration and rapid cargo release.

Cy5@DTL also displayed remarkable endo/lysosomal escape capability. The escape efficiency increased over time and was independent of concentration (Fig. 4H to K). A hemolysis assay further confirmed that its unique dithiolane ring structure can effectively disrupt membrane integrity to facilitate escape. Notably, DTL showed minimal in vivo hemolysis risk (<5% hemolysis at 20 μM), indicating favorable biosafety and bioavailability (Fig. 4L and M).

Ellman’s assay revealed that approximately 7.8% of the LA molecules on the DTL surface exist in the reduced open-ring form, exposing free sulfhydryl (–SH) groups (Fig. S5a). We attribute this phenomenon to the inherent ring strain of the 1,2-dithiolane moiety in LA. The rapid solvent exchange and steric constraints during nanoprecipitation likely induce a partial ring opening, establishing a dynamic equilibrium where a fraction of LA exists as dihydrolipoic acid. Critically, this surface reduction provides the necessary chemical basis for initiating exchange interactions with mucins. In simulated mucus-coated Transwell assays, DTL exhibited a distinct “stick-and-penetrate” kinetic profile: enhanced penetration was observed in the early phase (<4 h) compared to free drug, followed by substantial mucosal retention at later time points (>8 h) (Fig. S5b and c). This biphasic behavior suggests that thiol–disulfide exchange facilitates mucoadhesion, thereby extending residence time to prevent rapid clearance. At the cellular level, flow cytometry (FCM) confirmed that DTL effectively overcomes the mucus barrier (Fig. S5d and e), implying that the particles utilize dynamic covalent exchange to navigate through the mucin network rather than being immobilized. Finally, using rat intestinal mucosa as a surrogate model rich in mucins containing abundant cysteine residues, we observed that the tissue permeation of DTL was markedly arrested by the thiol-blocking agents 5,5′-dithiobis-(2-nitrobenzoic acid) (DTNB) or NEM (Fig. S5f). This definitively confirms that the mucosal penetration of DTL is strictly dependent on tissue thiol availability and the active exchange mechanism.

### Cytotoxic efficacy and GSH-responsive mechanism of DTL

**Fig. 5.**
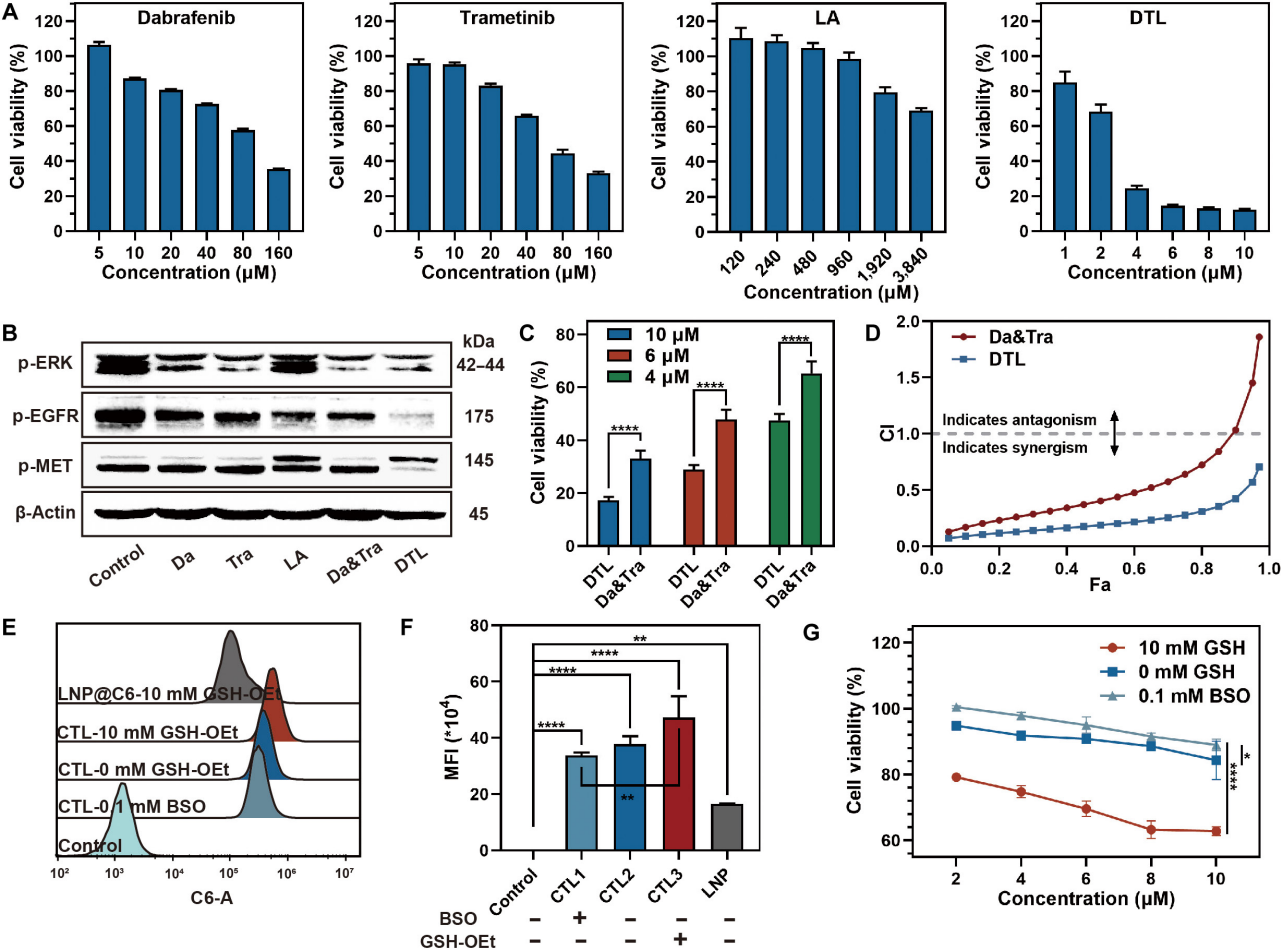
Evaluation of DTL nanodrugs’ cytotoxic activity against PC9 cell line and GSH-responsive mechanism. (A) Counting Kit-8 (CCK-8) assay assessing the cytotoxic activity of individual small molecules (Da, Tra, and LA) and DTL against PC9 cells. (B) Western blot (WB) analysis evaluating the impact of different treatment groups on key cancer-related signaling pathways (p-ERK, p-EGFR, and p-MET). (C) CCK-8 assay comparing the cytotoxic activity of DTL versus the Da&Tra dual-drug combination group on PC9 cells. (D) Combination index (CI) fraction of effect (Fa) curve generated using the Calcusyn software, according to the Chou–Talalay equation, demonstrating synergistic effects. (E) FCM analysis showing the uptake of CTL by PC9 cells pretreated with glutathione ethyl ester (GSH-OEt; GSH supplement) or buthionine sulfoximine (BSO; GSH inhibitor). (F) Statistical quantification of the fluorescence signals from panel (E). (G) CCK-8 assay determining the cytotoxic effects of DTL on PC9 cells with different GSH contents after 24 h. Data are expressed as mean ± standard deviation (*n* = 3); *t* test; *P < 0.05; **P < 0.01; ****P < 0.0001.

We first evaluated the individual cytotoxicities of the components against PC9 cells. Cell Counting Kit-8 (CCK-8) assay confirmed that both Da and Tra exhibited dose-dependent cytotoxicity against EGFR-mutated PC9 cells, while LA demonstrated activity at high concentrations (Fig. 5A). These results support the rationale for the DTL combination. We postulate that the DTL system exerts a comprehensive inhibitory effect by simultaneously targeting the intrinsic EGFR axis (via LA), blocking the downstream effector (via Da&Tra), and suppressing bypass pathways (via LA’s multitarget modulation), thereby preventing tumor escape via common resistance routes.

Western blot (WB) analysis provided molecular validation of this hypothesis, showing that DTL treatment substantially down-regulated the phosphorylation levels of MET, EGFR, and ERK (Fig. 5B and Fig. S6). Subsequently, we assessed the synergistic potential of the system. DTL demonstrated markedly enhanced cytotoxicity compared to monotherapies or the binary Da&Tra combination, indicating robust synergistic inhibitory effects (Fig. 5C and D).

Furthermore, we investigated the impact of intracellular GSH levels on therapeutic efficacy. A high-GSH environment markedly potentiated the cytotoxicity of DTL (Fig. 5E to G). Aligning with the morphological collapse observed in TEM, we attribute this enhancement to the rapid reductive cleavage of the surface disulfide network under high GSH concentrations, which triggers accelerated intracellular drug release. In summary, the potent antitumor efficacy of DTL stems from the multitarget synergy of its payloads, further amplified by its GSH-responsive release mechanism.

### Proteomic analysis decodes DTL’s synergistic mechanisms against NSCLC

**Fig. 6.**
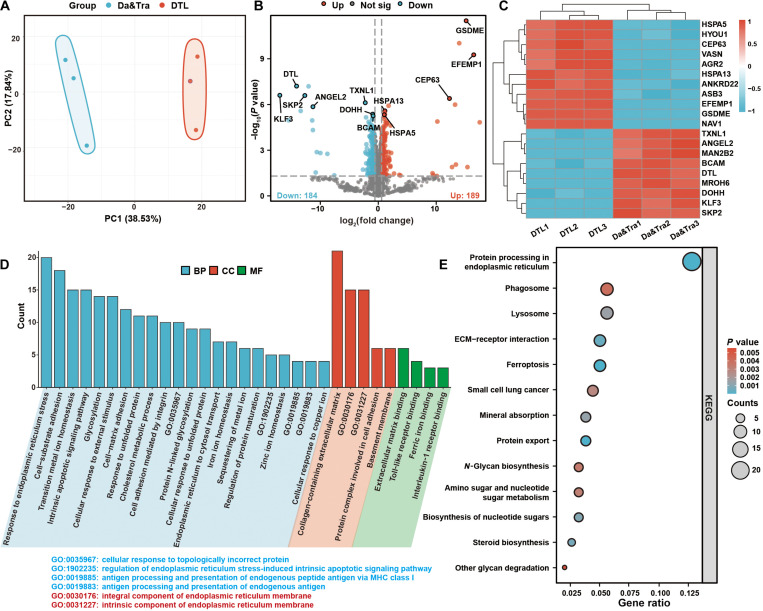
Proteomic analysis of differentially expressed proteins between DTL and Da&Tra treatment groups. (A) Principal component analysis (PCA) plot demonstrating intragroup reproducibility and intergroup differences among proteomic samples from DTL- and Da&Tra-treated cells. (B) Volcano plot illustrating differentially expressed proteins between the DTL and Da&Tra treatment groups. (C) Heatmap displaying the top 20 differentially expressed proteins between the DTL and Da&Tra treatment groups. (D) Gene Ontology (GO) enrichment analysis (Biological Process [BP], Cellular Component [CC], and Molecular Function [MF]) of differentially expressed proteins in DTL- and Da&Tra-treated cells. (E) Kyoto Encyclopedia of Genes and Genomes (KEGG) pathway enrichment analysis of differentially expressed proteins in DTL- and Da&Tra-treated cells.

To elucidate the synergistic mechanisms of DTL, we performed a comprehensive proteomic analysis of the treated PC9 cells. Proteomics revealed 373 differentially expressed proteins between DTL and dual-drug groups (screening criteria: |FC| > 1.5, *P* value < 0.05), with principal component analysis confirming distinct proteomic profiles (Fig. 6A). Up-regulated proteins (e.g., HSPA5, HSPA13, and GSDME) suggested enhanced ER stress, immunogenic cell death (ICD), pyroptosis, and ferroptosis pathways’ activation (Fig. 6B). Down-regulated proteins (e.g., DEPs, TXNL1, KLF3, and BCAM) indicated increased oxidative damage, reactive oxygen species (ROS) accumulation, mitochondrial damage, activation of apoptosis and inflammation pathways, and transfer inhibition (Fig. 6C). DTL likely induces substantial cell cycle arrest, apoptosis via mitochondrial/p53 pathways, strong oxidative stress via TXNL1, enhanced pyroptosis (both classical and nonclassical), and triggers ICD via ER stress. Gene Ontology (GO) and Kyoto Encyclopedia of Genes and Genomes (KEGG) analyses confirmed multitarget antitumor effects, enriching pathways related to ER function, cell death (ferroptosis), metabolism, glycosylation, and microenvironment regulation (Fig. 6D and E).

### Multimodal killing mechanisms of DTL nanodrugs in tumor cells

**Fig. 7.**
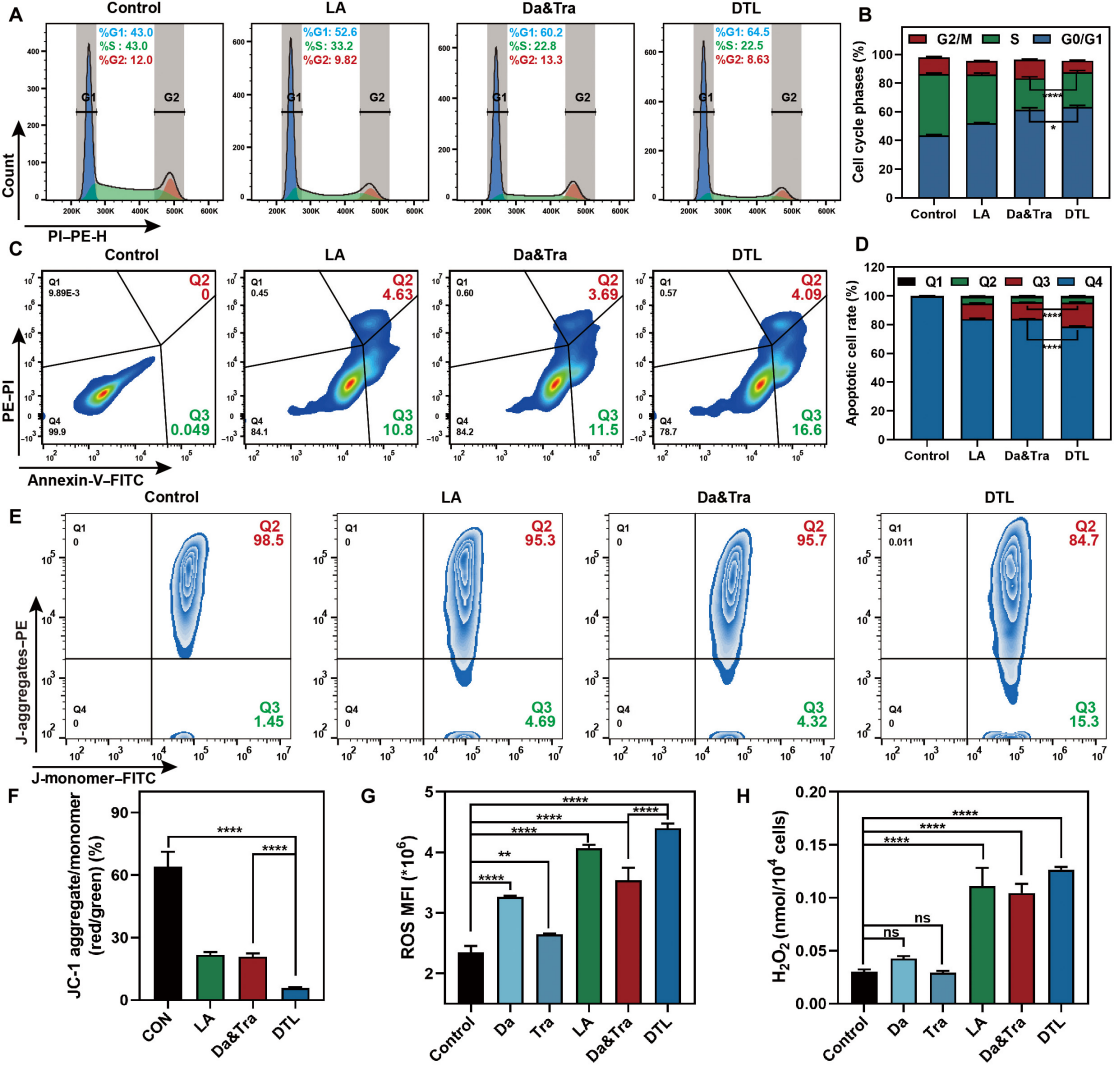
Investigation of DTL killing mechanisms in PC9 cells. (A) FCM analysis of PC9 cell cycle distribution following treatment with different drug groups. (B) Statistical quantification of cell cycle phases from panel (A). (C) FCM plots evaluating the apoptotic induction capacity of different treatment groups on PC9 cells. (D) Statistical quantification of apoptotic rates from panel (C). (E) FCM plots evaluating changes in ΔΨm within PC9 cells after treatment with different drug groups, using the JC-1 probe. (F) Statistical quantification of JC-1 aggregate/monomer ratio from panel (E). (G) Measurement of intracellular reactive oxygen species (ROS) content in different treatment groups. (H) Measurement of intracellular H_2_O_2_ content in different treatment groups. Data are expressed as mean ± standard deviation (*n* = 3); *t* test; ns, no significant difference; *P < 0.05; **P < 0.01; ****P < 0.0001. PI, propidium iodide; PE, phycoerythrin.

To validate the omics-suggested cell cycle arrest, activation of the apoptosis pathway, and mitochondrial damage, we conducted follow-up experiments. FCM showed that DTL markedly inhibited PC9 cell G2-phase DNA replication and induced G1-phase arrest, outperforming dual-drug combinations (Fig. 7A and B). DTL also induced substantially higher early apoptosis (16.6%) and late apoptosis/necrosis (4.09%) compared to the dual-drug group (Fig. 7C and D). LA alone showed comparable apoptosis induction to DTL. DTL markedly reduced mitochondrial membrane potential (20.7% vs. 10% for dual-drug), indicating synergistic effects (Fig. 7E and F). DTL also substantially increased intracellular ROS and H_2_O_2_ levels (Fig. 7G and H), stemming from LA’s pro-oxidative properties that disrupt redox balance and induce mitochondrial damage.

### Pyroptosis induction, immune activation, and invasion inhibition by DTL

**Fig. 8.**
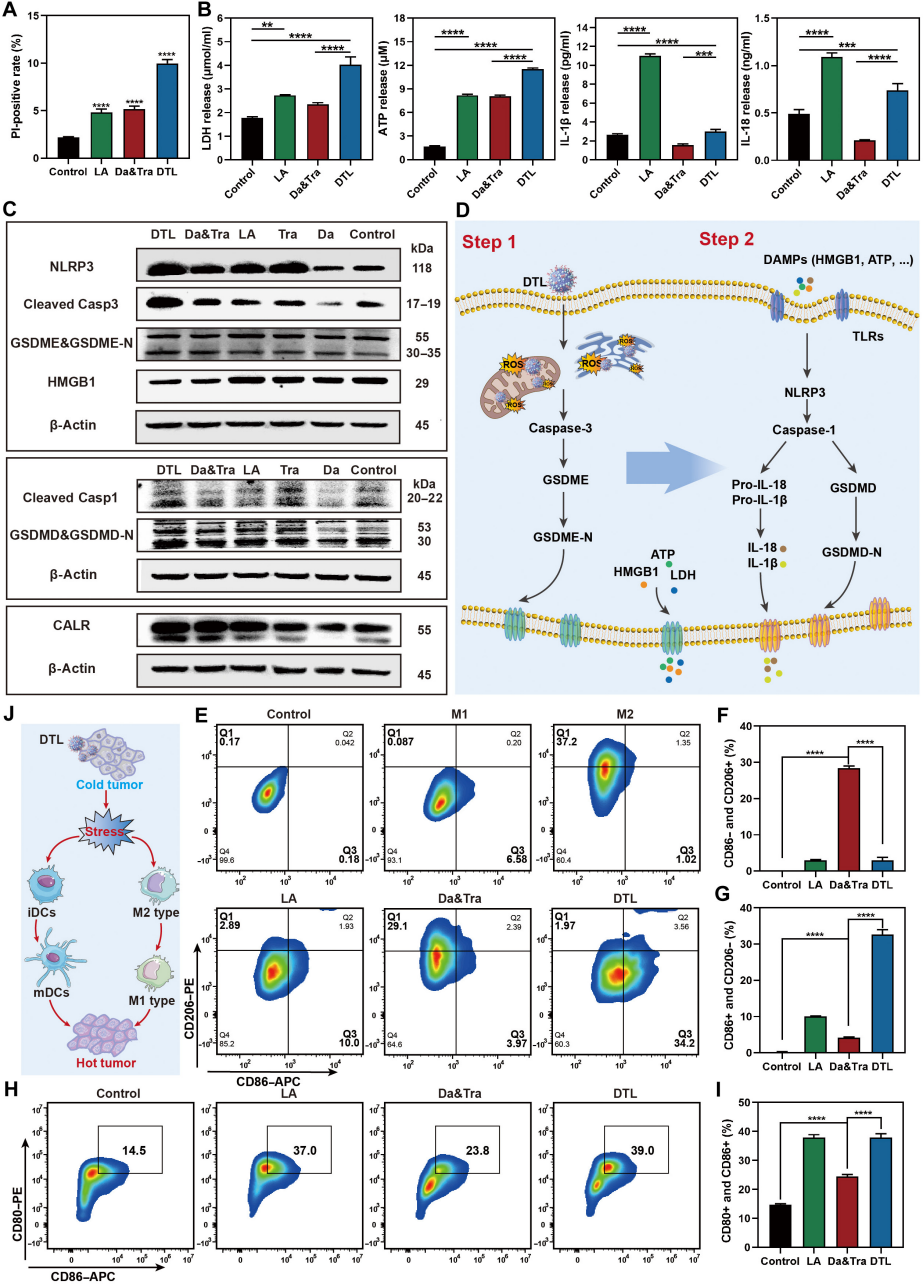
Investigation of DTL-induced pyroptosis in PC9 cells. (A) FCM analysis and statistical quantification of cell membrane rupture degree and PI dye fluorescence signals in different treatment groups. (B) Measurement of lactate dehydrogenase (LDH), adenosine triphosphate (ATP), interleukin-1β (IL-1β), and interleukin-18 (IL-18) levels released by PC9 cells in different treatment groups. (C) WB analysis of key pyroptosis and apoptosis pathway protein expression levels (NLRP3, cleaved Casp3, GSDME&GSDME-N, HMGB1, cleaved Casp1, GSDMD&GSDMD-N, and CALR) in PC9 cells after different treatments. (D) Schematic diagram illustrating the proposed mechanisms of DTL-induced pyroptosis in PC9 cells, involving both classical and nonclassical pathways. (E) FCM plots evaluating the ability of DTL to promote the conversion of M2-type macrophages into M1-type macrophages. (F) Statistical quantification of M1-type macrophages after different treatments. (G) Statistical quantification of M2-type macrophages after different treatments. (H) FCM plots evaluating the effect of tumor-released contents from different treatment groups on the maturation of bone-marrow-derived immature dendritic cells (iDCs). (I) Statistical quantification of myeloid-derived suppressor cells (mDCs) after different treatments. (J) Schematic diagram illustrating DTL-induced immune activation within the tumor microenvironment, including macrophage polarization and dendritic cell maturation. Data are expressed as mean ± standard deviation (*n* = 3); *t* test; **P < 0.01; ***P < 0.001; ****P < 0.0001. DAMPs, damage-associated molecular patterns; TLRs, Toll-like receptors; APC, allophycocyanin.

We then examined the pyroptosis pathway, immune activation, and metastasis inhibition suggested by the omics data. The DTL, LA, and dual-drug groups induced tumor cell membrane damage, with LA and DTL forming distinct pore structures (Fig. 8A and Fig. S7). DTL caused substantial release of adenosine triphosphate and lactate dehydrogenase. The LA group showed the most pronounced interleukin-1β (IL-1β) and interleukin-18 (IL-18) release, followed by DTL (Fig. 8B). WB analysis confirmed that DTL activated both noncanonical (caspase-3/GSDME) and canonical (NLRP3/caspase-1/GSDMD) pyroptosis pathways (Fig. 8C and D and Fig. S8). DTL markedly promoted M2-type macrophage conversion to M1 type (34.2%), far exceeding the dual-drug group (3.97%) (Fig. 8E to G). LA alone also showed M1 conversion (10.0%). The release from DTL-treated cells demonstrated a stronger capacity for immature dendritic cell (DC) maturation capacity (39% vs. 23.8% for dual-drug), with LA alone showing similar promotion (37%) (Fig. 8H and I). This suggests that LA-induced inflammatory cytokines (IL-1β and IL-18) contribute to DC maturation. DTL up-regulated calreticulin and down-regulated HMGB1, which is consistent with ICD. Overall, DTL induces pro-inflammatory contents, activates ICD, and leads to strong immune activation (Fig. 8J). DTL also markedly inhibited tumor cell migration (scratch and Transwell assays) (Fig. S9a, b, d, and e) and invasion (Transwell invasion assays) (Fig. S9c and f) compared to dual-drug combinations. Together, these data show that DTL nanodrugs confer a pronounced anti-metastatic advantage over traditional dual-drug therapy.

### Intranasal DTL for the treatment of primary NSCLC and established intracranial metastases

**Fig. 9.**
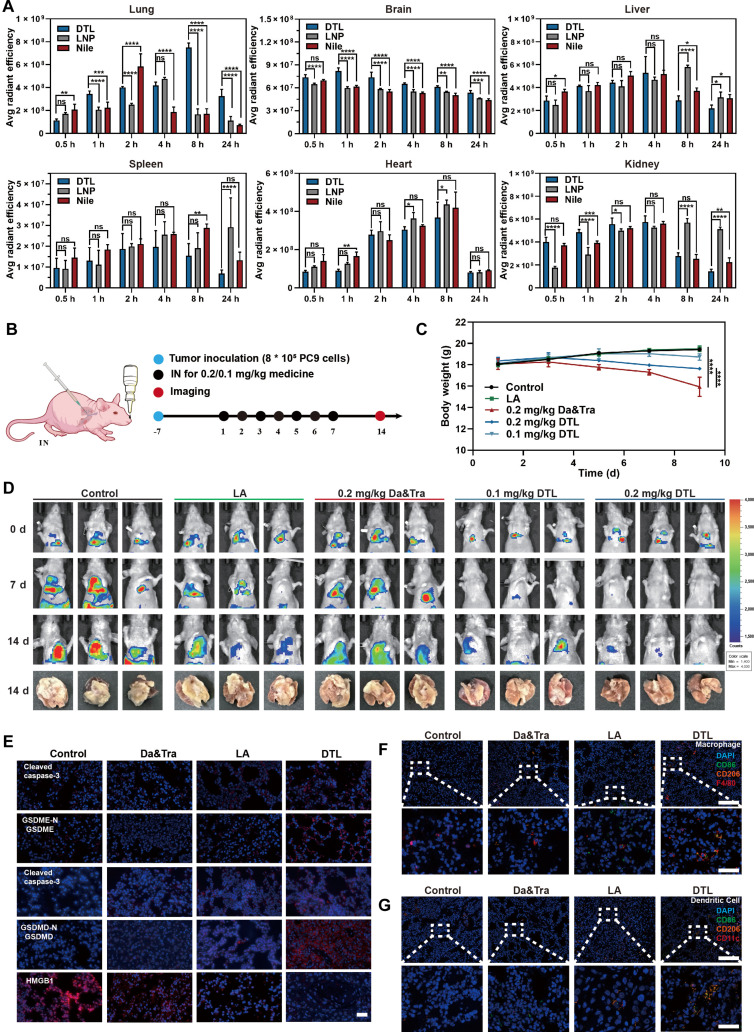
Intranasal administration of DTL for the treatment of in situ NSCLC. (A) In vivo fluorescence imaging showing drug distribution in mouse organs at various time points after intranasal administration. (B) Schematic diagram outlining the treatment process for the in situ NSCLC model. (C) Changes in the body weight of mice during 7 d of continuous treatment. (D) In vivo fluorescence imaging of tumor sites in mice 7 d after treatment and 7 d after discontinuation of treatment. (E) Immunofluorescence images of lung tumor tissue sections showing expression of pyroptosis-related proteins (cleaved caspase-3, cleaved caspase-1, GSDMD, GSDMD-N, HMGB1, and CALR). Scale bar: 100 μm (red: corresponding protein; blue: nuclear stain). (F) Immunofluorescence images of lung tumor tissue sections showing macrophage infiltration (M1/M2 polarization markers). The scale bar in the first row of images is 100 μm, and the scale bar in the second row of images is 25 μm (blue: nuclear stain; green: CD86; orange: CD206; red: F4/80). (G) Immunofluorescence images of lung tumor tissue sections showing DC infiltration. The scale bar in the first row of images is 100 μm, and the scale bar in the second row of images is 25 μm (blue: nuclear stain; green: CD86; orange: CD206; red: CD11c). Data are expressed as mean ± standard deviation (*n* = 3); *t* test; ns, no significant difference; *P < 0.05; **P < 0.01; ***P < 0.001; ****P < 0.0001. IN, intranasal.

The compelling in vitro advantages of DTL nanodrugs prompted a rigorous in vivo evaluation. Intranasal administration successfully achieved dual delivery to both primary pulmonary tumors and established intracranial metastases. Pharmacokinetic profiling revealed that Nile red-labeled DTL exhibited preferential accumulation and extended retention in lung and brain tissues, with minimal off-target distribution. This performance stood in sharp contrast to LNPs, which were predominantly captured by the liver and kidneys via passive clearance (Fig. 9A and Fig. S10). This divergent organ distribution provides strong pharmacokinetic evidence against passive accumulation, reinforcing the conclusion that DTL utilizes active, surface-chemistry-driven targeting mechanisms.

In the orthotopic PC9 lung cancer model, DTL exerted robust antitumor efficacy. Remarkably, low-dose DTL (50%) outperformed the full-dose free drug combination (Fig. 9B to D and Fig. S11), validating the efficiency of the delivery system. Mechanistically, DTL treatment validated the “vertical and horizontal blockade” strategy in vivo by down-regulating MAPK, EGFR, and MET signaling while restoring E-cadherin (Fig. S12). Beyond direct cytotoxicity, DTL induced pyroptosis (Fig. 9E) and reprogrammed the immune microenvironment by recruiting M1 macrophages and mature DCs (Fig. 9F and G). Histopathological analysis showed that DTL markedly reduced the liver and kidney toxicity observed with the dual-drug combination therapy (Fig. S13).

To map the nose-to-brain trajectory, we focused on the initial kinetic window [22]. Quantitative biodistribution analysis showed a rapid surge of Da in the olfactory bulb and brain stem immediately postadministration, establishing a concentration gradient substantially higher than that in the cerebrum (Table S5). This spatial distribution confirms that DTL leverages the olfactory and trigeminal nerve pathways to bypass the blood–brain barrier for direct central nervous system entry.

**Fig. 10.**
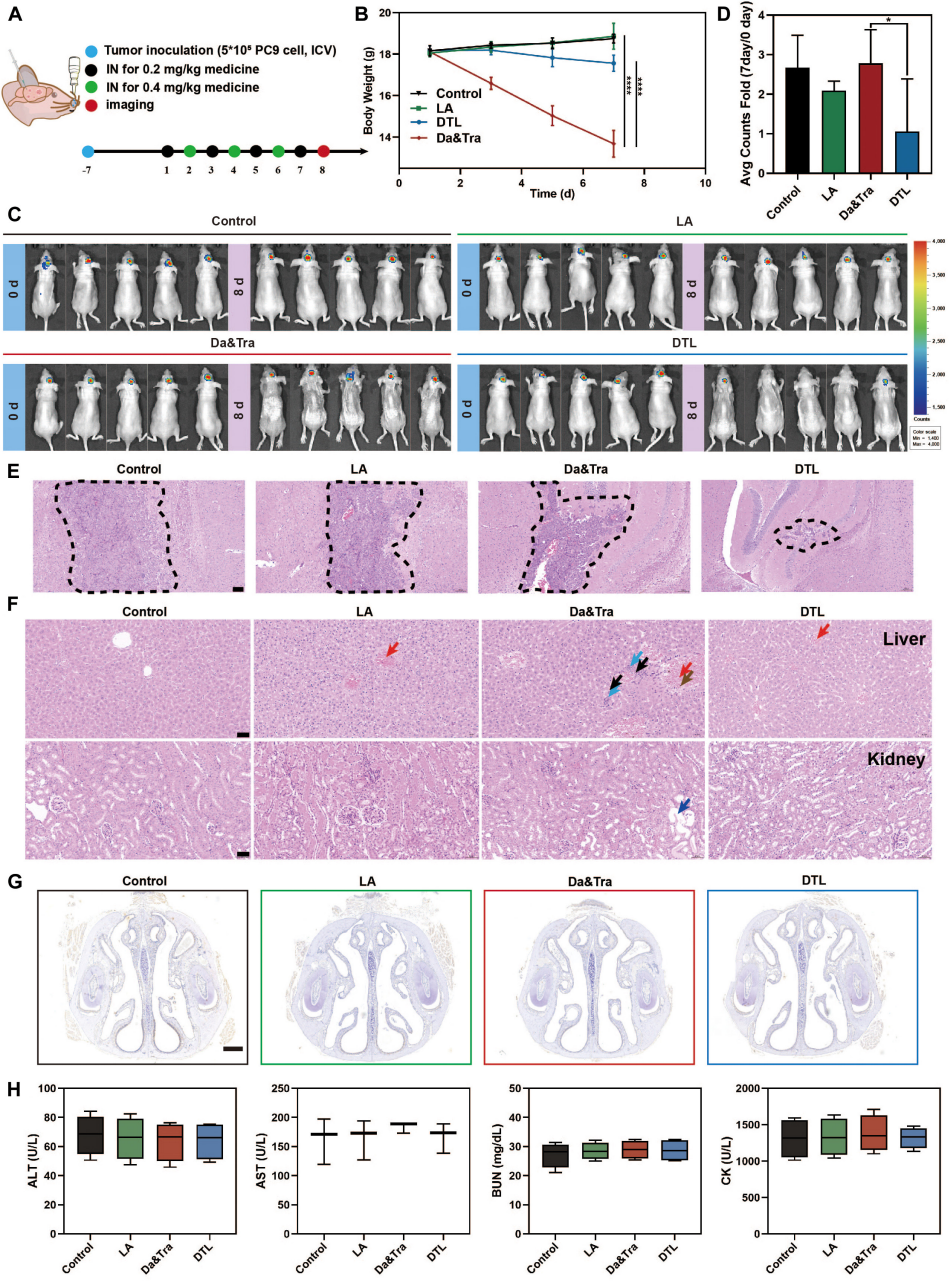
Intranasal administration of DTL for the treatment of brain metastatic NSCLC. (A) Schematic diagram illustrating the treatment process for the brain metastasis model. (B) Changes in mouse body weight during 7 d of continuous treatment. (C and D) In vivo fluorescence imaging of tumor sites and quantitative fluorescence statistics (*n* = 5) after 7 d of treatment, assessing brain tumor growth. (E) Hematoxylin and eosin (H&E) staining of brain tissue sections showing tumor size in different treatment groups. Scale bar: 100 μm. (F) H&E staining showing morphological changes in liver and kidney tissues after intranasal treatment in the PC9 brain tumor model. Scale bar: 50 μm (orange arrows: venous congestion; black arrows: focal hepatic cell necrosis; blue arrows: granulocyte infiltration; dark red arrows: fibrosis; brown arrows: pigment deposition; dark blue arrows: renal tubular dilation). (G) Immunohistochemical analysis of transforming growth factor-β1 (TGF-β1) expression in nasal mucosa, assessing inflammatory response. Scale bar: 500 μm. (H) Measurement of the plasma levels of alanine aminotransferase (ALT), aspartate aminotransferase (AST), blood urea nitrogen (BUN), and creatinine kinase (CK) in different treatment groups, indicating systemic organ function. Data are expressed as mean ± standard deviation (*n* = 3); *t* test; *P < 0.05; ****P < 0.0001. ICV, intracerebroventricular.

In the PC9 brain metastasis model, DTL treatment (alternate-day regimen) led to marked tumor regression (Fig. 10A to E). While the free drug combination caused severe systemic toxicity—manifested as severe weight loss, dermal lesions, and hepatorenal inflammation [23]—the DTL group maintained a superior safety profile. Although mild, transient weight fluctuation was observed in the DTL group (indicative of metabolic stress during intensive therapy), it was substantially less severe than that in the free drug group and devoid of histological organ damage. This highlights a synergistic safety mechanism: precise tumor targeting minimizes systemic exposure, while LA acts as an intrinsic anti-inflammatory and cytoprotective agent to repair off-target stress. Consequently, DTL achieves a high therapeutic index, effectively treating established metastases without inducing the irreversible systemic toxicity common in standard therapies (Fig. 10F to H).

## Discussion

In this study, we engineered an innovative CFND platform based on LA self-assembly, demonstrating its potent efficacy against primary NSCLC and established intracranial metastases via intranasal administration. By integrating a “data-driven” rational design engine with “mechanism-based” therapeutic strategies, we achieved a paradigm shift from empirical screening to predictive precision in nanomedicines.

Our work offers several key advancements.

First, we constructed a high-throughput “experiment–computation–ML” loop, establishing a 1,352-entry database to train the Best@LA model. This platform overcomes the stochastic nature of traditional formulation, offering a standardized road map for multicomponent co-assembly.

Secondly, guided by predictive modeling, the DTL system was assembled via a “synergistic interaction network” involving π–π stacking and hydrophobic effects. Functionally, we identified the unique 1,2-dithiolane ring of LA as the critical hardware for overcoming biological barriers. The surface-exposed disulfide network drives a “stick-and-penetrate” transport mechanism via dynamic thiol–disulfide exchange, ensuring rapid nose-to-brain delivery through the olfactory and trigeminal nerve pathways.

Thirdly, mechanistically, DTL executes a multidimensional attack. It imposes a vertical blockade on the downstream MAPK effector (via Da/Tra) while simultaneously executing a horizontal blockade on upstream EGFR and parallel bypass tracks (via LA), effectively shutting down resistance routes. This is complemented by the activation of ICD (pyroptosis/ferroptosis) and the remodeling of the immune microenvironment.

Fourthly, intranasal DTL achieved dual lung–brain targeting with minimal off-target exposure. This active targeting profile stands in stark contrast to the passive hepatic sequestration of LNPs, validating the superiority of surface-chemistry-driven delivery over size-dependent passive accumulation. Consequently, DTL achieved superior therapeutic outcomes at half the systemic dose, considerably widening the therapeutic window.

Finally, unlike traditional systems where inert excipients constitute the bulk mass, DTL utilizes LA as a “therapeutic building block”. This strategy confers 3 distinct advantages: (a) maximized payload: >90% drug loading efficiency; (b) intrinsic biosafety: elimination of carrier-induced toxicity; and (c) structural precision: thermodynamically driven assembly ensures batch-to-batch reproducibility.

We candidly acknowledge that our current immunocompromised models cannot fully capture the complex tumor–immune dynamics or the spontaneous metastatic cascade. Furthermore, long-term chronic toxicity studies are warranted for clinical translation. Future efforts will focus on expanding the ML-driven database and optimizing LA derivatives to further refine this platform, aiming to provide a robust clinical solution for advanced NSCLC patients facing the challenge of brain metastasis.

## Methods

### Synthesis and characterization of LA-based CFND library

Stock solutions of drug A (10 mM), drug B (10 mM), and α-lipoic acid (LA, 50 mg/ml) in dimethyl sulfoxide were prepared. The ternary mixture was formed by adding 10 μl of drug A, 10 μl of drug B, and 50 μl of LA to a 1.5-ml tube, which was then vortexed thoroughly. To induce nanoprecipitation, 1 ml of ultrapure water was added, followed by immediate vortexing and subsequent standing for 24 h to ensure nanoparticle formation. The size, stability (DLS), and morphology (TEM) of the resulting nanoparticles were characterized. If precipitation occurred within 4 h, the LA ratio was adjusted while maintaining the total organic volume. A systematic pairwise combination of 63 drugs was performed to generate 1,352 distinct LA-based nanoformulations for combinatorial screening.

### Development and validation of the ML prediction model

Assembled nanoparticles were defined as feasible if they exhibited a hydrated particle size of less than 350 nm and a PDI of less than 0.3, as determined by DLS. Molecular features from feasible combinations were extracted using the ECFP algorithm. The dataset was randomly split into training and testing sets to train 4 ML models: logistic regression, support vector machine, extreme gradient boosting, and random forest. Optimal hyperparameters for each algorithm were identified via 5-fold cross-validation, and the resulting base learners were integrated by hard voting to yield the ensemble model Best@LA; predictive capacity was subsequently assessed on the held-out test set. We have made the complete source code, training datasets, and detailed execution instructions publicly available on GitHub (link: https://github.com/changkunpeng/Machine-Learning-Accelerated-Design-of-Ternary-Carrier-Free-Nanomedicine).

### Synthesis, purification, and characterization of DTL

The DTL ternary assembly was performed as described in the library construction method. Unbound drugs were removed by dialyzing the nanoparticles against water for 24 h using a regenerated cellulose dialysis membrane (molecular weight cutoff [MWCO]: 3,500 Da) under constant agitation (150 rpm). The sample was then centrifuged (2,500 rpm, 5 min) to concentrate DTL via the resulting pellet.

The purified DTL was characterized using a suite of techniques. The hydrodynamic size distribution, the zeta potential, and stability were measured by DLS. Morphology was examined by TEM at 100 kV, with samples air-dried on copper grids. Elemental mapping for S, I, and F was performed using energy-dispersive x-ray spectroscopy–ultrahigh-resolution TEM. DTL’s compositional ratios were determined by HPLC using an XB-C18 column with a gradient elution method. Disulfide bond opening was verified by incubating DTL with GSH, followed by adding DTNB and measuring the absorbance at 412 nm.

### GSH-responsive release and molecular simulation

DTL nanoparticles were loaded into regenerated cellulose dialysis bags (MWCO: 1,000 Da) and incubated in phosphate-buffered saline (PBS; pH 7.4) or 10 mM GSH/PBS at 37 °C. Samples collected at 0, 2, and 4 h were analyzed by HPLC to quantify the release kinetics of Da, Tra, and LA. Changes in particle size and morphology under reducing conditions were monitored by DLS and TEM after a 1-h incubation with 10 mM GSH. Molecular dynamics simulations using the Martini model in GROMACS were performed to investigate the co-loading mechanism and intermolecular interactions of the 3 components.

### In vitro mucus retention and penetration assays

The retention capacity of DTL was assessed using a Transwell model. A mixture of nanodrugs and simulated nasal mucus was added to the upper chamber, with PBS in the lower chamber. Samples from the lower chamber were collected at specified time points (1, 2, 4, and 8 h) for quantitative analysis using an IVIS in vivo imaging system or a microplate reader. The penetration ability of DTL through mucus was also evaluated by FCM on PC9 cells incubated with drug/mucus mixtures. Furthermore, in vitro nasal penetration was evaluated using rat intestinal mucosa segments in Franz diffusion chambers, with drug concentrations in the receiver compartments quantified by liquid chromatography–quadrupole time-of-flight mass spectrometry.

### Cellular uptake and endosomal escape

DTL was labeled with Cy5-Mal overnight to generate DTL@Cy5. Uptake efficiency in PC9 cells (5 × 10^4^/well) was assessed in a time- and concentration-dependent manner using FCM and confocal microscopy. The contribution of specific endocytosis pathways was determined by pretreating cells with various inhibitors (DTNB, wortmannin, chlorpromazine, methyl-β-cyclodextrin, and NEM) before nanodrug incubation. Endosomal/lysosomal escape was assessed by confocal microscopy with nuclear/lysosomal staining, quantifying colocalization via Pearson’s coefficient. Hemolysis assays using red blood cell suspensions from C57BL/6 mice were performed to assess in vivo hemolytic risk.

$$\begin{array}{c} \text{Hemolysis rate}\;\left(\text{\%}\right)=\frac{\mathrm{Abs}\;\text{sample}-\mathrm{Abs}\;\text{negative}}{\mathrm{Abs}\;\text{positive}-\mathrm{Abs}\;\text{negative}}\times 100\text{\%} \end{array}$$ (1)

Abs sample denotes the absorbance of treated samples, Abs negative represents the untreated control group, and Abs positive indicates the 1% Triton X-100-lysed group.

### In vitro efficacy and mechanism studies

PC9 cell viability was assessed using the CCK-8 assay after treatment with various monotherapeutic or combination regimens, including GSH-modulated treatments. Cell cycle arrest was analyzed by FCM after cells were fixed and stained with propidium iodide (PI). Apoptosis was evaluated using annexin V–fluorescein isothiocyanate/PI dual staining and FCM. Changes in mitochondrial membrane potential (ΔΨm) were quantified by FCM after staining with the JC-1 probe. The ability of DTL to induce oxidative stress was assessed by measuring intracellular ROS and H_2_O_2_ levels. Membrane integrity was determined by inverted microscopy and FCM using PI staining. The release of inflammatory factors (IL-1β and IL-18) and damage markers (adenosine triphosphate and lactate dehydrogenase) was quantified using their respective kits. Key protein expression levels were analyzed by WB. The ability of DTL to induce M1 macrophage polarization and DC maturation was evaluated by FCM using specific markers. Finally, the anti-metastatic effects of DTL were assessed via scratch migration and Transwell migration/invasion assays.

### Proteomic analysis

Global proteomic profiling of differentially treated PC9 cells (80% to 90% confluent in 100-cm^2^ dishes) was performed following 24-h drug exposure. Quality-control-validated protein extracts underwent mass spectrometry, differential expression analysis, and functional annotation via GO/KEGG enrichment.

### Animal studies and biosafety evaluation

Orthotopic PC9-LUC1 lung cancer models were established in Balb/c-nu mice via intratracheal injection (8 × 10^5^ cells/mouse). Therapeutic intervention commenced on day 7 postinoculation with daily intranasal administration for 7 d. Drug biodistribution was assessed by ex vivo imaging of major organs using Nile red-labeled nanodrugs. Brain cancer models were constructed by stereotactic implantation of PC9 cells into the cranium (1 × 10^8^ cells/ml, 5 μl at 2 μl/min). Treatment commenced on day 7 with an every-other-day doubled-dose intranasal regimen. Both models were used for tumor growth assessment, histopathological analysis, and comprehensive biosafety evaluation via hematoxylin and eosin staining of major organs and measurement of key plasma markers. Immunohistochemistry and immunofluorescence were performed on tissue sections to analyze protein expression and immune cell infiltration.

### Statistical analysis

All data are expressed as mean value with a standard deviation (±SD) or standard error of the mean (±SEM). Statistical analysis is performed using a *t* test, and a *P* value <0.05 is considered significant ns, no significance; *P < 0.05; **P < 0.01; ***P < 0.001; ****P < 0.0001.

## Data Availability

All data generated or analyzed during this study are included in this published article (and its Supplementary Materials files) or are available from the authors upon request.

## Supplementary Materials

Supplementary 1 Experimental procedures Figs. S1 to S13 Tables S1 to S5
