# Supplementary material for: Machine-Learning-Accelerated Design of Ternary Carrier-Free Nanomedicine for Intranasal Therapy of Brain Metastatic Non-small-cell Lung Cancer
Source: Research (Wash D C). 2026 Mar 13;9:1180. doi: 10.34133/research.1180 (PMC12982895; doi:10.34133/research.1180)
Supplement: Supplementary 1 — Experimental procedures Figs. S1 to S13 Tables S1 to S5 [file research.1180.f1.zip › si (Anonymous)-1207.docx]

**Supporting Information**

**Machine Learning-Accelerated Design of Ternary Carrier-Free Nanomedicine for Intranasal Therapy of Brain Metastatic Non-Small Cell Lung Cancer**

Experimental procedures

Supplementary Figures 1-12

Supplementary Tables 1-4.

**1. Experimental Procedures**

**1.1 Materials**

63 drugs used for drug library construction: Shanghai Macklin Biochemical (α-lipoic acid, Aspirin, Paclitaxel, Clopidogrel, Nintedanib, Prasugrel, Cilostazol, Hematoporphyrin); APExBIO Houston USA (Doxorubicin, Acyclovir, Bosutinib, Dabrafenib, Darolutamide, Defactinib, Chlorin e6, Fluorometholone, Gemcitabine, Cobimetinib, Hydroxycamptothecin, Trametinib, Sacituzumab govitecan, Sunitinib, Vemurafenib, Camptothecin); Dalian Meilun Biotechnology (Amoxicillin, Erlotinib, Omeprazole, Dexamethasone, Temozolomide, Domperidone, Doxycycline, Famotidine, Furazolidone, Voriconazole, Fluconazole, Erythromycin, Baicalein, Gefitinib, Methotrexate, Metronidazole, Tetracycline, Clarithromycin, Lansoprazole, Rabeprazole, Rifampicin, Chloramphenicol, Meloxicam, Natamycin, Nile Red, Norfloxacin, Pranoprofen, Ticagrelor, Ketoconazole, Oxytetracycline, Tobramycin, Cimetidine, Indocyanine Green, Levofloxacin); MedChemExpress (Propranolol, Coumarin 6); Shanghai Yuanye Bio-Technology (Dipyridamole, Indobufen); and Shanghai Aladdin Biochemical (Propranolol hydrochloride).

Antibody: Met (D1C2 XP, 1:1000), p-Met (Tyr1234/1235, 1:1000), EGFR (D38B1 XP, 1:1000), p-EGFR (Tyr1068, D7A5 XP, 1:1000), Caspase-1 (D7F10, 1:1000), Cleaved Caspase-1 (Asp297, D57A2, 1:1000), GSDMD (E9S1X, 1:1000), Cleaved GSDMD (Asp275, E7H9G, 1:1000), HMGB1 (D3E5, 1:1000), Calreticulin (D3E6 XP, 1:1000), β-Actin (13E5, 1:2000), p-ERK1/2 (Thr202/Tyr204, D13.14.4E XP, 1:1000), ERK1/2 (137F5, 1:1000) from Cell Signaling Technology; DFNA5/GSDME-N-terminal (1:1000) from abmart; NLRP3 (ab283819 [RM1021], 1:1000) from abcam; F4/80 (1:100), CD86 (1:100), CD206 (1:100), CD80 (1:100) from Proteintech; CD11c (1:100) from CohesionBio.

Assay kit: All experimental kits were sourced from certified manufacturers: CCK-8 Cell Proliferation Assay Kit (Meilun Biotechnology); LysoTracker® Green DND-26 (Beyotime Biotechnology); Annexin V-FITC/PI Apoptosis Detection Kit (BD Biosciences, distributed by Meilun Biotechnology); Cell Cycle & Apoptosis Analysis Kit (PI/RNase staining buffer) and MitoProbe™ JC-1 Assay Kit (both Meilun Biotechnology); DCFH-DA ROS Detection Kit (Beyotime Biotechnology); H₂O₂ Assay Kit (BC3595), LDH Cytotoxicity Assay Kit (BC0685), ATP Bioluminescence Assay Kit (BC0995), and H&E Staining System (G1120) (Solarbio Science & Technology); Human IL-18/IL-1β ELISA MAX™ Deluxe Sets (BioLegend, distributed by MULTISCIENCES BIOTECH); Opal™ 4-plex IHC/IF Kit (Akoya Biosciences, distributed by RecordBio); and ImmPRESS® VR HRP Kit (Vector Laboratories, distributed by Proteintech).

**1.2 Cell cultures**

PC9 cells (Zhejiang Meisen) and PC9-LUC1 cells (Zhejiang Meisen) were cultured in RPMI-1640 (Gibco, 11875093) medium containing 10% FBS (OPCEL-BS-1101) and 1% penicillin/streptomycin (MeilunBio-PWL062). PC9-LUC1 cells were maintained in medium supplemented with 1 μg/mL puromycin. RAW 264.7 macrophages (Shanghai Institute of Life Sciences) and bone marrow-derived dendritic cells (BMDCs) (isolated from C57BL/6 mouse femurs) were cultured in DMEM medium containing 10% FBS and 1% penicillin/streptomycin. BMDCs media was also supplemented with 20 ng/mL GM-CSF (Novoprotein, Shanghai, China; CK02) and 10 ng/mL IL-4 (Novoprotein, Shanghai, China; CK74). All cells were incubated at 37°C under 5% CO2.

**1.3 Animal Models**

BALB/c mice (18–20 g, 6–8 weeks old) and BALB/c-nu athymic nude mice (18 g) were purchased from Beijing Vitonlihua Laboratory Animal Technology Co., Ltd. All animal experiments were conducted in accordance with the standards of the Animal Protection and Use Committee of Fuzhou University.

**2. Supplementary Figures**


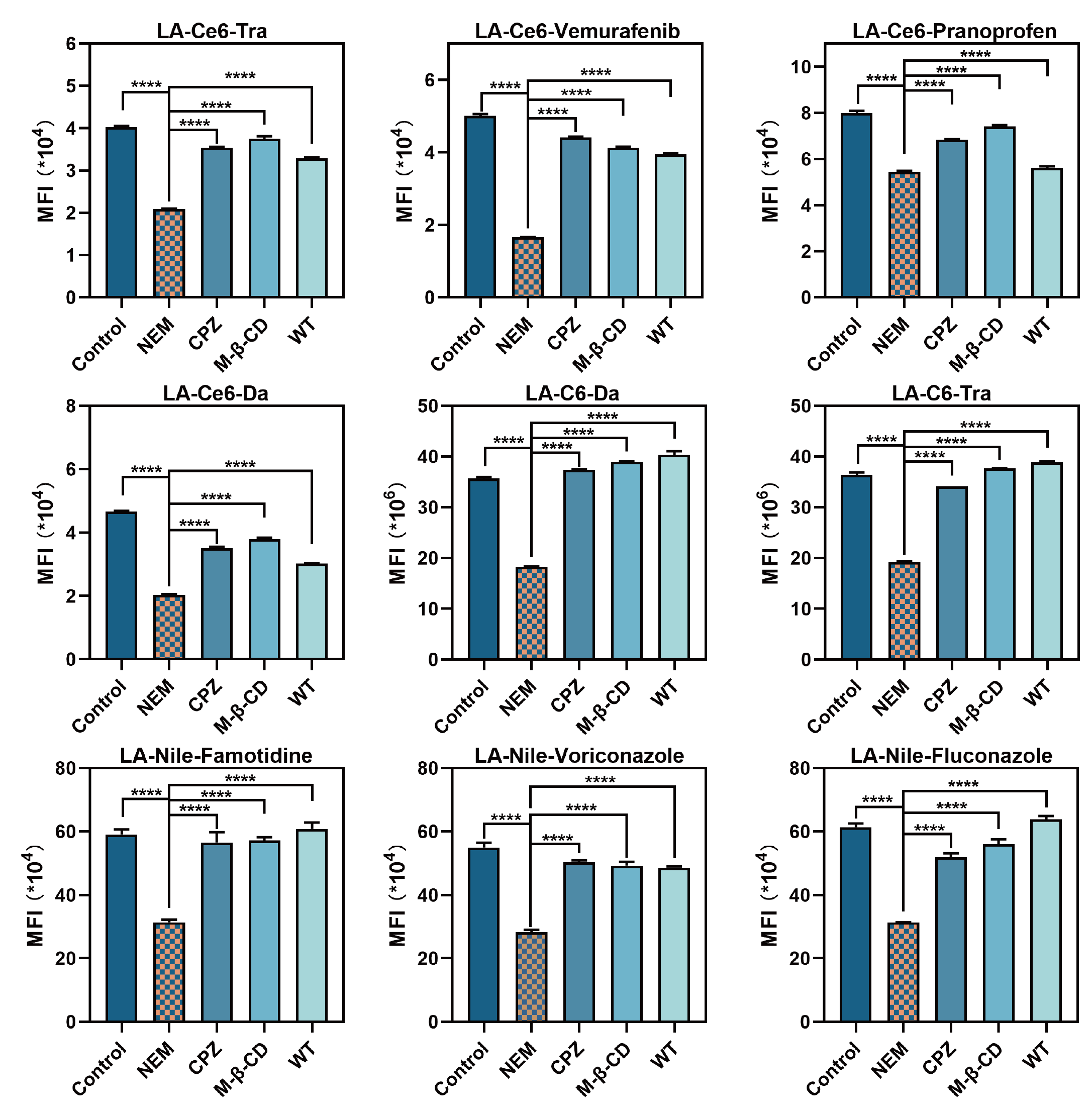


**Figure S1. Cellular uptake mechanisms of nine representative LA-based CFNDs.** Mean Fluorescence Intensity (MFI) of cells treated with various LA-based CFNDs following pre-treatment with different endocytosis inhibitors: N-ethylmaleimide (NEM), chlorpromazine hydrochloride (CPZ), methyl-β-cyclodextrin (M-β-CD), and wortmannin (WT). Data are expressed as mean ± standard deviation (n = 3); t-test; ****p < 0.0001.


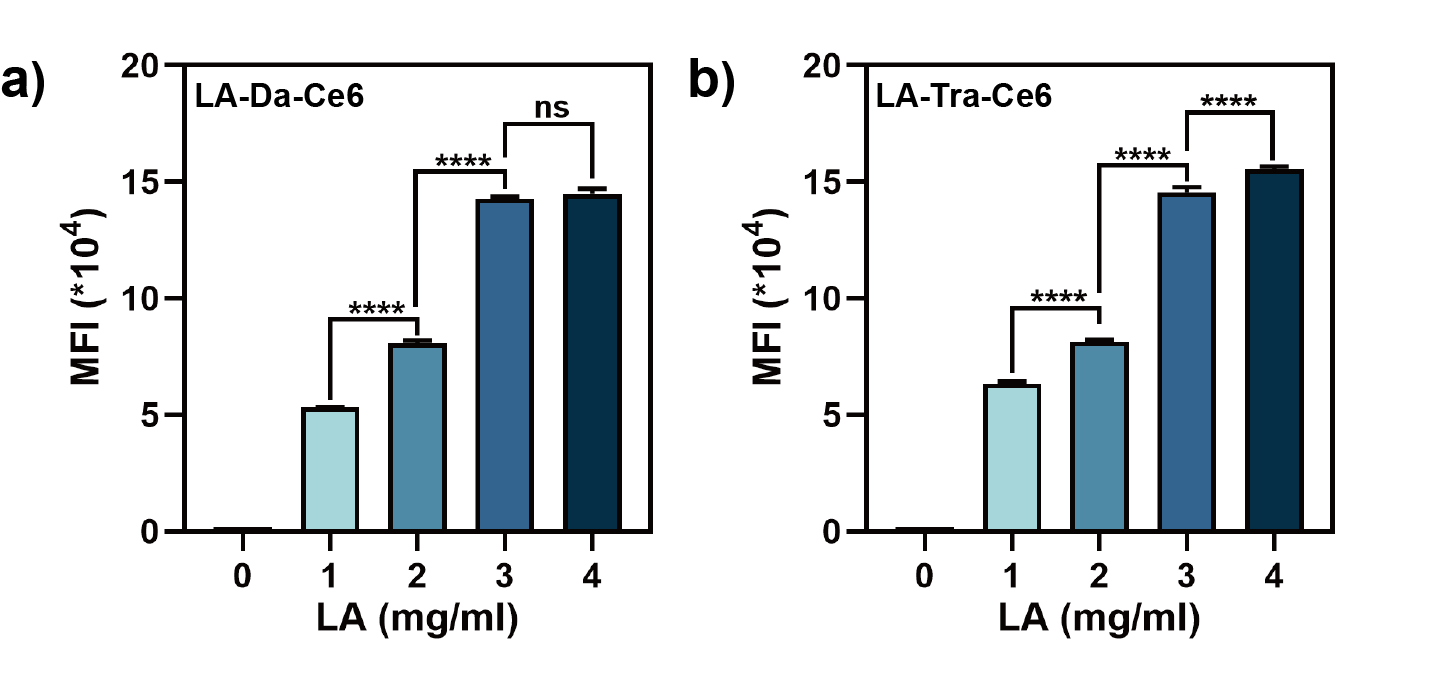


**Figure S2. Effect of LA content on drug uptake efficiency in tumor cells.** (A) Mean Fluorescence Intensity (MFI) of cells treated with LA-Da-Ce6 nanomedicines at varying concentrations of LA. (B) Mean Fluorescence Intensity (MFI) of cells treated with LA-Tra-Ce6 nanomedicines at varying concentrations of LA. Data are expressed as mean ± standard deviation (n = 3); t-test; ****p < 0.0001.


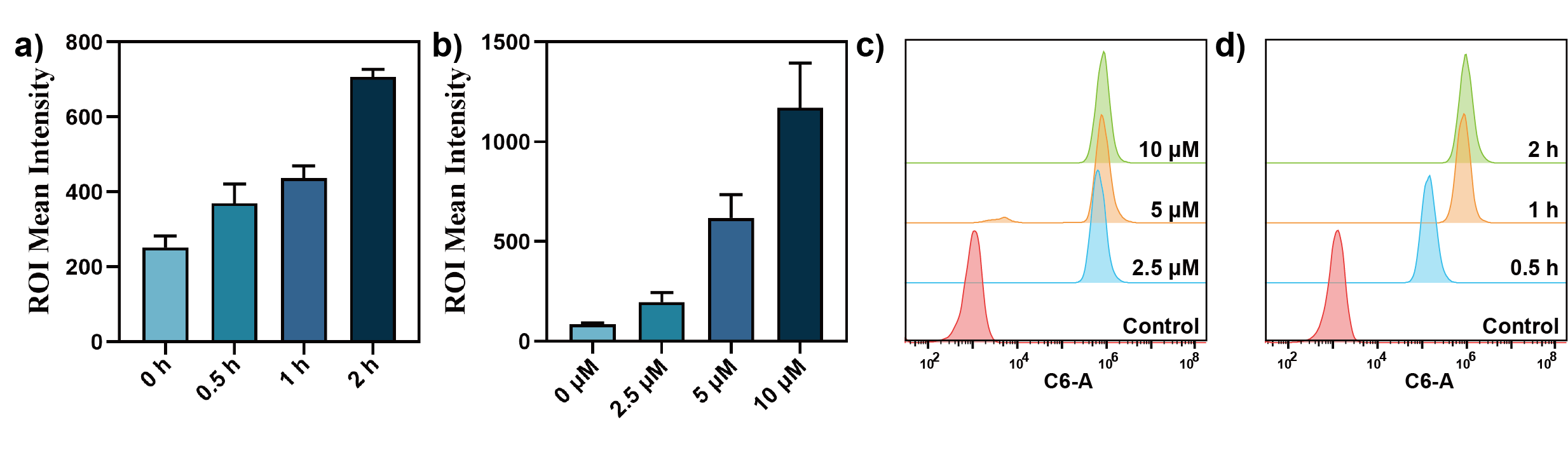


**Figure S3. Comprehensive investigation of DTL and related nanodrug uptake characteristics.** (a) Confocal Laser Scanning Microscopy (CLSM) analysis quantifying the mean fluorescence intensity (MFI) of Cy5-Mal-labeled DTL (Cy5@DTL) uptake by cells over time. (b) CLSM analysis quantifying the MFI of Cy5@DTL uptake by cells at different concentrations. (c) Flow cytometry (FCM) histograms showing the time-dependent cellular uptake of CTL (LA-based nanodrugs with Coumarin 6). (d) FCM histograms showing the concentration-dependent cellular uptake of CTL.


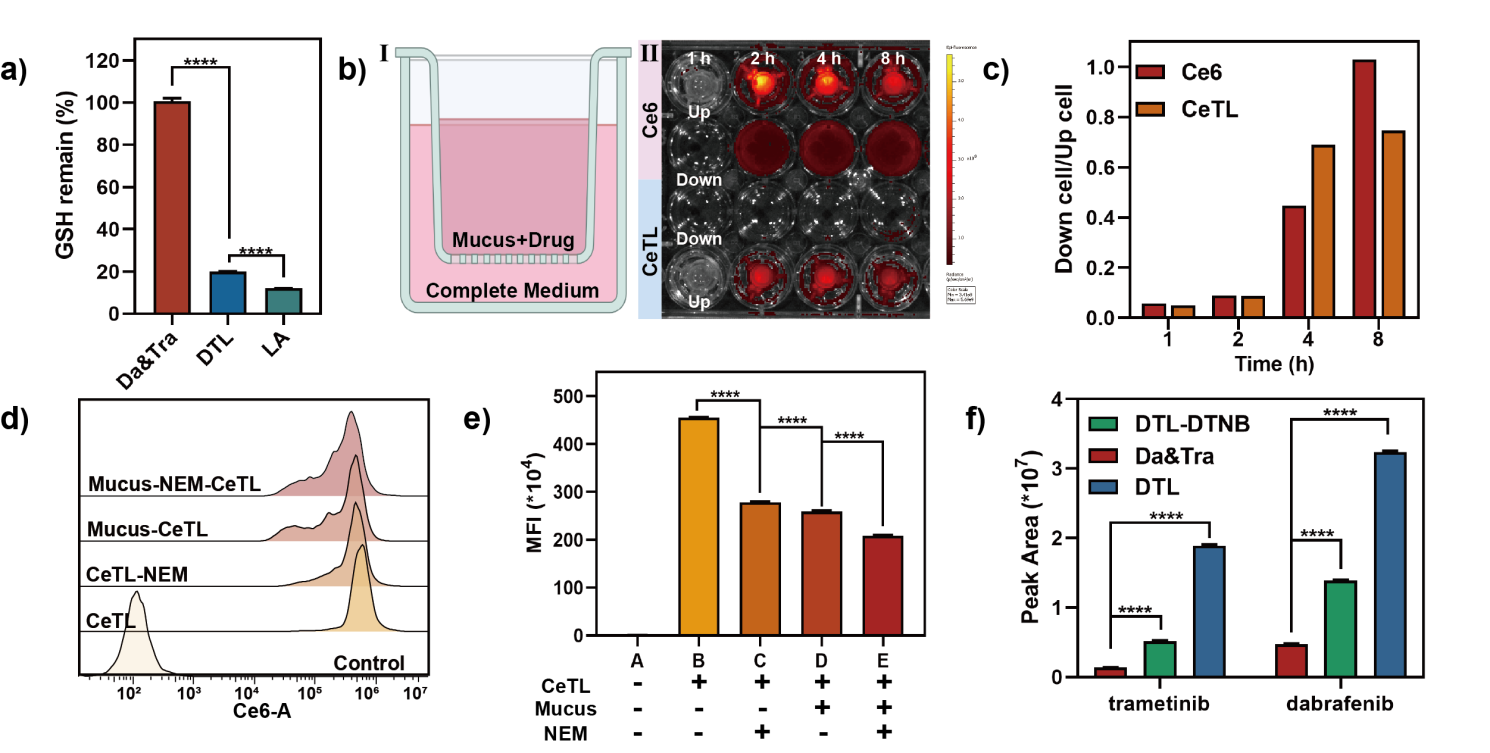


**Figure S4. DTL Nanodrug Mucus Retention and Mucosal Penetration Capabilities.** (A) Quantification of free thiols in DTL by Ellman’s assay. (B) Transwell model for mucoadhesion analysis. Ⅰ: the upper chamber contains a mixture of nanodrugs and mucus, while the lower chamber contains complete culture medium. Ⅱ: Fluorescence images of the upper and lower chambers after 1, 2, 4, and 8 h incubation. (C) Quantified fluorescence intensities derived from (B) (Ⅱ). (D) FCM analysis of mucus penetration with various treatments (CeTL, CeTL-NEM, Mucus-CeTL, Mucus-NEM-CeTL). (E) Quantitative comparison of intracellular fluorescence across groups in (D). (F) LC-MS/MS quantification of Da/Tra permeation across the rat mucus layer using Franz diffusion cells. Data are presented as mean ± standard deviation (n = 3); t-test; ****p < 0.0001.


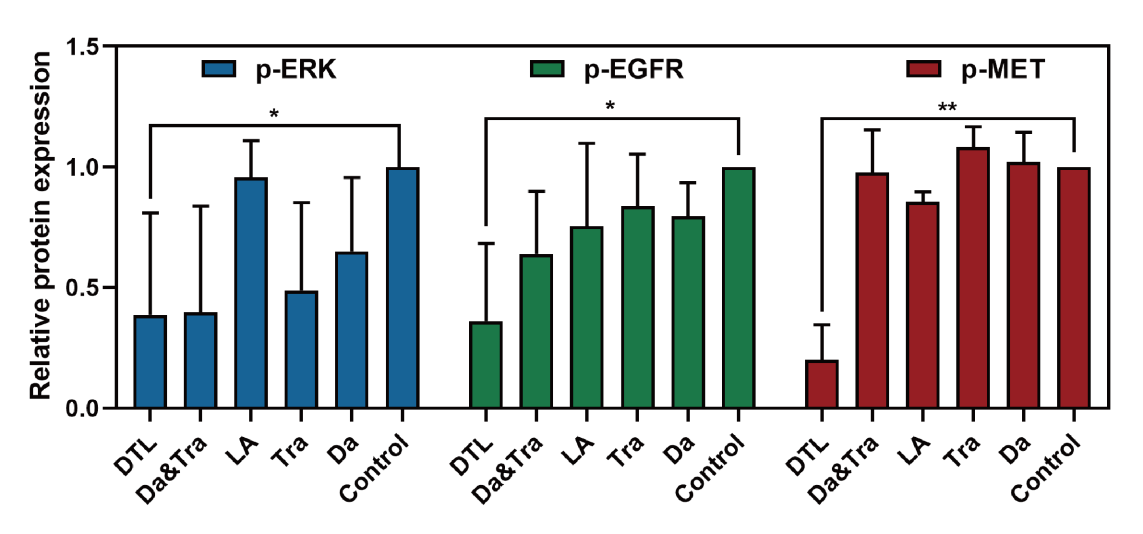


**Figure S5. Western blot (WB) analysis of tumor progression-related protein expression.**Bar graphs showing the relative protein expression levels of p-ERK, p-EGFR, and p-MET in cells treated with Control, LA, Da&Tra, and DTL groups. Data are expressed as mean ± standard deviation (n = 3); t-test; ns: no difference, *p < 0.05, **p < 0.01, ***p < 0.001.


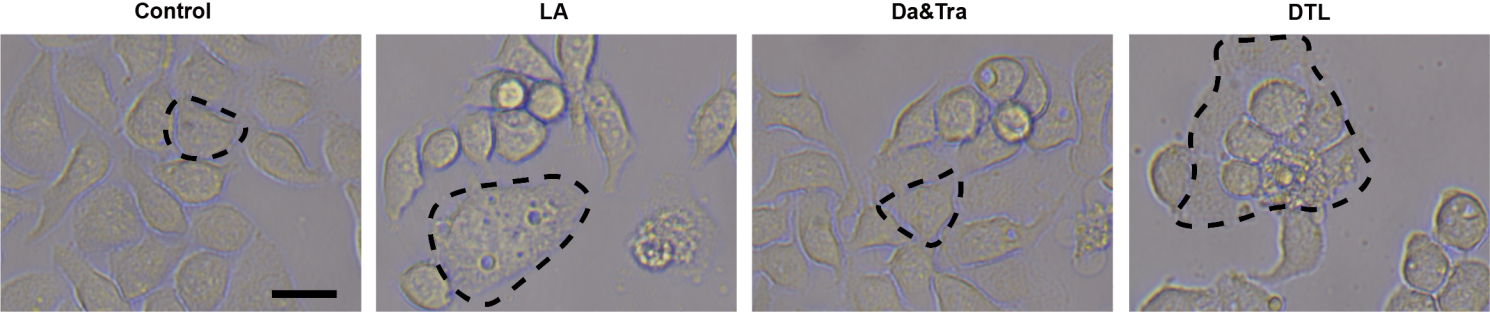


**Figure S6. Morphological changes in PC9 cells after treatment with different groups.** Inverted microscopy images showing morphological changes in PC9 cells after treatment with different groups, with areas of pore formation highlighted. Scale bar: 20 μm.


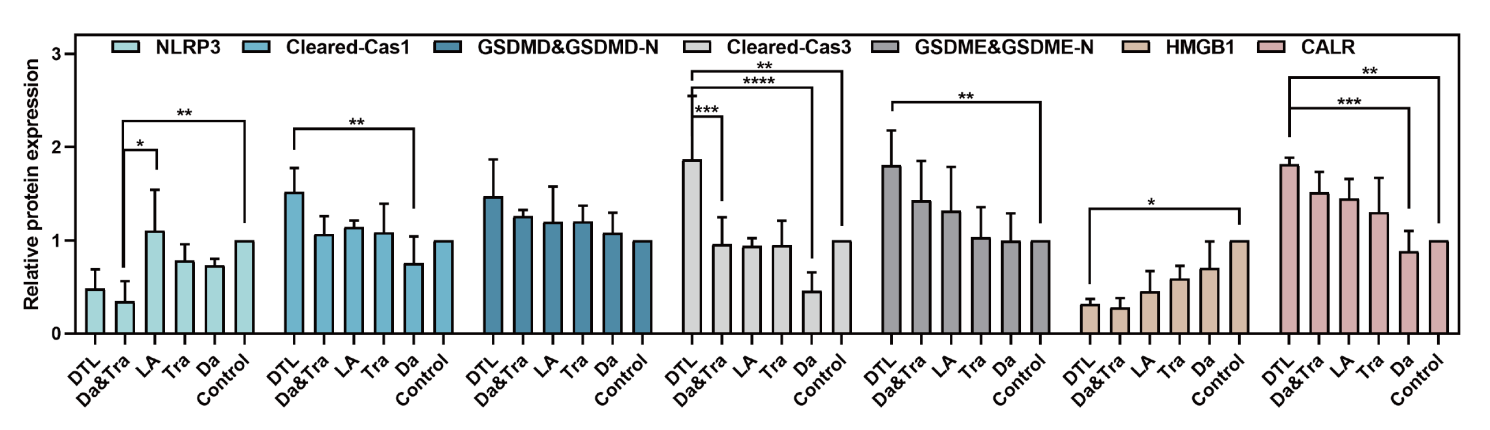


**Figure S7. Western blot (WB) analysis of pyroptosis-related pathway protein expression.** Bar graphs showing the relative protein expression levels of NLRP3, Cleaved-Caspase1, GSDMD&GSDMD-N, Cleaved-Caspase3, GSDME&GSDME-N, HMGB1, and CALR in cells treated with various groups. Data are expressed as mean ± standard deviation (n = 3); t-test; *p < 0.05, ***p < 0.001.


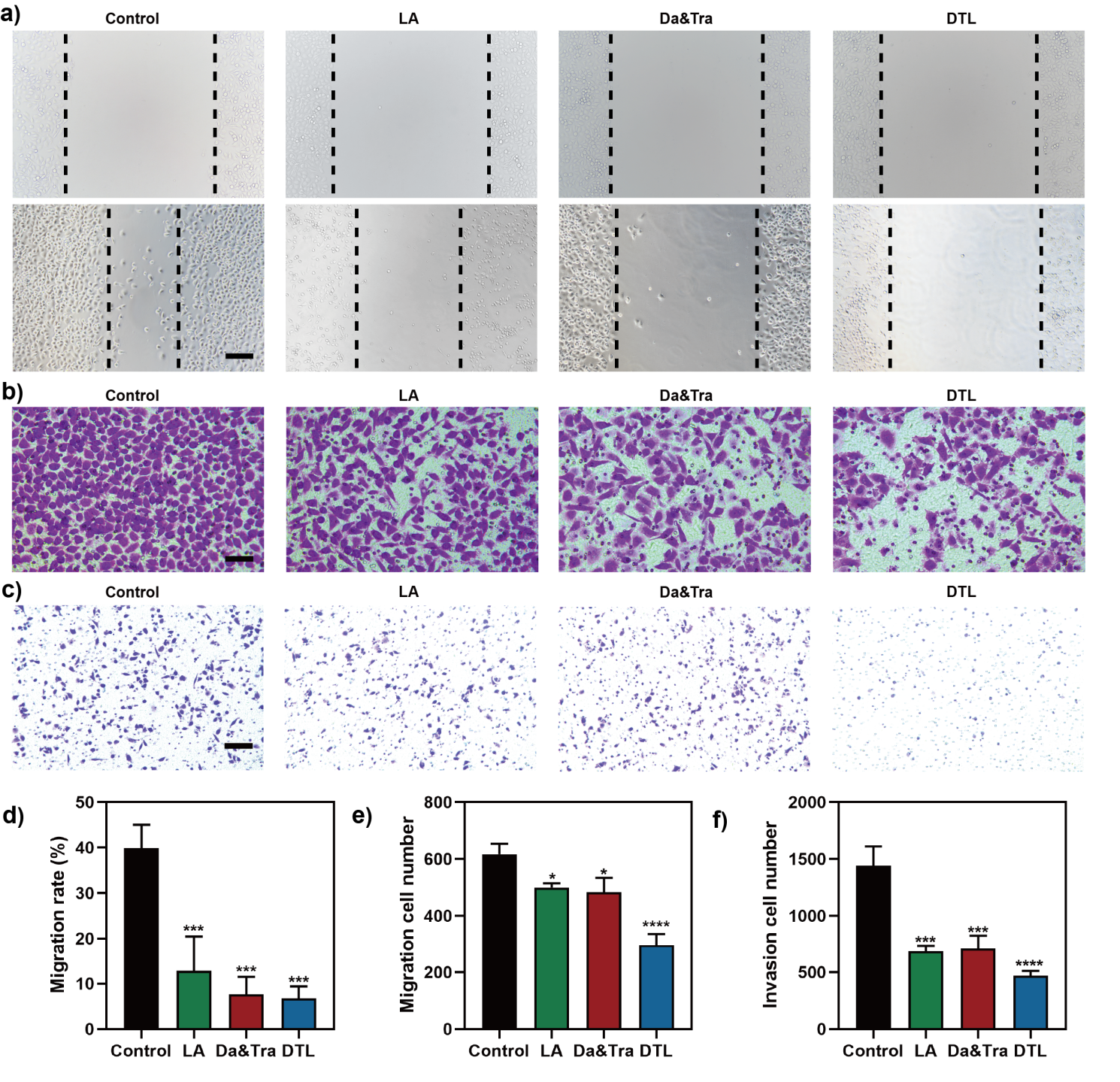


**Figure S8. Evaluation of DTL nanomedicines' anti-metastatic and anti-invasive capabilities in NSCLC.** (A) Representative images from scratch assays evaluating the migration ability of PC9 cells in different treatment groups. Scale bar: 200 μm. (B) Representative images from Transwell migration assays. Scale bar: 100 μm. (C) Representative images from Transwell invasion assays. Scale bar: 200 μm. (D) Statistical quantification of cell migration rate from scratch assays (panel A). (E) Statistical quantification of migrating cell numbers from Transwell migration assays (panel B). (F) Statistical quantification of invading cell numbers from Transwell invasion assays (panel C). Data are expressed as mean ± standard deviation (n = 3); t-test; *p < 0.05, ***p < 0.001, ****p < 0.0001.


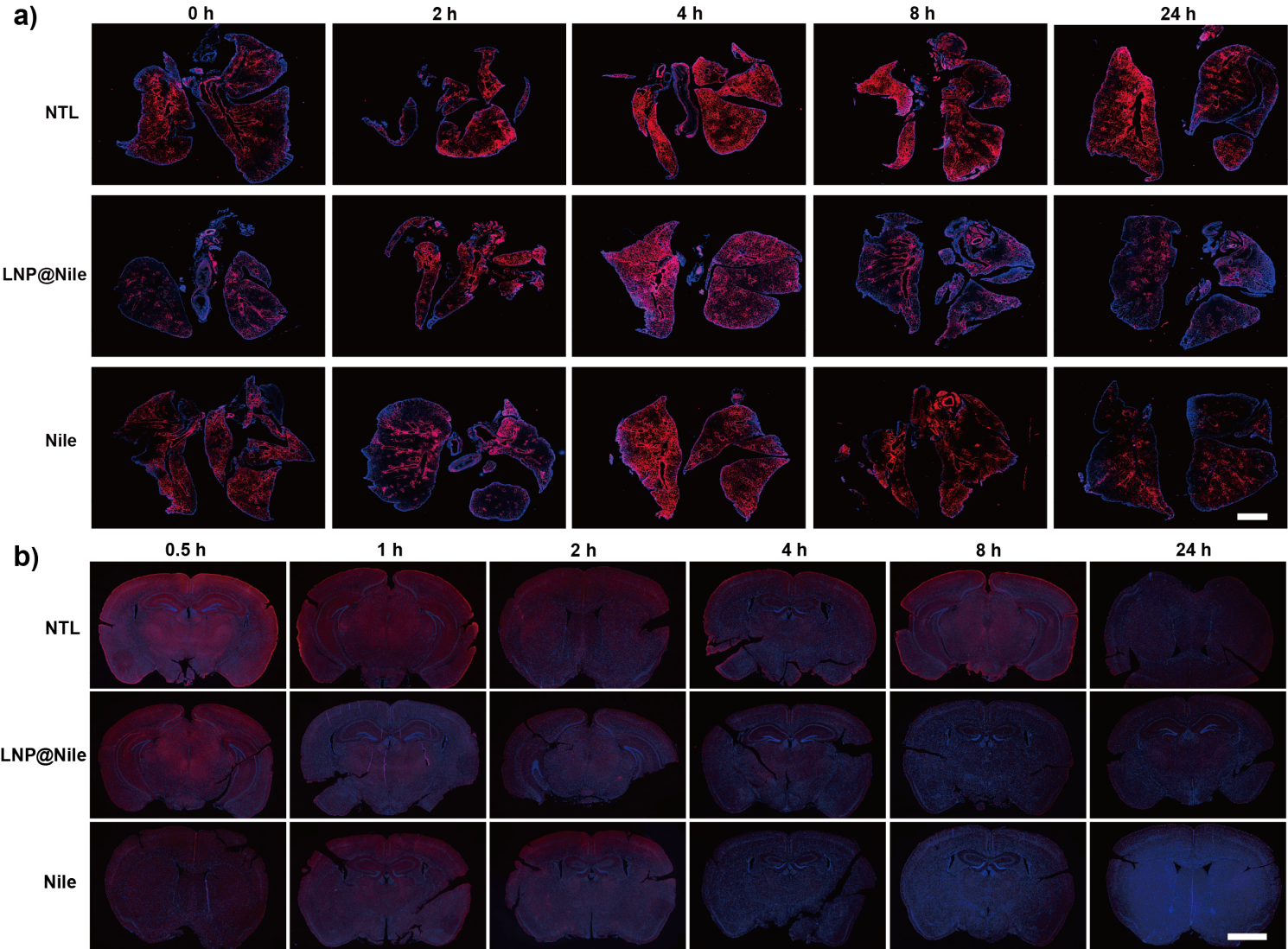


**Figure S9. Fluorescence imaging of tissue sections showing drug accumulation.** (A) Representative images of lung tissue sections from mice treated with NTL (Nile-labeled DTL), LNP@Nile (Nile-labeled lipid nanoparticles), or free Nile, at various time points post-intranasal administration. Scale bar: 2000 μm. (B) Representative images of brain tissue sections from mice treated with NTL, LNP@Nile, or free Nile, at various time points post-intranasal administration. Scale bar: 2000 μm. Blue indicates the cell nucleus (DAPI stain), and red indicates the corresponding drug fluorescence.


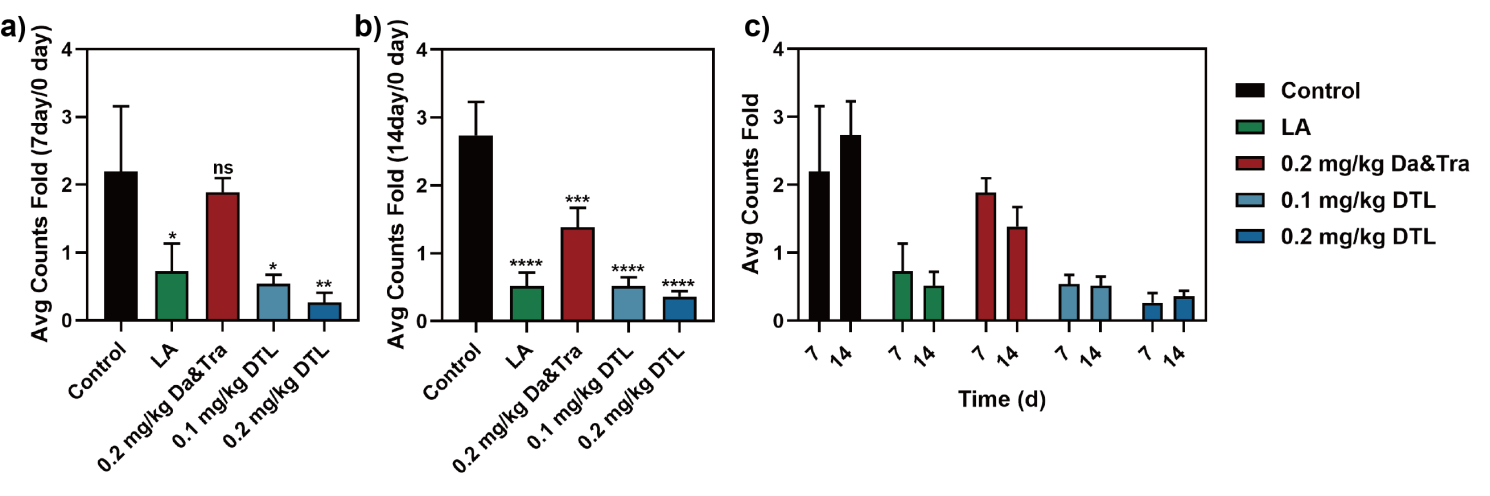


**Figure S10. Comparison of bioluminescence signal intensity in lung tumors. Comparison of bioluminescence signal intensity (tumor burden) in lung tumors across different treatment groups in an orthotopic NSCLC model.** (A) Ratio of bioluminescence signal intensity in lung tumors on day 7 of treatment compared to day 0. (B) Ratio of bioluminescence signal intensity on day 14 of treatment compared to day 0. (C) Combined comparison of data from panels (A) and (B). Results are expressed as mean ± standard deviation (n = 3); t-test; ns: no significant difference, *p < 0.05, **p < 0.01, ***p < 0.001, ****p < 0.0001.


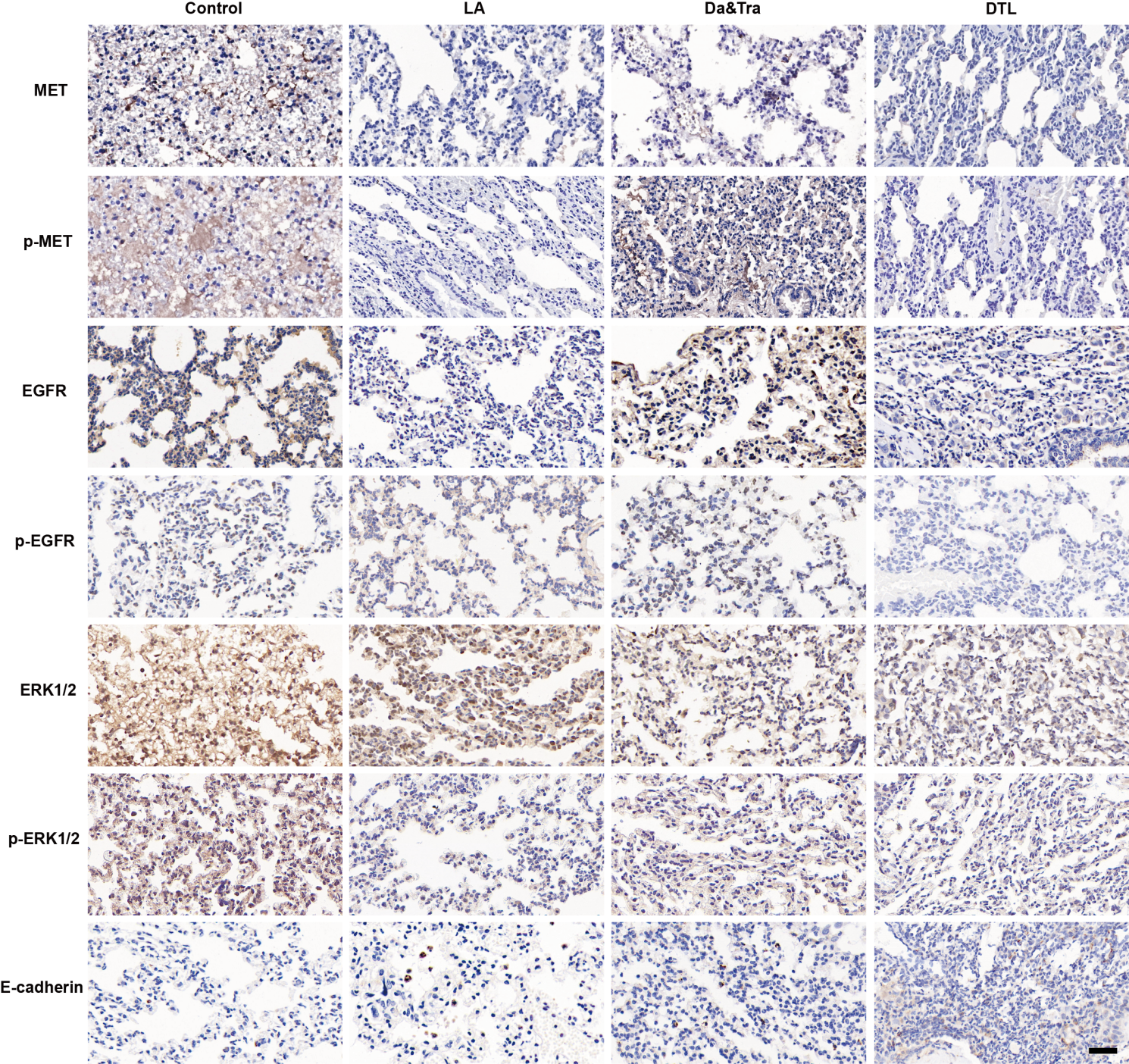


**Figure S11. Immunohistochemical analysis of DTL regulation on key signaling pathways.** Representative immunohistochemistry staining showing the expression levels of Met, p-Met, EGFR, p-EGFR, ERK1/2, p-ERK1/2, and E-cadherin in lung tumor tissue after different treatments. Scale bar: 100 μm. Blue indicates nuclear stain (hematoxylin), and brown indicates the corresponding protein (DAB chromogen).


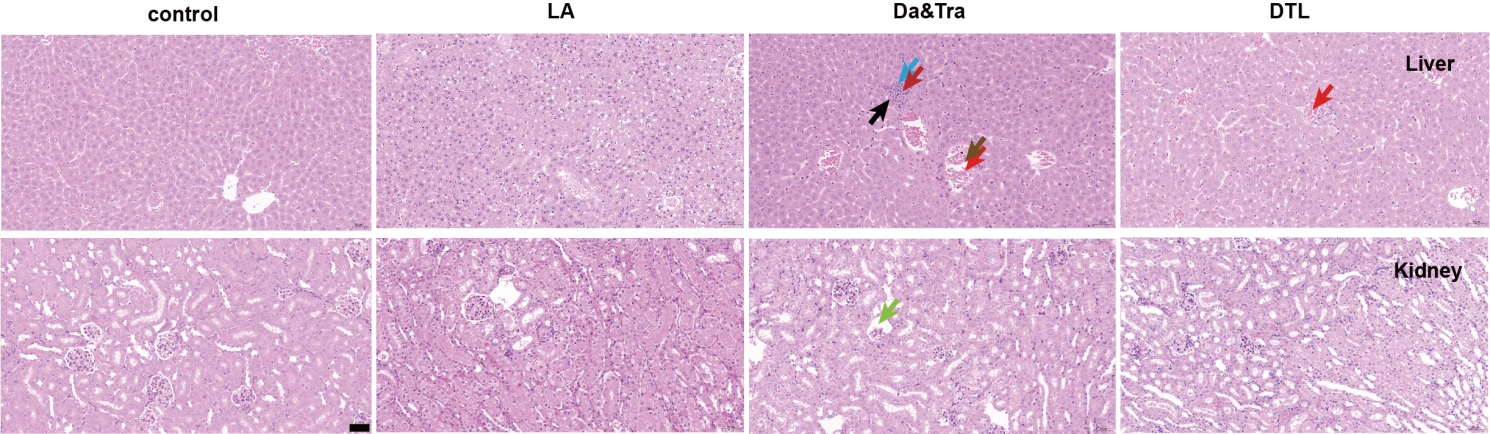


**Figure S12.** **Histopathological evaluation of liver and kidney tissues. Histopathological evaluation of liver and kidney tissues after intranasal administration of treatments in a PC9 lung cancer model via H&E staining.** Representative H&E-stained sections showing morphological changes in liver and kidney tissues across different treatment groups. Scale bar: 50 μm. Specific pathological features in the Da&Tra group are indicated by arrows: orange for venous congestion, black for focal hepatic cell necrosis, blue for granulocyte infiltration, dark red for fibrous tissue proliferation, brown for pigment deposition, and light green for eosinophilic material deposition within renal tubules.

**3. Supplementary Table**

| **CAS** | **Name** | **CAS** | **Name** |
| --- | --- | --- | --- |
| 1195765-45-7 | Dabrafenib | 379231-04-6 | Saracatinib |
| 137234-62-9 | Voriconazole | 380843-75-4 | Bosutinib |
| 21967-41-9 | Baicalin | 1110813-31-4 | Dacomitinib |
| 59-05-2 | Methoexate | 557795-19-4 | Sunitinib |
| 871700-17-3 | Trametinib | 1073154-85-4 | Defactinib |
| 19660-77-6 | Chlorin e6 | 525-66-6 | Propanolol |
| 23214-92-8 | Doxorubicin | 50-02-2 | dexamethasone |
| [14459-29-1](https://china.guidechem.com/114399/detail.html) | [Hematoporphyrin](https://www.guidechem.com/encyclopedia/hematoporphyrin-dic114399.html) | 318-98-9 | Propranolol Hydrochloride |
| 3599-32-4 | Indocyanine green | 50-78-2 | Aspirin |
| [934660-93-2](https://china.guidechem.com/1556922/detail.html) | [Cobimetinib](https://www.guidechem.com/encyclopedia/xl518-dic1556922.html) | 113665-84-2 | Clopidogrel |
| 184475-35-2 | Gefitinib | 150322-43-3 | Prasugrel |
| [95058-81-4](https://china.guidechem.com/28627/detail.html) | [Gemcitabine](https://www.guidechem.com/encyclopedia/gemcitabine-dic28627.html) | 274693-27-5 | Ticagrelor |
| [183321-74-6](https://china.guidechem.com/1554622/) | [Erlotinib](https://www.guidechem.com/encyclopedia/icotinib-dic1554622.html) | 73963-72-1 | Cilostazol |
| [918504-65-1](https://china.guidechem.com/1173516/) | [Vemurafenib](https://www.guidechem.com/encyclopedia/vemurafenib-dic1173516.html) | 63610-08-2 | Indobufen |
| [33069-62-4](https://china.guidechem.com/22415/) | [Paclitaxel](https://www.guidechem.com/encyclopedia/paclitaxel-dic22415.html) | 656247-17-5 | Nintedanib |
| [60-54-8](https://china.guidechem.com/279/) | [Tetracycline](https://www.guidechem.com/encyclopedia/tetracycline-dic279.html) | [58-32-2](https://china.guidechem.com/217/) | Dipyridamole |
| [7689-03-4](https://china.guidechem.com/15395/detail.html) | [Camptothecin](https://www.guidechem.com/encyclopedia/camptothecin-dic15395.html) | 443-48-1 | Metronidazole |
| 7385-67-3 | Nile Red | 103577-45-3 | Lansoprazole |
| 85622-93-1 | Temozolomide | 117976-89-3 | Rabeprazole |
| 19685-09-7 | 10-Hydroxycamptothecin | 70458-96-7 | Norfloxacin |
| 71125-38-7 | meloxicam | 51481-61-9 | cimetidine |
| 59277-89-3 | Acyclovir | 56-75-7 | Chloramphenicol |
| 86386-73-4 | Fluconazole | [7681-93-8](https://china.guidechem.com/15390/) | Natamycin |
| 426-13-1 | Fluorometholone | 52549-17-4 | pranoprofen |
| 114-07-8 | Erythromycin | 65277-42-1 | Ketoconazole |
| 57-62-5 | chlortetracycline | 32986-56-4 | Tobramycin |
| 81103-11-9 | Clarithromycin | 100986-85-4 | Levofloxacin |
| 13292-46-1 | Rifampin | 26787-78-0 | Amoxicillin |
| 73590-58-6 | Omeprazole | 67-45-8 | Furoxone |
| 57808-66-9 | Domperidone | 564-25-0 | Doxycycline |
| 76824-35-6 | Famotidine | 38215-36-0 | Coumarin 6 |
| 79-57-2 | Oxytetracycline |  |  |

**Table S1. List of 63 Commonly Used Small Molecule Drugs.**

| **Drug A &Drug B** | **A-size (nm)** | **A-PDI** | **B-size** | **B-PDI** | **Size (nm)** | **PDI** |
| --- | --- | --- | --- | --- | --- | --- |
| 1195765-45-7 & 7681-93-8 | 953.87 | 0.64 | 5014.67 | 0.26 | 200.73 | 0.05 |
| 1195765-45-7 & 19660-77-6 | 953.87 | 0.64 | 456.47 | 0.61 | 281.00 | 0.08 |
| 137234-62-9 & 918504-65-1 | 4169.33 | 1.00 | 136.93 | 0.16 | 135.17 | 0.05 |
| 137234-62-9 & 7681-93-8 | 4169.33 | 1.00 | 5014.67 | 0.26 | 138.87 | 0.02 |
| 21967-41-9 & 60-54-8 | 1035.33 | 0.36 | 2740.00 | 0.71 | 166.33 | 0.02 |
| 21967-41-9 & 50-02-2 | 1035.33 | 0.36 | 1354.23 | 0.73 | 123.30 | 0.03 |
| 21967-41-9 & 114-07-8 | 1035.33 | 0.36 | 837.87 | 0.76 | 295.07 | 0.03 |
| 21967-41-9 & 79-57-2 | 1035.33 | 0.36 | 677.63 | 0.62 | 169.90 | 0.03 |
| 21967-41-9 & 564-25-0 | 1035.33 | 0.36 | 1751.33 | 0.38 | 307.07 | 0.03 |
| 59-05-2 & 7681-93-8 | 1396.07 | 0.91 | 5014.67 | 0.26 | 170.67 | 0.04 |
| 59-05-2 & 95058-81-4 | 1396.07 | 0.91 | 1684.57 | 0.88 | 165.57 | 0.02 |
| 59-05-2 & 918504-65-1 | 1396.07 | 0.91 | 136.93 | 0.16 | 159.93 | 0.03 |
| 871700-17-3 & 137234-62-9 | 140.57 | 0.21 | 4169.33 | 1.00 | 129.50 | 0.02 |
| 871700-17-3 & 918504-65-1 | 140.57 | 0.21 | 136.93 | 0.16 | 127.40 | 0.02 |
| 871700-17-3 & 59-05-2 | 140.57 | 0.21 | 1396.07 | 0.91 | 147.57 | 0.03 |
| 871700-17-3 & 85622-93-1 | 140.57 | 0.21 | 213.13 | 0.71 | 133.03 | 0.04 |
| 871700-17-3 & 19660-77-6 | 140.57 | 0.21 | 456.47 | 0.61 | 248.27 | 0.03 |
| 871700-17-3 & 7385-67-3 | 140.57 | 0.21 | 2360.40 | 0.79 | 161.57 | 0.02 |
| 14459-29-1 & 19685-09-7 | 5000.67 | 0.30 | 1606.00 | 0.23 | 206.17 | 0.03 |
| 14459-29-1 & 1195765-45-7 | 5000.67 | 0.30 | 953.87 | 0.64 | 178.60 | 0.12 |
| 14459-29-1 & 871700-17-3 | 5000.67 | 0.30 | 140.57 | 0.21 | 216.13 | 0.08 |
| 14459-29-1 & 918504-65-1 | 5000.67 | 0.30 | 136.93 | 0.16 | 187.63 | 0.20 |
| 14459-29-1 & 184475-35-2 | 5000.67 | 0.30 | 1399.00 | 0.78 | 187.80 | 0.03 |
| 14459-29-1 & 183321-74-6 | 5000.67 | 0.30 | 9530.33 | 0.69 | 120.47 | 0.15 |
| 14459-29-1 & 934660-93-2 | 5000.67 | 0.30 | 2026.60 | 0.51 | 175.90 | 0.03 |
| 19660-77-6 & 60-54-8 | 456.47 | 0.61 | 2740.00 | 0.71 | 197.77 | 0.10 |
| 19660-77-6 & 3599-32-4 | 456.47 | 0.61 | 2886.87 | 1.00 | 118.10 | 0.23 |
| 19660-77-6 & 33069-62-4 | 456.47 | 0.61 | 6681.00 | 0.70 | 182.57 | 0.19 |
| 19660-77-6 & 59-05-2 | 456.47 | 0.61 | 1396.07 | 0.91 | 137.73 | 0.06 |
| 19660-77-6 & 19685-09-7 | 456.47 | 0.61 | 1606.00 | 0.23 | 221.83 | 0.03 |
| 19660-77-6 & 1195765-45-7 | 456.47 | 0.61 | 953.87 | 0.64 | 281.00 | 0.08 |
| 19660-77-6 & 871700-17-3 | 456.47 | 0.61 | 140.57 | 0.21 | 248.27 | 0.03 |
| 19660-77-6 & 184475-35-2 | 456.47 | 0.61 | 1399.00 | 0.78 | 188.57 | 0.13 |
| 19660-77-6 & 918504-65-1 | 456.47 | 0.61 | 136.93 | 0.16 | 236.10 | 0.06 |
| 19660-77-6 & 85622-93-1 | 456.47 | 0.61 | 213.13 | 0.71 | 206.03 | 0.02 |
| 3599-32-4 & 1195765-45-7 | 2886.87 | 1.00 | 953.87 | 0.64 | 326.73 | 0.25 |
| 3599-32-4 & 60-54-8 | 2886.87 | 1.00 | 2740.00 | 0.71 | 159.37 | 0.18 |
| 3599-32-4 & 871700-17-3 | 2886.87 | 1.00 | 140.57 | 0.21 | 254.67 | 0.10 |
| 3599-32-4 & 59-05-2 | 2886.87 | 1.00 | 1396.07 | 0.91 | 170.73 | 0.18 |
| 3599-32-4 & 183321-74-6 | 2886.87 | 1.00 | 9530.33 | 0.69 | 252.07 | 0.29 |
| 3599-32-4 & 934660-93-2 | 2886.87 | 1.00 | 2026.60 | 0.51 | 286.07 | 0.27 |
| 3599-32-4 & 184475-35-2 | 2886.87 | 1.00 | 1399.00 | 0.78 | 286.23 | 0.19 |
| 3599-32-4 & 918504-65-1 | 2886.87 | 1.00 | 136.93 | 0.16 | 166.07 | 0.18 |
| 50-78-2 & 274693-27-5 | 534.40 | 0.54 | 232.10 | 0.09 | 103.20 | 0.05 |
| 50-78-2 & 73963-72-1 | 534.40 | 0.54 | 1367.00 | 0.63 | 141.23 | 0.05 |
| 50-78-2 & 113665-84-2 | 534.40 | 0.54 | 460.10 | 0.32 | 282.30 | 0.04 |
| 50-78-2 & 656247-17-5 | 534.40 | 0.54 | 3728.00 | 0.34 | 101.03 | 0.11 |
| 113665-84-2 & 656247-17-5 | 460.10 | 0.32 | 3728.00 | 0.34 | 128.07 | 0.22 |
| 113665-84-2 & 63610-08-2 | 460.10 | 0.32 | 19016.00 | 0.61 | 146.97 | 0.09 |
| 113665-84-2 & 73963-72-1 | 460.10 | 0.32 | 1367.00 | 0.63 | 313.90 | 0.05 |
| 150322-43-3 & 656247-17-5 | 839.80 | 0.43 | 3728.00 | 0.34 | 125.20 | 0.15 |
| 150322-43-3 & 73963-72-1 | 839.80 | 0.43 | 1367.00 | 0.63 | 252.77 | 0.03 |
| 150322-43-3 & 63610-08-2 | 839.80 | 0.43 | 19016.00 | 0.61 | 209.53 | 0.05 |
| 274693-27-5 & 656247-17-5 | 232.10 | 0.09 | 3728.00 | 0.34 | 127.83 | 0.08 |
| 274693-27-5 & 73963-72-1 | 232.10 | 0.09 | 1367.00 | 0.63 | 153.73 | 0.19 |
| 274693-27-5 & 58-32-2 | 232.10 | 0.09 | 2549.00 | 0.95 | 138.70 | 0.13 |
| 63610-08-2 & 274693-27-5 | 19016.00 | 0.61 | 232.10 | 0.09 | 100.38 | 0.06 |
| 63610-08-2 & 73963-72-1 | 19016.00 | 0.61 | 1367.00 | 0.63 | 104.10 | 0.07 |
| 63610-08-2 & 656247-17-5 | 19016.00 | 0.61 | 3728.00 | 0.34 | 220.60 | 0.09 |
| 656247-17-5 & 58-32-2 | 3728.00 | 0.34 | 2549.00 | 0.95 | 114.67 | 0.13 |
| 656247-17-5 & 73963-72-1 | 3728.00 | 0.34 | 1367.00 | 0.63 | 162.13 | 0.25 |
| 26787-78-0 & 443-48-1 | 3629.70 | 0.92 | 5914.67 | 0.57 | 82.10 | 0.04 |
| 26787-78-0 & 73590-58-6 | 3629.70 | 0.92 | 321.90 | 0.24 | 160.87 | 0.06 |
| 26787-78-0 & 67-45-8 | 3629.70 | 0.92 | 2820.33 | 0.65 | 91.12 | 0.03 |
| 26787-78-0 & 76824-35-6 | 3629.70 | 0.92 | 3162.00 | 0.63 | 65.70 | 0.06 |
| 73590-58-6 & 67-45-8 | 321.90 | 0.24 | 2820.33 | 0.65 | 175.13 | 0.11 |
| 73590-58-6 & 103577-45-3 | 321.90 | 0.24 | 1192.67 | 0.08 | 221.27 | 0.03 |
| 73590-58-6 & 76824-35-6 | 321.90 | 0.24 | 3162.00 | 0.63 | 161.23 | 0.08 |
| 67-45-8 & 76824-35-6 | 2820.33 | 0.65 | 3162.00 | 0.63 | 110.13 | 0.04 |
| 76824-35-6 & 103577-45-3 | 3162.00 | 0.63 | 1192.67 | 0.08 | 169.37 | 0.11 |
| 443-48-1 & 73590-58-6 | 5914.67 | 0.57 | 321.90 | 0.24 | 175.07 | 0.11 |
| 51481-61-9 & 443-48-1 | 1943.67 | 0.98 | 5914.67 | 0.57 | 99.59 | 0.15 |
| 51481-61-9 & 73590-58-6 | 1943.67 | 0.98 | 321.90 | 0.24 | 157.80 | 0.03 |
| 51481-61-9 & 67-45-8 | 1943.67 | 0.98 | 2820.33 | 0.65 | 90.50 | 0.07 |
| 51481-61-9 & 103577-45-3 | 1943.67 | 0.98 | 1192.67 | 0.08 | 150.53 | 0.05 |
| 70458-96-7 & 103577-45-3 | 1719.00 | 0.84 | 1192.67 | 0.08 | 173.90 | 0.06 |
| 70458-96-7 & 76824-35-6 | 1719.00 | 0.84 | 3162.00 | 0.63 | 116.50 | 0.07 |
| 70458-96-7 & 67-45-8 | 1719.00 | 0.84 | 2820.33 | 0.65 | 132.23 | 0.02 |
| 70458-96-7 & 443-48-1 | 1719.00 | 0.84 | 5914.67 | 0.57 | 139.93 | 0.12 |
| 70458-96-7 & 73590-58-6 | 1719.00 | 0.84 | 321.90 | 0.24 | 198.67 | 0.04 |
| 70458-96-7 & 51481-61-9 | 1719.00 | 0.84 | 1943.67 | 0.98 | 144.90 | 0.03 |
| 70458-96-7 & 26787-78-0 | 1719.00 | 0.84 | 3629.70 | 0.92 | 95.85 | 0.21 |
| 117976-89-3 & 51481-61-9 | 521.03 | 0.70 | 1943.67 | 0.98 | 147.90 | 0.13 |
| 117976-89-3 & 59277-89-3 | 521.03 | 0.70 | 4287.33 | 0.56 | 110.30 | 0.04 |
| 117976-89-3 & 76824-35-6 | 521.03 | 0.70 | 3162.00 | 0.63 | 153.93 | 0.05 |
| 117976-89-3 & 67-45-8 | 521.03 | 0.70 | 2820.33 | 0.65 | 144.37 | 0.09 |
| 117976-89-3 & 443-48-1 | 521.03 | 0.70 | 5914.67 | 0.57 | 113.47 | 0.14 |
| 117976-89-3 & 103577-45-3 | 521.03 | 0.70 | 1192.67 | 0.08 | 195.90 | 0.10 |
| 13292-46-1 & 65277-42-1 | 929.80 | 0.59 | 2835.00 | 1.00 | 228.27 | 0.06 |
| 52549-17-4 & 56-75-7 | 3274.33 | 0.65 | 1721.57 | 0.53 | 112.43 | 0.06 |
| 52549-17-4 & 65277-42-1 | 3274.33 | 0.65 | 2835.00 | 1.00 | 113.57 | 0.01 |
| 52549-17-4 & 100986-85-4 | 3274.33 | 0.65 | 3040.00 | 1.00 | 153.87 | 0.03 |
| 52549-17-4 & 59277-89-3 | 3274.33 | 0.65 | 4287.33 | 0.56 | 98.07 | 0.08 |
| 52549-17-4 & 426-13-1 | 3274.33 | 0.65 | 70109.00 | 0.69 | 112.70 | 0.03 |
| 56-75-7 & 65277-42-1 | 1721.57 | 0.53 | 2835.00 | 1.00 | 149.23 | 0.03 |
| 56-75-7 & 100986-85-4 | 1721.57 | 0.53 | 3040.00 | 1.00 | 187.90 | 0.28 |
| 56-75-7 & 59277-89-3 | 1721.57 | 0.53 | 4287.33 | 0.56 | 102.93 | 0.01 |
| 56-75-7 & 426-13-1 | 1721.57 | 0.53 | 70109.00 | 0.69 | 96.11 | 0.10 |
| 65277-42-1 & 100986-85-4 | 2835.00 | 1.00 | 3040.00 | 1.00 | 285.07 | 0.03 |
| 65277-42-1 & 59277-89-3 | 2835.00 | 1.00 | 4287.33 | 0.56 | 275.90 | 0.02 |
| 65277-42-1 & 426-13-1 | 2835.00 | 1.00 | 70109.00 | 0.69 | 169.00 | 0.05 |
| 65277-42-1 & 32986-56-4 | 2835.00 | 1.00 | 2046.87 | 0.73 | 283.80 | 0.04 |
| 59277-89-3 & 426-13-1 | 4287.33 | 0.56 | 70109.00 | 0.69 | 111.37 | 0.16 |
| 7681-93-8 & 3599-32-4 | 5014.67 | 0.26 | 2886.87 | 1.00 | 144.97 | 0.20 |
| 525-66-6 & 656247-17-5 | 1355.47 | 0.44 | 3728.00 | 0.34 | 189.67 | 0.13 |
| 73590-58-6 & 73963-72-1 | 321.90 | 0.24 | 1367.00 | 0.63 | 239.67 | 0.08 |
| 73590-58-6 & 426-13-1 | 321.90 | 0.24 | 70109.00 | 0.69 | 282.83 | 0.10 |
| 73590-58-6 & 86386-73-4 | 321.90 | 0.24 | 716.57 | 0.55 | 275.30 | 0.13 |
| 73590-58-6 & 52549-17-4 | 321.90 | 0.24 | 3274.33 | 0.65 | 243.00 | 0.11 |
| 73590-58-6 & 59277-89-3 | 321.90 | 0.24 | 4287.33 | 0.56 | 269.27 | 0.17 |
| 73590-58-6 & 50-02-2 | 321.90 | 0.24 | 1354.23 | 0.73 | 287.57 | 0.13 |
| 73590-58-6 & 51481-61-9 | 321.90 | 0.24 | 1943.67 | 0.98 | 263.13 | 0.14 |
| 73590-58-6 & 443-48-1 | 321.90 | 0.24 | 5914.67 | 0.57 | 175.07 | 0.11 |
| 73590-58-6 & 3599-32-4 | 321.90 | 0.24 | 2886.87 | 1.00 | 121.17 | 0.11 |
| 73590-58-6 & 19660-77-6 | 321.90 | 0.24 | 456.47 | 0.61 | 216.73 | 0.06 |
| 73590-58-6 & 871700-17-3 | 321.90 | 0.24 | 140.57 | 0.21 | 273.87 | 0.12 |
| 73590-58-6 & 137234-62-9 | 321.90 | 0.24 | 4169.33 | 1.00 | 264.27 | 0.14 |
| 73590-58-6 & 7385-67-3 | 321.90 | 0.24 | 2360.40 | 0.79 | 235.50 | 0.06 |
| 73590-58-6 & 7689-03-4 | 321.90 | 0.24 | 1990.33 | 0.53 | 286.50 | 0.13 |
| 73590-58-6 & 60-54-8 | 321.90 | 0.24 | 2740.00 | 0.71 | 285.60 | 0.09 |
| 73590-58-6 & 33069-62-4 | 321.90 | 0.24 | 6681.00 | 0.70 | 209.23 | 0.07 |
| 73590-58-6 & 19685-09-7 | 321.90 | 0.24 | 1606.00 | 0.23 | 226.60 | 0.06 |
| 73590-58-6 & 85622-93-1 | 321.90 | 0.24 | 213.13 | 0.71 | 237.57 | 0.08 |
| 73590-58-6 & 50-78-2 | 321.90 | 0.24 | 534.40 | 0.54 | 256.53 | 0.10 |
| 73590-58-6 & 274693-27-5 | 321.90 | 0.24 | 232.10 | 0.09 | 246.37 | 0.07 |
| 73590-58-6 & 63610-08-2 | 321.90 | 0.24 | 19016.00 | 0.61 | 229.33 | 0.13 |
| 426-13-1 & 67-45-8 | 70109.00 | 0.69 | 2820.33 | 0.65 | 120.47 | 0.09 |
| 426-13-1 & 443-48-1 | 70109.00 | 0.69 | 5914.67 | 0.57 | 94.11 | 0.17 |
| 426-13-1 & 21967-41-9 | 70109.00 | 0.69 | 1035.33 | 0.36 | 110.17 | 0.17 |
| 426-13-1 & 137234-62-9 | 70109.00 | 0.69 | 4169.33 | 1.00 | 99.87 | 0.11 |
| 426-13-1 & 117976-89-3 | 70109.00 | 0.69 | 521.03 | 0.70 | 118.70 | 0.17 |
| 426-13-1 & 7689-03-4 | 70109.00 | 0.69 | 1990.33 | 0.53 | 112.90 | 0.17 |
| 426-13-1 & 60-54-8 | 70109.00 | 0.69 | 2740.00 | 0.71 | 164.43 | 0.17 |
| 426-13-1 & 918504-65-1 | 70109.00 | 0.69 | 136.93 | 0.16 | 128.13 | 0.18 |
| 426-13-1 & 95058-81-4 | 70109.00 | 0.69 | 1684.57 | 0.88 | 123.57 | 0.18 |
| 426-13-1 & 1073154-85-4 | 70109.00 | 0.69 | 5538.33 | 0.92 | 253.07 | 0.07 |
| 426-13-1 & 71125-38-7 | 70109.00 | 0.69 | 533.93 | 0.38 | 109.60 | 0.14 |
| 426-13-1 & 19685-09-7 | 70109.00 | 0.69 | 1606.00 | 0.23 | 108.97 | 0.08 |
| 426-13-1 & 85622-93-1 | 70109.00 | 0.69 | 213.13 | 0.71 | 109.60 | 0.12 |
| 426-13-1 & 50-78-2 | 70109.00 | 0.69 | 534.40 | 0.54 | 109.33 | 0.07 |
| 426-13-1 & 73963-72-1 | 70109.00 | 0.69 | 1367.00 | 0.63 | 123.27 | 0.09 |
| 426-13-1 & 656247-17-5 | 70109.00 | 0.69 | 3728.00 | 0.34 | 256.10 | 0.13 |
| 59277-89-3 & 443-48-1 | 4287.33 | 0.56 | 5914.67 | 0.57 | 110.67 | 0.07 |
| 59277-89-3 & 76824-35-6 | 4287.33 | 0.56 | 3162.00 | 0.63 | 121.40 | 0.04 |
| 59277-89-3 & 57-62-5 | 4287.33 | 0.56 | 1303.00 | 0.42 | 204.87 | 0.04 |
| 59277-89-3 & 70458-96-7 | 4287.33 | 0.56 | 1719.00 | 0.84 | 159.90 | 0.12 |
| 59277-89-3 & 117976-89-3 | 4287.33 | 0.56 | 521.03 | 0.70 | 110.30 | 0.04 |
| 59277-89-3 & 137234-62-9 | 4287.33 | 0.56 | 4169.33 | 1.00 | 109.37 | 0.09 |
| 59277-89-3 & 21967-41-9 | 4287.33 | 0.56 | 1035.33 | 0.36 | 106.93 | 0.07 |
| 59277-89-3 & 59-05-2 | 4287.33 | 0.56 | 1396.07 | 0.91 | 141.00 | 0.03 |
| 59277-89-3 & 871700-17-3 | 4287.33 | 0.56 | 140.57 | 0.21 | 114.17 | 0.09 |
| 59277-89-3 & 918504-65-1 | 4287.33 | 0.56 | 136.93 | 0.16 | 125.80 | 0.03 |
| 59277-89-3 & 95058-81-4 | 4287.33 | 0.56 | 1684.57 | 0.88 | 118.83 | 0.06 |
| 59277-89-3 & 85622-93-1 | 4287.33 | 0.56 | 213.13 | 0.71 | 116.37 | 0.04 |
| 59277-89-3 & 60-54-8 | 4287.33 | 0.56 | 2740.00 | 0.71 | 142.70 | 0.03 |
| 59277-89-3 & 7385-67-3 | 4287.33 | 0.56 | 2360.40 | 0.79 | 192.07 | 0.03 |
| 59277-89-3 & 71125-38-7 | 4287.33 | 0.56 | 533.93 | 0.38 | 121.63 | 0.05 |
| 59277-89-3 & 274693-27-5 | 4287.33 | 0.56 | 232.10 | 0.09 | 118.37 | 0.10 |
| 59277-89-3 & 73963-72-1 | 4287.33 | 0.56 | 1367.00 | 0.63 | 126.57 | 0.03 |
| 59277-89-3 & 19685-09-7 | 4287.33 | 0.56 | 1606.00 | 0.23 | 120.97 | 0.07 |
| 59277-89-3 & 150322-43-3 | 4287.33 | 0.56 | 839.80 | 0.43 | 126.90 | 0.08 |
| 59277-89-3 & 1073154-85-4 | 4287.33 | 0.56 | 5538.33 | 0.92 | 237.77 | 0.02 |
| 114-07-8 & 19660-77-6 | 837.87 | 0.76 | 456.47 | 0.61 | 242.60 | 0.01 |
| 443-48-1 & 67-45-8 | 5914.67 | 0.57 | 2820.33 | 0.65 | 104.57 | 0.13 |
| 443-48-1 & 103577-45-3 | 5914.67 | 0.57 | 1192.67 | 0.08 | 210.87 | 0.09 |
| 443-48-1 & 51481-61-9 | 5914.67 | 0.57 | 1943.67 | 0.98 | 99.59 | 0.15 |
| 443-48-1 & 50-02-2 | 5914.67 | 0.57 | 1354.23 | 0.73 | 109.30 | 0.20 |
| 443-48-1 & 76824-35-6 | 5914.67 | 0.57 | 3162.00 | 0.63 | 112.90 | 0.14 |
| 443-48-1 & 70458-96-7 | 5914.67 | 0.57 | 1719.00 | 0.84 | 139.93 | 0.12 |
| 443-48-1 & 117976-89-3 | 5914.67 | 0.57 | 521.03 | 0.70 | 161.00 | 0.12 |
| 443-48-1 & 1195765-45-7 | 5914.67 | 0.57 | 953.87 | 0.64 | 273.97 | 0.13 |
| 443-48-1 & 137234-62-9 | 5914.67 | 0.57 | 4169.33 | 1.00 | 105.72 | 0.19 |
| 443-48-1 & 59-05-2 | 5914.67 | 0.57 | 1396.07 | 0.91 | 123.07 | 0.11 |
| 443-48-1 & 871700-17-3 | 5914.67 | 0.57 | 140.57 | 0.21 | 117.07 | 0.15 |
| 114-07-8 & 73963-72-1 | 837.87 | 0.76 | 1367.00 | 0.63 | 297.33 | 0.09 |
| 443-48-1 & 656247-17-5 | 5914.67 | 0.57 | 3728.00 | 0.34 | 137.13 | 0.13 |
| 443-48-1 & 63610-08-2 | 5914.67 | 0.57 | 19016.00 | 0.61 | 95.55 | 0.10 |
| 443-48-1 & 274693-27-5 | 5914.67 | 0.57 | 232.10 | 0.09 | 107.00 | 0.13 |
| 443-48-1 & 1073154-85-4 | 5914.67 | 0.57 | 5538.33 | 0.92 | 205.60 | 0.06 |
| 443-48-1 & 379231-04-6 | 5914.67 | 0.57 | 1374.87 | 0.76 | 312.43 | 0.19 |
| 443-48-1 & 71125-38-7 | 5914.67 | 0.57 | 533.93 | 0.38 | 102.30 | 0.12 |
| 443-48-1 & 19685-09-7 | 5914.67 | 0.57 | 1606.00 | 0.23 | 100.02 | 0.21 |
| 443-48-1 & 7385-67-3 | 5914.67 | 0.57 | 2360.40 | 0.79 | 207.60 | 0.03 |
| 443-48-1 & 60-54-8 | 5914.67 | 0.57 | 2740.00 | 0.71 | 121.17 | 0.07 |
| 443-48-1 & 33069-62-4 | 5914.67 | 0.57 | 6681.00 | 0.70 | 111.73 | 0.20 |
| 443-48-1 & 918504-65-1 | 5914.67 | 0.57 | 136.93 | 0.16 | 106.03 | 0.13 |
| 443-48-1 & 95058-81-4 | 5914.67 | 0.57 | 1684.57 | 0.88 | 94.48 | 0.10 |
| 26787-78-0 & 3599-32-4 | 3629.70 | 0.92 | 2886.87 | 1.00 | 238.13 | 0.13 |
| 26787-78-0 & 95058-81-4 | 3629.70 | 0.92 | 1684.57 | 0.88 | 267.77 | 0.19 |
| 26787-78-0 & 19660-77-6 | 3629.70 | 0.92 | 456.47 | 0.61 | 229.40 | 0.13 |
| 26787-78-0 & 21967-41-9 | 3629.70 | 0.92 | 1035.33 | 0.36 | 211.33 | 0.17 |
| 26787-78-0 & 137234-62-9 | 3629.70 | 0.92 | 4169.33 | 1.00 | 289.20 | 0.15 |
| 26787-78-0 & 117976-89-3 | 3629.70 | 0.92 | 521.03 | 0.70 | 126.80 | 0.06 |
| 26787-78-0 & 86386-73-4 | 3629.70 | 0.92 | 716.57 | 0.55 | 298.67 | 0.08 |
| 26787-78-0 & 52549-17-4 | 3629.70 | 0.92 | 3274.33 | 0.65 | 248.07 | 0.12 |
| 26787-78-0 & 50-02-2 | 3629.70 | 0.92 | 1354.23 | 0.73 | 311.53 | 0.25 |
| 26787-78-0 & 51481-61-9 | 3629.70 | 0.92 | 1943.67 | 0.98 | 79.03 | 0.05 |
| 26787-78-0 & 56-75-7 | 3629.70 | 0.92 | 1721.57 | 0.53 | 335.23 | 0.19 |
| 26787-78-0 & 60-54-8 | 3629.70 | 0.92 | 2740.00 | 0.71 | 244.70 | 0.23 |
| 26787-78-0 & 918504-65-1 | 3629.70 | 0.92 | 136.93 | 0.16 | 180.50 | 0.13 |
| 26787-78-0 & 85622-93-1 | 3629.70 | 0.92 | 213.13 | 0.71 | 146.83 | 0.23 |
| 26787-78-0 & 71125-38-7 | 3629.70 | 0.92 | 533.93 | 0.38 | 158.03 | 0.21 |
| 26787-78-0 & 1073154-85-4 | 3629.70 | 0.92 | 5538.33 | 0.92 | 309.50 | 0.03 |
| 26787-78-0 & 50-78-2 | 3629.70 | 0.92 | 534.40 | 0.54 | 119.27 | 0.01 |
| 26787-78-0 & 274693-27-5 | 3629.70 | 0.92 | 232.10 | 0.09 | 104.93 | 0.04 |
| 26787-78-0 & 63610-08-2 | 3629.70 | 0.92 | 19016.00 | 0.61 | 124.77 | 0.02 |
| 100986-85-4 & 67-45-8 | 3040.00 | 1.00 | 2820.33 | 0.65 | 159.27 | 0.06 |
| 100986-85-4 & 51481-61-9 | 3040.00 | 1.00 | 1943.67 | 0.98 | 178.37 | 0.02 |
| 100986-85-4 & 50-02-2 | 3040.00 | 1.00 | 1354.23 | 0.73 | 164.37 | 0.01 |
| 100986-85-4 & 70458-96-7 | 3040.00 | 1.00 | 1719.00 | 0.84 | 241.17 | 0.02 |
| 100986-85-4 & 1195765-45-7 | 3040.00 | 1.00 | 953.87 | 0.64 | 322.23 | 0.04 |
| 100986-85-4 & 137234-62-9 | 3040.00 | 1.00 | 4169.33 | 1.00 | 161.43 | 0.02 |
| 100986-85-4 & 21967-41-9 | 3040.00 | 1.00 | 1035.33 | 0.36 | 261.17 | 0.03 |
| 100986-85-4 & 59-05-2 | 3040.00 | 1.00 | 1396.07 | 0.91 | 228.63 | 0.03 |
| 100986-85-4 & 871700-17-3 | 3040.00 | 1.00 | 140.57 | 0.21 | 193.10 | 0.02 |
| 100986-85-4 & 19660-77-6 | 3040.00 | 1.00 | 456.47 | 0.61 | 199.80 | 0.06 |
| 100986-85-4 & 95058-81-4 | 3040.00 | 1.00 | 1684.57 | 0.88 | 153.90 | 0.04 |
| 100986-85-4 & 918504-65-1 | 3040.00 | 1.00 | 136.93 | 0.16 | 158.23 | 0.02 |
| 100986-85-4 & 33069-62-4 | 3040.00 | 1.00 | 6681.00 | 0.70 | 182.43 | 0.10 |
| 100986-85-4 & 60-54-8 | 3040.00 | 1.00 | 2740.00 | 0.71 | 182.93 | 0.02 |
| 100986-85-4 & 7689-03-4 | 3040.00 | 1.00 | 1990.33 | 0.53 | 170.20 | 0.03 |
| 100986-85-4 & 7385-67-3 | 3040.00 | 1.00 | 2360.40 | 0.79 | 233.23 | 0.09 |
| 100986-85-4 & 85622-93-1 | 3040.00 | 1.00 | 213.13 | 0.71 | 175.27 | 0.03 |
| 100986-85-4 & 19685-09-7 | 3040.00 | 1.00 | 1606.00 | 0.23 | 168.07 | 0.02 |
| 100986-85-4 & 71125-38-7 | 3040.00 | 1.00 | 533.93 | 0.38 | 143.27 | 0.02 |
| 100986-85-4 & 656247-17-5 | 3040.00 | 1.00 | 3728.00 | 0.34 | 169.93 | 0.08 |
| 100986-85-4 & 63610-08-2 | 3040.00 | 1.00 | 19016.00 | 0.61 | 131.80 | 0.01 |
| 100986-85-4 & 73963-72-1 | 3040.00 | 1.00 | 1367.00 | 0.63 | 133.10 | 0.04 |
| 100986-85-4 & 274693-27-5 | 3040.00 | 1.00 | 232.10 | 0.09 | 122.27 | 0.03 |
| 100986-85-4 & 50-78-2 | 3040.00 | 1.00 | 534.40 | 0.54 | 134.43 | 0.03 |
| 100986-85-4 & 1073154-85-4 | 3040.00 | 1.00 | 5538.33 | 0.92 | 253.80 | 0.03 |
| 67-45-8 & 73963-72-1 | 2820.33 | 0.65 | 1367.00 | 0.63 | 119.30 | 0.13 |
| 67-45-8 & 63610-08-2 | 2820.33 | 0.65 | 19016.00 | 0.61 | 108.33 | 0.09 |
| 67-45-8 & 656247-17-5 | 2820.33 | 0.65 | 3728.00 | 0.34 | 201.13 | 0.14 |
| 67-45-8 & 274693-27-5 | 2820.33 | 0.65 | 232.10 | 0.09 | 150.57 | 0.18 |
| 67-45-8 & 50-78-2 | 2820.33 | 0.65 | 534.40 | 0.54 | 118.53 | 0.03 |
| 67-45-8 & 1073154-85-4 | 2820.33 | 0.65 | 5538.33 | 0.92 | 162.83 | 0.03 |
| 67-45-8 & 71125-38-7 | 2820.33 | 0.65 | 533.93 | 0.38 | 112.40 | 0.07 |
| 67-45-8 & 19685-09-7 | 2820.33 | 0.65 | 1606.00 | 0.23 | 123.83 | 0.10 |
| 67-45-8 & 85622-93-1 | 2820.33 | 0.65 | 213.13 | 0.71 | 122.63 | 0.06 |
| 67-45-8 & 7385-67-3 | 2820.33 | 0.65 | 2360.40 | 0.79 | 129.30 | 0.08 |
| 67-45-8 & 918504-65-1 | 2820.33 | 0.65 | 136.93 | 0.16 | 116.00 | 0.07 |
| 67-45-8 & 95058-81-4 | 2820.33 | 0.65 | 1684.57 | 0.88 | 125.43 | 0.09 |
| 67-45-8 & 19660-77-6 | 2820.33 | 0.65 | 456.47 | 0.61 | 184.20 | 0.04 |
| 67-45-8 & 871700-17-3 | 2820.33 | 0.65 | 140.57 | 0.21 | 124.60 | 0.05 |
| 67-45-8 & 59-05-2 | 2820.33 | 0.65 | 1396.07 | 0.91 | 145.87 | 0.03 |
| 67-45-8 & 21967-41-9 | 2820.33 | 0.65 | 1035.33 | 0.36 | 139.57 | 0.13 |
| 67-45-8 & 1195765-45-7 | 2820.33 | 0.65 | 953.87 | 0.64 | 256.00 | 0.06 |
| 67-45-8 & 86386-73-4 | 2820.33 | 0.65 | 716.57 | 0.55 | 110.13 | 0.02 |
| 67-45-8 & 52549-17-4 | 2820.33 | 0.65 | 3274.33 | 0.65 | 111.90 | 0.04 |
| 67-45-8 & 103577-45-3 | 2820.33 | 0.65 | 1192.67 | 0.08 | 156.57 | 0.09 |
| 67-45-8 & 56-75-7 | 2820.33 | 0.65 | 1721.57 | 0.53 | 114.63 | 0.06 |
| 56-75-7 & 51481-61-9 | 1721.57 | 0.53 | 1943.67 | 0.98 | 100.05 | 0.31 |
| 56-75-7 & 50-02-2 | 1721.57 | 0.53 | 1354.23 | 0.73 | 90.52 | 0.30 |
| 56-75-7 & 76824-35-6 | 1721.57 | 0.53 | 3162.00 | 0.63 | 99.90 | 0.14 |
| 56-75-7 & 70458-96-7 | 1721.57 | 0.53 | 1719.00 | 0.84 | 134.27 | 0.11 |
| 56-75-7 & 1195765-45-7 | 1721.57 | 0.53 | 953.87 | 0.64 | 256.53 | 0.12 |
| 56-75-7 & 137234-62-9 | 1721.57 | 0.53 | 4169.33 | 1.00 | 96.40 | 0.17 |
| 56-75-7 & 21967-41-9 | 1721.57 | 0.53 | 1035.33 | 0.36 | 94.32 | 0.13 |
| 56-75-7 & 59-05-2 | 1721.57 | 0.53 | 1396.07 | 0.91 | 121.73 | 0.08 |
| 56-75-7 & 871700-17-3 | 1721.57 | 0.53 | 140.57 | 0.21 | 105.93 | 0.14 |
| 56-75-7 & 3599-32-4 | 1721.57 | 0.53 | 2886.87 | 1.00 | 129.23 | 0.30 |
| 56-75-7 & 95058-81-4 | 1721.57 | 0.53 | 1684.57 | 0.88 | 96.96 | 0.13 |
| 56-75-7 & 918504-65-1 | 1721.57 | 0.53 | 136.93 | 0.16 | 110.17 | 0.12 |
| 56-75-7 & 60-54-8 | 1721.57 | 0.53 | 2740.00 | 0.71 | 132.80 | 0.16 |
| 56-75-7 & 7385-67-3 | 1721.57 | 0.53 | 2360.40 | 0.79 | 183.03 | 0.15 |
| 56-75-7 & 85622-93-1 | 1721.57 | 0.53 | 213.13 | 0.71 | 88.82 | 0.19 |
| 56-75-7 & 71125-38-7 | 1721.57 | 0.53 | 533.93 | 0.38 | 96.02 | 0.23 |
| 56-75-7 & 1073154-85-4 | 1721.57 | 0.53 | 5538.33 | 0.92 | 240.53 | 0.16 |
| 56-75-7 & 50-78-2 | 1721.57 | 0.53 | 534.40 | 0.54 | 94.33 | 0.24 |
| 56-75-7 & 274693-27-5 | 1721.57 | 0.53 | 232.10 | 0.09 | 86.84 | 0.08 |
| 56-75-7 & 73963-72-1 | 1721.57 | 0.53 | 1367.00 | 0.63 | 92.40 | 0.13 |
| 56-75-7 & 63610-08-2 | 1721.57 | 0.53 | 19016.00 | 0.61 | 94.77 | 0.20 |
| 56-75-7 & 656247-17-5 | 1721.57 | 0.53 | 3728.00 | 0.34 | 220.37 | 0.16 |
| 57808-66-9 & 656247-17-5 | 351.90 | 0.51 | 3728.00 | 0.34 | 257.47 | 0.12 |
| 103577-45-3 & 3599-32-4 | 1192.67 | 0.08 | 2886.87 | 1.00 | 109.27 | 0.10 |
| 103577-45-3 & 19660-77-6 | 1192.67 | 0.08 | 456.47 | 0.61 | 297.37 | 0.08 |
| 103577-45-3 & 76824-35-6 | 1192.67 | 0.08 | 3162.00 | 0.63 | 169.37 | 0.11 |
| 51481-61-9 & 76824-35-6 | 1943.67 | 0.98 | 3162.00 | 0.63 | 143.80 | 0.17 |
| 51481-61-9 & 52549-17-4 | 1943.67 | 0.98 | 3274.33 | 0.65 | 125.90 | 0.02 |
| 51481-61-9 & 50-02-2 | 1943.67 | 0.98 | 1354.23 | 0.73 | 123.73 | 0.01 |
| 51481-61-9 & 86386-73-4 | 1943.67 | 0.98 | 716.57 | 0.55 | 126.50 | 0.04 |
| 50-78-2 & 63610-08-2 | 534.40 | 0.54 | 19016.00 | 0.61 | 124.77 | 0.02 |
| 1073154-85-4 & 274693-27-5 | 5538.33 | 0.92 | 232.10 | 0.09 | 138.80 | 0.06 |
| 1073154-85-4 & 73963-72-1 | 5538.33 | 0.92 | 1367.00 | 0.63 | 202.67 | 0.09 |
| 1073154-85-4 & 63610-08-2 | 5538.33 | 0.92 | 19016.00 | 0.61 | 306.30 | 0.16 |
| 50-02-2 & 50-78-2 | 1354.23 | 0.73 | 534.40 | 0.54 | 103.83 | 0.07 |
| 50-02-2 & 1195765-45-7 | 1354.23 | 0.73 | 953.87 | 0.64 | 248.37 | 0.07 |
| 50-02-2 & 85622-93-1 | 1354.23 | 0.73 | 213.13 | 0.71 | 128.27 | 0.03 |
| 50-02-2 & 19660-77-6 | 1354.23 | 0.73 | 456.47 | 0.61 | 195.60 | 0.03 |
| 50-02-2 & 76824-35-6 | 1354.23 | 0.73 | 3162.00 | 0.63 | 126.23 | 0.03 |
| 50-02-2 & 137234-62-9 | 1354.23 | 0.73 | 4169.33 | 1.00 | 99.98 | 0.07 |
| 50-02-2 & 86386-73-4 | 1354.23 | 0.73 | 716.57 | 0.55 | 105.73 | 0.07 |
| 50-02-2 & 95058-81-4 | 1354.23 | 0.73 | 1684.57 | 0.88 | 113.23 | 0.06 |
| 50-02-2 & 59-05-2 | 1354.23 | 0.73 | 1396.07 | 0.91 | 127.03 | 0.06 |
| 50-02-2 & 57-62-5 | 1354.23 | 0.73 | 1303.00 | 0.42 | 246.80 | 0.05 |
| 50-02-2 & 117976-89-3 | 1354.23 | 0.73 | 521.03 | 0.70 | 114.00 | 0.07 |
| 50-02-2 & 71125-38-7 | 1354.23 | 0.73 | 533.93 | 0.38 | 111.83 | 0.03 |
| 50-02-2 & 656247-17-5 | 1354.23 | 0.73 | 3728.00 | 0.34 | 220.40 | 0.14 |
| 50-02-2 & 7385-67-3 | 1354.23 | 0.73 | 2360.40 | 0.79 | 225.67 | 0.07 |
| 50-02-2 & 70458-96-7 | 1354.23 | 0.73 | 1719.00 | 0.84 | 176.17 | 0.06 |
| 50-02-2 & 52549-17-4 | 1354.23 | 0.73 | 3274.33 | 0.65 | 110.67 | 0.06 |
| 50-02-2 & 19685-09-7 | 1354.23 | 0.73 | 1606.00 | 0.23 | 142.40 | 0.02 |
| 50-02-2 & 871700-17-3 | 1354.23 | 0.73 | 140.57 | 0.21 | 119.80 | 0.03 |
| 50-02-2 & 60-54-8 | 1354.23 | 0.73 | 2740.00 | 0.71 | 144.13 | 0.07 |
| 50-02-2 & 274693-27-5 | 1354.23 | 0.73 | 232.10 | 0.09 | 95.20 | 0.13 |
| 50-02-2 & 918504-65-1 | 1354.23 | 0.73 | 136.93 | 0.16 | 117.80 | 0.02 |
| 50-02-2 & 73963-72-1 | 1354.23 | 0.73 | 1367.00 | 0.63 | 97.17 | 0.15 |
| 50-02-2 & 7689-03-4 | 1354.23 | 0.73 | 1990.33 | 0.53 | 121.20 | 0.05 |
| 50-02-2 & 63610-08-2 | 1354.23 | 0.73 | 19016.00 | 0.61 | 105.59 | 0.20 |
| 76824-35-6 & 380843-75-4 | 3162.00 | 0.63 | 552.10 | 0.45 | 232.83 | 0.22 |
| 76824-35-6 & 1195765-45-7 | 3162.00 | 0.63 | 953.87 | 0.64 | 216.87 | 0.01 |
| 76824-35-6 & 1073154-85-4 | 3162.00 | 0.63 | 5538.33 | 0.92 | 169.20 | 0.04 |
| 76824-35-6 & 85622-93-1 | 3162.00 | 0.63 | 213.13 | 0.71 | 123.77 | 0.03 |
| 76824-35-6 & 19660-77-6 | 3162.00 | 0.63 | 456.47 | 0.61 | 139.47 | 0.04 |
| 76824-35-6 & 86386-73-4 | 3162.00 | 0.63 | 716.57 | 0.55 | 122.93 | 0.06 |
| 76824-35-6 & 21967-41-9 | 3162.00 | 0.63 | 1035.33 | 0.36 | 120.40 | 0.05 |
| 76824-35-6 & 95058-81-4 | 3162.00 | 0.63 | 1684.57 | 0.88 | 125.50 | 0.01 |
| 76824-35-6 & 59-05-2 | 3162.00 | 0.63 | 1396.07 | 0.91 | 158.53 | 0.02 |
| 76824-35-6 & 57-62-5 | 3162.00 | 0.63 | 1303.00 | 0.42 | 215.67 | 0.04 |
| 76824-35-6 & 117976-89-3 | 3162.00 | 0.63 | 521.03 | 0.70 | 133.67 | 0.02 |
| 76824-35-6 & 71125-38-7 | 3162.00 | 0.63 | 533.93 | 0.38 | 103.60 | 0.06 |
| 76824-35-6 & 656247-17-5 | 3162.00 | 0.63 | 3728.00 | 0.34 | 263.00 | 0.18 |
| 76824-35-6 & 7385-67-3 | 3162.00 | 0.63 | 2360.40 | 0.79 | 140.03 | 0.03 |
| 76824-35-6 & 19685-09-7 | 3162.00 | 0.63 | 1606.00 | 0.23 | 123.63 | 0.06 |
| 76824-35-6 & 871700-17-3 | 3162.00 | 0.63 | 140.57 | 0.21 | 149.77 | 0.04 |
| 76824-35-6 & 60-54-8 | 3162.00 | 0.63 | 2740.00 | 0.71 | 127.00 | 0.05 |
| 76824-35-6 & 274693-27-5 | 3162.00 | 0.63 | 232.10 | 0.09 | 107.43 | 0.06 |
| 76824-35-6 & 918504-65-1 | 3162.00 | 0.63 | 136.93 | 0.16 | 118.67 | 0.06 |
| 76824-35-6 & 73963-72-1 | 3162.00 | 0.63 | 1367.00 | 0.63 | 115.37 | 0.07 |
| 76824-35-6 & 7689-03-4 | 3162.00 | 0.63 | 1990.33 | 0.53 | 104.27 | 0.03 |
| 76824-35-6 & 63610-08-2 | 3162.00 | 0.63 | 19016.00 | 0.61 | 107.17 | 0.07 |
| 86386-73-4 & 380843-75-4 | 716.57 | 0.55 | 552.10 | 0.45 | 252.33 | 0.14 |
| 86386-73-4 & 1195765-45-7 | 716.57 | 0.55 | 953.87 | 0.64 | 224.10 | 0.02 |
| 86386-73-4 & 85622-93-1 | 716.57 | 0.55 | 213.13 | 0.71 | 99.07 | 0.07 |
| 86386-73-4 & 137234-62-9 | 716.57 | 0.55 | 4169.33 | 1.00 | 96.99 | 0.09 |
| 86386-73-4 & 21967-41-9 | 716.57 | 0.55 | 1035.33 | 0.36 | 118.10 | 0.12 |
| 86386-73-4 & 95058-81-4 | 716.57 | 0.55 | 1684.57 | 0.88 | 104.70 | 0.11 |
| 86386-73-4 & 57-62-5 | 716.57 | 0.55 | 1303.00 | 0.42 | 180.07 | 0.05 |
| 86386-73-4 & 117976-89-3 | 716.57 | 0.55 | 521.03 | 0.70 | 107.10 | 0.08 |
| 86386-73-4 & 71125-38-7 | 716.57 | 0.55 | 533.93 | 0.38 | 103.47 | 0.06 |
| 86386-73-4 & 7385-67-3 | 716.57 | 0.55 | 2360.40 | 0.79 | 162.90 | 0.06 |
| 86386-73-4 & 70458-96-7 | 716.57 | 0.55 | 1719.00 | 0.84 | 119.77 | 0.09 |
| 86386-73-4 & 19685-09-7 | 716.57 | 0.55 | 1606.00 | 0.23 | 106.57 | 0.10 |
| 86386-73-4 & 871700-17-3 | 716.57 | 0.55 | 140.57 | 0.21 | 111.13 | 0.11 |
| 86386-73-4 & 60-54-8 | 716.57 | 0.55 | 2740.00 | 0.71 | 119.27 | 0.07 |
| 86386-73-4 & 918504-65-1 | 716.57 | 0.55 | 136.93 | 0.16 | 112.97 | 0.11 |
| 86386-73-4 & 7689-03-4 | 716.57 | 0.55 | 1990.33 | 0.53 | 107.97 | 0.11 |
| 52549-17-4 & 50-78-2 | 3274.33 | 0.65 | 534.40 | 0.54 | 101.33 | 0.05 |
| 52549-17-4 & 380843-75-4 | 3274.33 | 0.65 | 552.10 | 0.45 | 283.47 | 0.10 |
| 52549-17-4 & 1195765-45-7 | 3274.33 | 0.65 | 953.87 | 0.64 | 218.47 | 0.01 |
| 52549-17-4 & 85622-93-1 | 3274.33 | 0.65 | 213.13 | 0.71 | 116.97 | 0.03 |
| 52549-17-4 & 19660-77-6 | 3274.33 | 0.65 | 456.47 | 0.61 | 152.73 | 0.02 |
| 52549-17-4 & 76824-35-6 | 3274.33 | 0.65 | 3162.00 | 0.63 | 101.63 | 0.16 |
| 52549-17-4 & 137234-62-9 | 3274.33 | 0.65 | 4169.33 | 1.00 | 111.03 | 0.09 |
| 52549-17-4 & 21967-41-9 | 3274.33 | 0.65 | 1035.33 | 0.36 | 98.78 | 0.04 |
| 52549-17-4 & 95058-81-4 | 3274.33 | 0.65 | 1684.57 | 0.88 | 109.63 | 0.02 |
| 52549-17-4 & 59-05-2 | 3274.33 | 0.65 | 1396.07 | 0.91 | 129.97 | 0.05 |
| 52549-17-4 & 57-62-5 | 3274.33 | 0.65 | 1303.00 | 0.42 | 184.43 | 0.17 |
| 52549-17-4 & 117976-89-3 | 3274.33 | 0.65 | 521.03 | 0.70 | 123.07 | 0.04 |
| 52549-17-4 & 71125-38-7 | 3274.33 | 0.65 | 533.93 | 0.38 | 105.83 | 0.03 |
| 52549-17-4 & 656247-17-5 | 3274.33 | 0.65 | 3728.00 | 0.34 | 261.13 | 0.13 |
| 52549-17-4 & 7385-67-3 | 3274.33 | 0.65 | 2360.40 | 0.79 | 170.83 | 0.04 |
| 52549-17-4 & 70458-96-7 | 3274.33 | 0.65 | 1719.00 | 0.84 | 139.73 | 0.19 |
| 52549-17-4 & 871700-17-3 | 3274.33 | 0.65 | 140.57 | 0.21 | 119.53 | 0.03 |
| 52549-17-4 & 557795-19-4 | 3274.33 | 0.65 | 3914.33 | 0.32 | 280.00 | 0.11 |
| 52549-17-4 & 60-54-8 | 3274.33 | 0.65 | 2740.00 | 0.71 | 120.10 | 0.05 |
| 52549-17-4 & 274693-27-5 | 3274.33 | 0.65 | 232.10 | 0.09 | 107.70 | 0.05 |
| 52549-17-4 & 918504-65-1 | 3274.33 | 0.65 | 136.93 | 0.16 | 108.17 | 0.08 |
| 52549-17-4 & 73963-72-1 | 3274.33 | 0.65 | 1367.00 | 0.63 | 115.50 | 0.06 |
| 52549-17-4 & 63610-08-2 | 3274.33 | 0.65 | 19016.00 | 0.61 | 108.33 | 0.04 |
| 557795-19-4 & 656247-17-5 | 3914.33 | 0.32 | 3728.00 | 0.34 | 228.87 | 0.16 |
| 557795-19-4 & 274693-27-5 | 3914.33 | 0.32 | 232.10 | 0.09 | 240.23 | 0.11 |
| 51481-61-9 & 50-78-2 | 1943.67 | 0.98 | 534.40 | 0.54 | 119.70 | 0.05 |
| 51481-61-9 & 1195765-45-7 | 1943.67 | 0.98 | 953.87 | 0.64 | 182.60 | 0.03 |
| 51481-61-9 & 1073154-85-4 | 1943.67 | 0.98 | 5538.33 | 0.92 | 247.37 | 0.04 |
| 51481-61-9 & 85622-93-1 | 1943.67 | 0.98 | 213.13 | 0.71 | 104.77 | 0.11 |
| 51481-61-9 & 19660-77-6 | 1943.67 | 0.98 | 456.47 | 0.61 | 164.40 | 0.04 |
| 51481-61-9 & 137234-62-9 | 1943.67 | 0.98 | 4169.33 | 1.00 | 108.10 | 0.06 |
| 51481-61-9 & 21967-41-9 | 1943.67 | 0.98 | 1035.33 | 0.36 | 117.40 | 0.03 |
| 51481-61-9 & 95058-81-4 | 1943.67 | 0.98 | 1684.57 | 0.88 | 105.07 | 0.06 |
| 51481-61-9 & 59-05-2 | 1943.67 | 0.98 | 1396.07 | 0.91 | 130.17 | 0.04 |
| 51481-61-9 & 57-62-5 | 1943.67 | 0.98 | 1303.00 | 0.42 | 188.43 | 0.02 |
| 51481-61-9 & 117976-89-3 | 1943.67 | 0.98 | 521.03 | 0.70 | 107.20 | 0.03 |
| 51481-61-9 & 71125-38-7 | 1943.67 | 0.98 | 533.93 | 0.38 | 98.09 | 0.06 |
| 51481-61-9 & 656247-17-5 | 1943.67 | 0.98 | 3728.00 | 0.34 | 258.40 | 0.12 |
| 51481-61-9 & 70458-96-7 | 1943.67 | 0.98 | 1719.00 | 0.84 | 144.90 | 0.03 |
| 51481-61-9 & 871700-17-3 | 1943.67 | 0.98 | 140.57 | 0.21 | 125.70 | 0.08 |
| 51481-61-9 & 60-54-8 | 1943.67 | 0.98 | 2740.00 | 0.71 | 142.17 | 0.06 |
| 51481-61-9 & 274693-27-5 | 1943.67 | 0.98 | 232.10 | 0.09 | 117.10 | 0.07 |
| 51481-61-9 & 918504-65-1 | 1943.67 | 0.98 | 136.93 | 0.16 | 116.60 | 0.09 |
| 51481-61-9 & 73963-72-1 | 1943.67 | 0.98 | 1367.00 | 0.63 | 104.57 | 0.10 |
| 51481-61-9 & 63610-08-2 | 1943.67 | 0.98 | 19016.00 | 0.61 | 111.17 | 0.05 |
| 23214-92-8 & 1195765-45-7 | 4562.00 | 0.39 | 953.87 | 0.64 | 220.87 | 0.05 |
| 1195765-45-7 & 50-78-2 | 953.87 | 0.64 | 534.40 | 0.54 | 275.83 | 0.02 |
| 1195765-45-7 & 1073154-85-4 | 953.87 | 0.64 | 5538.33 | 0.92 | 310.00 | 0.02 |
| 1195765-45-7 & 137234-62-9 | 953.87 | 0.64 | 4169.33 | 1.00 | 259.47 | 0.03 |
| 1195765-45-7 & 21967-41-9 | 953.87 | 0.64 | 1035.33 | 0.36 | 230.40 | 0.03 |
| 1195765-45-7 & 95058-81-4 | 953.87 | 0.64 | 1684.57 | 0.88 | 261.67 | 0.02 |
| 1195765-45-7 & 71125-38-7 | 953.87 | 0.64 | 533.93 | 0.38 | 223.57 | 0.03 |
| 1195765-45-7 & 7385-67-3 | 953.87 | 0.64 | 2360.40 | 0.79 | 303.80 | 0.03 |
| 1195765-45-7 & 871700-17-3 | 953.87 | 0.64 | 140.57 | 0.21 | 195.60 | 0.04 |
| 1195765-45-7 & 60-54-8 | 953.87 | 0.64 | 2740.00 | 0.71 | 232.90 | 0.02 |
| 1195765-45-7 & 918504-65-1 | 953.87 | 0.64 | 136.93 | 0.16 | 240.17 | 0.04 |
| 1195765-45-7 & 73963-72-1 | 953.87 | 0.64 | 1367.00 | 0.63 | 211.20 | 0.04 |
| 1195765-45-7 & 63610-08-2 | 953.87 | 0.64 | 19016.00 | 0.61 | 234.90 | 0.03 |
| 137234-62-9 & 19660-77-6 | 4169.33 | 1.00 | 456.47 | 0.61 | 220.27 | 0.05 |
| 137234-62-9 & 50-78-2 | 4169.33 | 1.00 | 534.40 | 0.54 | 111.20 | 0.12 |
| 137234-62-9 & 380843-75-4 | 4169.33 | 1.00 | 552.10 | 0.45 | 350.57 | 0.24 |
| 137234-62-9 & 1073154-85-4 | 4169.33 | 1.00 | 5538.33 | 0.92 | 202.67 | 0.03 |
| 137234-62-9 & 85622-93-1 | 4169.33 | 1.00 | 213.13 | 0.71 | 113.73 | 0.04 |
| 137234-62-9 & 21967-41-9 | 4169.33 | 1.00 | 1035.33 | 0.36 | 113.33 | 0.04 |
| 137234-62-9 & 59-05-2 | 4169.33 | 1.00 | 1396.07 | 0.91 | 153.03 | 0.02 |
| 137234-62-9 & 71125-38-7 | 4169.33 | 1.00 | 533.93 | 0.38 | 108.90 | 0.05 |
| 137234-62-9 & 656247-17-5 | 4169.33 | 1.00 | 3728.00 | 0.34 | 160.80 | 0.10 |
| 137234-62-9 & 7385-67-3 | 4169.33 | 1.00 | 2360.40 | 0.79 | 170.73 | 0.03 |
| 137234-62-9 & 19685-09-7 | 4169.33 | 1.00 | 1606.00 | 0.23 | 117.83 | 0.04 |
| 137234-62-9 & 60-54-8 | 4169.33 | 1.00 | 2740.00 | 0.71 | 126.37 | 0.08 |
| 137234-62-9 & 274693-27-5 | 4169.33 | 1.00 | 232.10 | 0.09 | 105.40 | 0.08 |
| 137234-62-9 & 73963-72-1 | 4169.33 | 1.00 | 1367.00 | 0.63 | 116.33 | 0.02 |
| 137234-62-9 & 63610-08-2 | 4169.33 | 1.00 | 19016.00 | 0.61 | 106.03 | 0.10 |
| 86386-73-4 & 50-78-2 | 716.57 | 0.55 | 534.40 | 0.54 | 125.80 | 0.02 |
| 86386-73-4 & 1073154-85-4 | 716.57 | 0.55 | 5538.33 | 0.92 | 213.37 | 0.02 |
| 86386-73-4 & 274693-27-5 | 716.57 | 0.55 | 232.10 | 0.09 | 112.47 | 0.01 |
| 86386-73-4 & 73963-72-1 | 716.57 | 0.55 | 1367.00 | 0.63 | 129.73 | 0.01 |
| 21967-41-9 & 50-78-2 | 1035.33 | 0.36 | 534.40 | 0.54 | 100.69 | 0.18 |
| 21967-41-9 & 1073154-85-4 | 1035.33 | 0.36 | 5538.33 | 0.92 | 125.77 | 0.07 |
| 21967-41-9 & 85622-93-1 | 1035.33 | 0.36 | 213.13 | 0.71 | 95.33 | 0.11 |
| 21967-41-9 & 95058-81-4 | 1035.33 | 0.36 | 1684.57 | 0.88 | 86.80 | 0.17 |
| 21967-41-9 & 59-05-2 | 1035.33 | 0.36 | 1396.07 | 0.91 | 170.07 | 0.06 |
| 21967-41-9 & 71125-38-7 | 1035.33 | 0.36 | 533.93 | 0.38 | 101.40 | 0.12 |
| 21967-41-9 & 656247-17-5 | 1035.33 | 0.36 | 3728.00 | 0.34 | 193.80 | 0.12 |
| 21967-41-9 & 7385-67-3 | 1035.33 | 0.36 | 2360.40 | 0.79 | 108.37 | 0.03 |
| 21967-41-9 & 871700-17-3 | 1035.33 | 0.36 | 140.57 | 0.21 | 143.87 | 0.22 |
| 21967-41-9 & 557795-19-4 | 1035.33 | 0.36 | 3914.33 | 0.32 | 297.97 | 0.14 |
| 21967-41-9 & 274693-27-5 | 1035.33 | 0.36 | 232.10 | 0.09 | 104.87 | 0.19 |
| 21967-41-9 & 918504-65-1 | 1035.33 | 0.36 | 136.93 | 0.16 | 110.87 | 0.17 |
| 21967-41-9 & 73963-72-1 | 1035.33 | 0.36 | 1367.00 | 0.63 | 108.70 | 0.16 |
| 21967-41-9 & 63610-08-2 | 1035.33 | 0.36 | 19016.00 | 0.61 | 110.07 | 0.20 |
| 21967-41-9 & 33069-62-4 | 1035.33 | 0.36 | 6681.00 | 0.70 | 115.40 | 0.22 |
| 59-05-2 & 71125-38-7 | 1396.07 | 0.91 | 533.93 | 0.38 | 108.53 | 0.09 |
| 59-05-2 & 7385-67-3 | 1396.07 | 0.91 | 2360.40 | 0.79 | 158.13 | 0.02 |
| 59-05-2 & 19685-09-7 | 1396.07 | 0.91 | 1606.00 | 0.23 | 119.00 | 0.04 |
| 57-62-5 & 50-78-2 | 1303.00 | 0.42 | 534.40 | 0.54 | 245.47 | 0.03 |
| 57-62-5 & 85622-93-1 | 1303.00 | 0.42 | 213.13 | 0.71 | 278.40 | 0.02 |
| 57-62-5 & 137234-62-9 | 1303.00 | 0.42 | 4169.33 | 1.00 | 246.80 | 0.04 |
| 57-62-5 & 21967-41-9 | 1303.00 | 0.42 | 1035.33 | 0.36 | 274.37 | 0.05 |
| 57-62-5 & 95058-81-4 | 1303.00 | 0.42 | 1684.57 | 0.88 | 222.27 | 0.02 |
| 57-62-5 & 59-05-2 | 1303.00 | 0.42 | 1396.07 | 0.91 | 314.13 | 0.05 |
| 57-62-5 & 117976-89-3 | 1303.00 | 0.42 | 521.03 | 0.70 | 241.03 | 0.03 |
| 57-62-5 & 71125-38-7 | 1303.00 | 0.42 | 533.93 | 0.38 | 255.67 | 0.02 |
| 57-62-5 & 656247-17-5 | 1303.00 | 0.42 | 3728.00 | 0.34 | 250.80 | 0.16 |
| 57-62-5 & 7385-67-3 | 1303.00 | 0.42 | 2360.40 | 0.79 | 249.77 | 0.03 |
| 57-62-5 & 19685-09-7 | 1303.00 | 0.42 | 1606.00 | 0.23 | 253.53 | 0.03 |
| 57-62-5 & 871700-17-3 | 1303.00 | 0.42 | 140.57 | 0.21 | 218.70 | 0.02 |
| 57-62-5 & 60-54-8 | 1303.00 | 0.42 | 2740.00 | 0.71 | 225.23 | 0.01 |
| 57-62-5 & 274693-27-5 | 1303.00 | 0.42 | 232.10 | 0.09 | 293.83 | 0.01 |
| 57-62-5 & 918504-65-1 | 1303.00 | 0.42 | 136.93 | 0.16 | 174.03 | 0.02 |
| 57-62-5 & 73963-72-1 | 1303.00 | 0.42 | 1367.00 | 0.63 | 256.53 | 0.03 |
| 57-62-5 & 7689-03-4 | 1303.00 | 0.42 | 1990.33 | 0.53 | 246.63 | 0.01 |
| 57-62-5 & 3599-32-4 | 1303.00 | 0.42 | 2886.87 | 1.00 | 248.87 | 0.04 |
| 57-62-5 & 33069-62-4 | 1303.00 | 0.42 | 6681.00 | 0.70 | 349.97 | 0.03 |
| 934660-93-2 & 656247-17-5 | 2026.60 | 0.51 | 3728.00 | 0.34 | 169.60 | 0.17 |
| 81103-11-9 & 380843-75-4 | 1851.70 | 0.67 | 552.10 | 0.45 | 338.67 | 0.14 |
| 117976-89-3 & 50-78-2 | 521.03 | 0.70 | 534.40 | 0.54 | 128.00 | 0.04 |
| 117976-89-3 & 85622-93-1 | 521.03 | 0.70 | 213.13 | 0.71 | 159.90 | 0.27 |
| 117976-89-3 & 137234-62-9 | 521.03 | 0.70 | 4169.33 | 1.00 | 130.03 | 0.10 |
| 117976-89-3 & 21967-41-9 | 521.03 | 0.70 | 1035.33 | 0.36 | 95.93 | 0.14 |
| 117976-89-3 & 71125-38-7 | 521.03 | 0.70 | 533.93 | 0.38 | 183.00 | 0.29 |
| 117976-89-3 & 7385-67-3 | 521.03 | 0.70 | 2360.40 | 0.79 | 298.93 | 0.22 |
| 117976-89-3 & 871700-17-3 | 521.03 | 0.70 | 140.57 | 0.21 | 157.97 | 0.19 |
| 117976-89-3 & 60-54-8 | 521.03 | 0.70 | 2740.00 | 0.71 | 153.73 | 0.21 |
| 117976-89-3 & 274693-27-5 | 521.03 | 0.70 | 232.10 | 0.09 | 114.00 | 0.02 |
| 117976-89-3 & 918504-65-1 | 521.03 | 0.70 | 136.93 | 0.16 | 138.33 | 0.17 |
| 117976-89-3 & 73963-72-1 | 521.03 | 0.70 | 1367.00 | 0.63 | 116.87 | 0.03 |
| 117976-89-3 & 63610-08-2 | 521.03 | 0.70 | 19016.00 | 0.61 | 112.97 | 0.02 |
| 70458-96-7 & 50-78-2 | 1719.00 | 0.84 | 534.40 | 0.54 | 271.83 | 0.32 |
| 70458-96-7 & 85622-93-1 | 1719.00 | 0.84 | 213.13 | 0.71 | 187.93 | 0.13 |
| 70458-96-7 & 137234-62-9 | 1719.00 | 0.84 | 4169.33 | 1.00 | 170.27 | 0.03 |
| 70458-96-7 & 95058-81-4 | 1719.00 | 0.84 | 1684.57 | 0.88 | 164.87 | 0.22 |
| 70458-96-7 & 59-05-2 | 1719.00 | 0.84 | 1396.07 | 0.91 | 228.17 | 0.03 |
| 70458-96-7 & 117976-89-3 | 1719.00 | 0.84 | 521.03 | 0.70 | 199.47 | 0.03 |
| 70458-96-7 & 71125-38-7 | 1719.00 | 0.84 | 533.93 | 0.38 | 221.83 | 0.14 |
| 70458-96-7 & 656247-17-5 | 1719.00 | 0.84 | 3728.00 | 0.34 | 216.07 | 0.17 |
| 70458-96-7 & 871700-17-3 | 1719.00 | 0.84 | 140.57 | 0.21 | 163.33 | 0.01 |
| 70458-96-7 & 60-54-8 | 1719.00 | 0.84 | 2740.00 | 0.71 | 203.50 | 0.15 |
| 70458-96-7 & 274693-27-5 | 1719.00 | 0.84 | 232.10 | 0.09 | 321.20 | 0.13 |
| 70458-96-7 & 918504-65-1 | 1719.00 | 0.84 | 136.93 | 0.16 | 194.53 | 0.06 |
| 70458-96-7 & 73963-72-1 | 1719.00 | 0.84 | 1367.00 | 0.63 | 257.77 | 0.06 |
| 70458-96-7 & 63610-08-2 | 1719.00 | 0.84 | 19016.00 | 0.61 | 229.83 | 0.15 |
| 70458-96-7 & 3599-32-4 | 1719.00 | 0.84 | 2886.87 | 1.00 | 190.43 | 0.27 |
| 3599-32-4 & 14459-29-1 | 2886.87 | 1.00 | 5000.67 | 0.30 | 137.17 | 0.19 |
| 3599-32-4 & 380843-75-4 | 2886.87 | 1.00 | 552.10 | 0.45 | 184.00 | 0.07 |
| 3599-32-4 & 1073154-85-4 | 2886.87 | 1.00 | 5538.33 | 0.92 | 185.80 | 0.26 |
| 3599-32-4 & 113665-84-2 | 2886.87 | 1.00 | 460.10 | 0.32 | 165.73 | 0.27 |
| 3599-32-4 & 71125-38-7 | 2886.87 | 1.00 | 533.93 | 0.38 | 160.80 | 0.15 |
| 3599-32-4 & 58-32-2 | 2886.87 | 1.00 | 2549.00 | 0.95 | 156.70 | 0.22 |
| 3599-32-4 & 379231-04-6 | 2886.87 | 1.00 | 1374.87 | 0.76 | 166.93 | 0.09 |
| 3599-32-4 & 557795-19-4 | 2886.87 | 1.00 | 3914.33 | 0.32 | 174.10 | 0.27 |
| 3599-32-4 & 65277-42-1 | 2886.87 | 1.00 | 2835.00 | 1.00 | 194.20 | 0.29 |
| 3599-32-4 & 73963-72-1 | 2886.87 | 1.00 | 1367.00 | 0.63 | 167.57 | 0.27 |
| 426-13-1 & 1195765-45-7 | 70109.00 | 0.69 | 953.87 | 0.64 | 224.00 | 0.02 |
| 57-62-5 & 70458-96-7 | 1303.00 | 0.42 | 1719.00 | 0.84 | 223.43 | 0.02 |
| 59-05-2 & 50-78-2 | 1396.07 | 0.91 | 534.40 | 0.54 | 178.27 | 0.07 |
| 59-05-2 & 274693-27-5 | 1396.07 | 0.91 | 232.10 | 0.09 | 135.23 | 0.05 |
| 59-05-2 & 73963-72-1 | 1396.07 | 0.91 | 1367.00 | 0.63 | 212.40 | 0.10 |
| 59-05-2 & 63610-08-2 | 1396.07 | 0.91 | 19016.00 | 0.61 | 164.80 | 0.05 |
| 59-05-2 & 656247-17-5 | 1396.07 | 0.91 | 3728.00 | 0.34 | 250.03 | 0.13 |
| 871700-17-3 & 60-54-8 | 140.57 | 0.21 | 2740.00 | 0.71 | 134.17 | 0.13 |
| 871700-17-3 & 71125-38-7 | 140.57 | 0.21 | 533.93 | 0.38 | 112.23 | 0.11 |
| 871700-17-3 & 1073154-85-4 | 140.57 | 0.21 | 5538.33 | 0.92 | 208.77 | 0.06 |
| 871700-17-3 & 50-78-2 | 140.57 | 0.21 | 534.40 | 0.54 | 124.70 | 0.10 |
| 871700-17-3 & 274693-27-5 | 140.57 | 0.21 | 232.10 | 0.09 | 115.20 | 0.11 |
| 871700-17-3 & 656247-17-5 | 140.57 | 0.21 | 3728.00 | 0.34 | 228.87 | 0.16 |
| 19660-77-6 & 95058-81-4 | 456.47 | 0.61 | 1684.57 | 0.88 | 254.10 | 0.04 |
| 19660-77-6 & 7689-03-4 | 456.47 | 0.61 | 1990.33 | 0.53 | 231.80 | 0.03 |
| 19660-77-6 & 7385-67-3 | 456.47 | 0.61 | 2360.40 | 0.79 | 233.67 | 0.05 |
| 19660-77-6 & 71125-38-7 | 456.47 | 0.61 | 533.93 | 0.38 | 242.33 | 0.02 |
| 19660-77-6 & 50-78-2 | 456.47 | 0.61 | 534.40 | 0.54 | 326.77 | 0.11 |
| 19660-77-6 & 113665-84-2 | 456.47 | 0.61 | 460.10 | 0.32 | 248.63 | 0.03 |
| 19660-77-6 & 150322-43-3 | 456.47 | 0.61 | 839.80 | 0.43 | 278.60 | 0.09 |
| 19660-77-6 & 274693-27-5 | 456.47 | 0.61 | 232.10 | 0.09 | 284.77 | 0.03 |
| 19660-77-6 & 73963-72-1 | 456.47 | 0.61 | 1367.00 | 0.63 | 234.60 | 0.06 |
| 19660-77-6 & 63610-08-2 | 456.47 | 0.61 | 19016.00 | 0.61 | 244.30 | 0.04 |
| 33069-62-4 & 7681-93-8 | 6681.00 | 0.70 | 5014.67 | 0.26 | 146.03 | 0.10 |

**Table S2. List of 63 Commonly Used Small Molecule Drugs.** This table lists the drug combinations that successfully formed stable nanoparticles (defined as particle sizes smaller than 350 nm and PDI smaller than 0.3) when co-assembled with LA. The table includes CAS numbers, the hydrated particle size (nm) and PDI of each drug alone (A-size, A-PDI, B-size, B-PDI), and the final size and PDI of the assembled ternary nanodrugs.

| **Drug1** | **Drug2** | **Yes/No** | **Drug1** | **Drug2** | **Yes/No** |
| --- | --- | --- | --- | --- | --- |
| 33069-62-4 | 1195765-45-7 | no | 426-13-1 | [183321-74-6](https://china.guidechem.com/1554622/) | no |
| 33069-62-4 | 59-05-2 | no | 426-13-1 | 184475-35-2 | no |
| 33069-62-4 | [95058-81-4](https://www.guidechem.com/encyclopedia/gemcitabine-dic28627.html) | no | 426-13-1 | [934660-93-2](https://china.guidechem.com/1556922/detail.html) | no |
| 33069-62-4 | 85622-93-1 | no | 426-13-1 | 379231-04-6 | no |
| 33069-62-4 | [918504-65-1](https://www.guidechem.com/encyclopedia/vemurafenib-dic1173516.html) | no | 426-13-1 | 557795-19-4 | no |
| 33069-62-4 | 934660-93-2 | no | 426-13-1 | 1110813-31-4 | no |
| 33069-62-4 | 137234-62-9 | no | 426-13-1 | 380843-75-4 | no |
| 33069-62-4 | 871700-17-3 | no | 426-13-1 | 318-98-9 | no |
| 19660-77-6 | [183321-74-6](https://www.guidechem.com/encyclopedia/icotinib-dic1554622.html) | no | 426-13-1 | 113665-84-2 | no |
| 3599-32-4 | 23214-92-8 | no | 426-13-1 | [58-32-2](https://china.guidechem.com/217/) | no |
| 76824-35-6 | 57808-66-9 | no | 426-13-1 | 103577-45-3 | no |
| 73590-58-6 | 57808-66-9 | no | 426-13-1 | [33069-62-4](https://china.guidechem.com/22415/) | no |
| 73590-58-6 | 23214-92-8 | no | 59277-89-3 | 26787-78-0 | no |
| [934660-93-2](https://china.guidechem.com/1556922/detail.html) | [183321-74-6](https://china.guidechem.com/1554622/) | no | 59277-89-3 | 81103-11-9 | no |
| [934660-93-2](https://china.guidechem.com/1556922/detail.html) | [918504-65-1](https://china.guidechem.com/1173516/) | no | 59277-89-3 | 3599-32-4 | no |
| [934660-93-2](https://china.guidechem.com/1556922/detail.html) | 1195765-45-7 | no | 59277-89-3 | [934660-93-2](https://china.guidechem.com/1556922/detail.html) | no |
| [934660-93-2](https://china.guidechem.com/1556922/detail.html) | 137234-62-9 | no | 59277-89-3 | [183321-74-6](https://china.guidechem.com/1554622/) | no |
| [934660-93-2](https://china.guidechem.com/1556922/detail.html) | 59-05-2 | no | 59277-89-3 | 184475-35-2 | no |
| [934660-93-2](https://china.guidechem.com/1556922/detail.html) | [7681-93-8](https://china.guidechem.com/15390/) | no | 59277-89-3 | 379231-04-6 | no |
| [934660-93-2](https://china.guidechem.com/1556922/detail.html) | 19660-77-6 | no | 59277-89-3 | 1110813-31-4 | no |
| 21967-41-9 | [65277-42-1](https://china.guidechem.com/26080/) | no | 59277-89-3 | 557795-19-4 | no |
| 184475-35-2 | [934660-93-2](https://china.guidechem.com/1556922/detail.html) | no | 59277-89-3 | 380843-75-4 | no |
| 184475-35-2 | [183321-74-6](https://china.guidechem.com/1554622/) | no | 59277-89-3 | 23214-92-8 | no |
| 184475-35-2 | [7681-93-8](https://china.guidechem.com/15390/) | no | 59277-89-3 | [14459-29-1](https://china.guidechem.com/114399/detail.html) | no |
| 184475-35-2 | [95058-81-4](https://china.guidechem.com/28627/detail.html) | no | 114-07-8 | 56-75-7 | no |
| 184475-35-2 | 59-05-2 | no | 114-07-8 | 67-45-8 | no |
| 184475-35-2 | 137234-62-9 | no | 114-07-8 | [183321-74-6](https://china.guidechem.com/1554622/) | no |
| 871700-17-3 | [7681-93-8](https://china.guidechem.com/15390/) | no | 114-07-8 | [934660-93-2](https://china.guidechem.com/1556922/detail.html) | no |
| 871700-17-3 | [934660-93-2](https://china.guidechem.com/1556922/detail.html) | no | 114-07-8 | 184475-35-2 | no |
| 871700-17-3 | 184475-35-2 | no | 114-07-8 | 871700-17-3 | no |
| 871700-17-3 | [183321-74-6](https://china.guidechem.com/1554622/) | no | 114-07-8 | 3599-32-4 | no |
| 871700-17-3 | [95058-81-4](https://china.guidechem.com/28627/detail.html) | no | 114-07-8 | [14459-29-1](https://china.guidechem.com/114399/detail.html) | no |
| [183321-74-6](https://china.guidechem.com/1554622/) | [7681-93-8](https://china.guidechem.com/15390/) | no | 114-07-8 | 23214-92-8 | no |
| [183321-74-6](https://china.guidechem.com/1554622/) | 59-05-2 | no | 114-07-8 | 443-48-1 | no |
| [183321-74-6](https://china.guidechem.com/1554622/) | [918504-65-1](https://china.guidechem.com/1173516/) | no | 114-07-8 | 100986-85-4 | no |
| [95058-81-4](https://china.guidechem.com/28627/detail.html) | 137234-62-9 | no | 114-07-8 | 57808-66-9 | no |
| [95058-81-4](https://china.guidechem.com/28627/detail.html) | [183321-74-6](https://china.guidechem.com/1554622/) | no | 114-07-8 | 103577-45-3 | no |
| [95058-81-4](https://china.guidechem.com/28627/detail.html) | [934660-93-2](https://china.guidechem.com/1556922/detail.html) | no | 114-07-8 | 51481-61-9 | no |
| [95058-81-4](https://china.guidechem.com/28627/detail.html) | [918504-65-1](https://china.guidechem.com/1173516/) | no | 114-07-8 | 50-02-2 | no |
| 3599-32-4 | [33069-62-4](https://china.guidechem.com/22415/) | no | 114-07-8 | 32986-56-4 | no |
| 3599-32-4 | 19685-09-7 | no | 114-07-8 | 76824-35-6 | no |
| [14459-29-1](https://china.guidechem.com/114399/detail.html) | [33069-62-4](https://china.guidechem.com/22415/) | no | 114-07-8 | 81103-11-9 | no |
| [14459-29-1](https://china.guidechem.com/114399/detail.html) | 59-05-2 | no | 114-07-8 | 57-62-5 | no |
| [33069-62-4](https://china.guidechem.com/22415/) | 19685-09-7 | no | 114-07-8 | 117976-89-3 | no |
| [33069-62-4](https://china.guidechem.com/22415/) | 184475-35-2 | no | 114-07-8 | 1195765-45-7 | no |
| [33069-62-4](https://china.guidechem.com/22415/) | [183321-74-6](https://china.guidechem.com/1554622/) | no | 114-07-8 | 137234-62-9 | no |
| [33069-62-4](https://china.guidechem.com/22415/) | [7689-03-4](https://china.guidechem.com/15395/detail.html) | no | 114-07-8 | [65277-42-1](https://china.guidechem.com/26080/) | no |
| [7689-03-4](https://china.guidechem.com/15395/detail.html) | [183321-74-6](https://china.guidechem.com/1554622/) | no | 114-07-8 | 59-05-2 | no |
| [7689-03-4](https://china.guidechem.com/15395/detail.html) | 184475-35-2 | no | 114-07-8 | [95058-81-4](https://china.guidechem.com/28627/detail.html) | no |
| [7689-03-4](https://china.guidechem.com/15395/detail.html) | 871700-17-3 | no | 114-07-8 | [918504-65-1](https://china.guidechem.com/1173516/) | no |
| [7689-03-4](https://china.guidechem.com/15395/detail.html) | 137234-62-9 | no | 114-07-8 | [60-54-8](https://china.guidechem.com/279/) | no |
| [7689-03-4](https://china.guidechem.com/15395/detail.html) | [7681-93-8](https://china.guidechem.com/15390/) | no | 114-07-8 | 19685-09-7 | no |
| [7689-03-4](https://china.guidechem.com/15395/detail.html) | [934660-93-2](https://china.guidechem.com/1556922/detail.html) | no | 114-07-8 | 379231-04-6 | no |
| [7689-03-4](https://china.guidechem.com/15395/detail.html) | [918504-65-1](https://china.guidechem.com/1173516/) | no | 114-07-8 | 380843-75-4 | no |
| [7689-03-4](https://china.guidechem.com/15395/detail.html) | 85622-93-1 | no | 114-07-8 | 1110813-31-4 | no |
| [7689-03-4](https://china.guidechem.com/15395/detail.html) | [95058-81-4](https://china.guidechem.com/28627/detail.html) | no | 114-07-8 | 1073154-85-4 | no |
| [7689-03-4](https://china.guidechem.com/15395/detail.html) | 59-05-2 | no | 114-07-8 | 318-98-9 | no |
| [7689-03-4](https://china.guidechem.com/15395/detail.html) | 19685-09-7 | no | 114-07-8 | 113665-84-2 | no |
| [7689-03-4](https://china.guidechem.com/15395/detail.html) | 1195765-45-7 | no | 114-07-8 | 274693-27-5 | no |
| 23214-92-8 | 184475-35-2 | no | 114-07-8 | 557795-19-4 | no |
| 23214-92-8 | 871700-17-3 | no | 114-07-8 | 63610-08-2 | no |
| 23214-92-8 | 19685-09-7 | no | 114-07-8 | [58-32-2](https://china.guidechem.com/217/) | no |
| 23214-92-8 | [183321-74-6](https://china.guidechem.com/1554622/) | no | 114-07-8 | [33069-62-4](https://china.guidechem.com/22415/) | no |
| 23214-92-8 | 59-05-2 | no | 114-07-8 | [7689-03-4](https://china.guidechem.com/15395/detail.html) | no |
| 23214-92-8 | 19660-77-6 | no | 114-07-8 | 7385-67-3 | no |
| 23214-92-8 | [918504-65-1](https://china.guidechem.com/1173516/) | no | 114-07-8 | 50-78-2 | no |
| 23214-92-8 | [934660-93-2](https://china.guidechem.com/1556922/detail.html) | no | 443-48-1 | 100986-85-4 | no |
| 23214-92-8 | 85622-93-1 | no | 443-48-1 | 56-75-7 | no |
| 23214-92-8 | 137234-62-9 | no | 443-48-1 | 57808-66-9 | no |
| 23214-92-8 | 3599-32-4 | no | 443-48-1 | 52549-17-4 | no |
| 23214-92-8 | [14459-29-1](https://china.guidechem.com/114399/detail.html) | no | 443-48-1 | 86386-73-4 | no |
| 318-98-9 | 379231-04-6 | no | 443-48-1 | 81103-11-9 | no |
| 318-98-9 | 380843-75-4 | no | 443-48-1 | 21967-41-9 | no |
| 318-98-9 | 1110813-31-4 | no | 443-48-1 | 3599-32-4 | no |
| 318-98-9 | 557795-19-4 | no | 443-48-1 | [934660-93-2](https://china.guidechem.com/1556922/detail.html) | no |
| 318-98-9 | 1073154-85-4 | no | 443-48-1 | 184475-35-2 | no |
| [58-32-2](https://china.guidechem.com/217/) | 50-78-2 | no | 443-48-1 | 13292-46-1 | no |
| [58-32-2](https://china.guidechem.com/217/) | 63610-08-2 | no | 443-48-1 | 19660-77-6 | no |
| [58-32-2](https://china.guidechem.com/217/) | 73963-72-1 | no | 443-48-1 | 23214-92-8 | no |
| 113665-84-2 | [58-32-2](https://china.guidechem.com/217/) | no | 443-48-1 | [14459-29-1](https://china.guidechem.com/114399/detail.html) | no |
| 113665-84-2 | 274693-27-5 | no | 443-48-1 | [65277-42-1](https://china.guidechem.com/26080/) | no |
| 150322-43-3 | [58-32-2](https://china.guidechem.com/217/) | no | 443-48-1 | [58-32-2](https://china.guidechem.com/217/) | no |
| 150322-43-3 | 274693-27-5 | no | 443-48-1 | 150322-43-3 | no |
| 150322-43-3 | 113665-84-2 | no | 443-48-1 | 113665-84-2 | no |
| 525-66-6 | 1073154-85-4 | no | 443-48-1 | 318-98-9 | no |
| 525-66-6 | 379231-04-6 | no | 443-48-1 | 557795-19-4 | no |
| 525-66-6 | 1110813-31-4 | no | 443-48-1 | 1110813-31-4 | no |
| 525-66-6 | 380843-75-4 | no | 443-48-1 | 380843-75-4 | no |
| 525-66-6 | 557795-19-4 | no | 443-48-1 | [183321-74-6](https://china.guidechem.com/1554622/) | no |
| 57808-66-9 | 26787-78-0 | no | 26787-78-0 | [934660-93-2](https://china.guidechem.com/1556922/detail.html) | no |
| 57808-66-9 | 67-45-8 | no | 26787-78-0 | 184475-35-2 | no |
| 57808-66-9 | 443-48-1 | no | 26787-78-0 | [183321-74-6](https://china.guidechem.com/1554622/) | no |
| 114-07-8 | 86386-73-4 | no | 26787-78-0 | [65277-42-1](https://china.guidechem.com/26080/) | no |
| 114-07-8 | 52549-17-4 | no | 26787-78-0 | [14459-29-1](https://china.guidechem.com/114399/detail.html) | no |
| 114-07-8 | 13292-46-1 | no | 26787-78-0 | 23214-92-8 | no |
| 114-07-8 | 426-13-1 | no | 26787-78-0 | 871700-17-3 | no |
| 13292-46-1 | [7681-93-8](https://china.guidechem.com/15390/) | no | 26787-78-0 | 59-05-2 | no |
| 13292-46-1 | 426-13-1 | no | 26787-78-0 | 1195765-45-7 | no |
| 13292-46-1 | 59277-89-3 | no | 26787-78-0 | 57-62-5 | no |
| 13292-46-1 | 100986-85-4 | no | 26787-78-0 | 81103-11-9 | no |
| 13292-46-1 | 56-75-7 | no | 26787-78-0 | 32986-56-4 | no |
| 52549-17-4 | 32986-56-4 | no | 26787-78-0 | 103577-45-3 | no |
| 52549-17-4 | 13292-46-1 | no | 26787-78-0 | 13292-46-1 | no |
| 32986-56-4 | 13292-46-1 | no | 26787-78-0 | 100986-85-4 | no |
| 32986-56-4 | 56-75-7 | no | 26787-78-0 | 379231-04-6 | no |
| 32986-56-4 | 100986-85-4 | no | 26787-78-0 | 380843-75-4 | no |
| 32986-56-4 | 59277-89-3 | no | 26787-78-0 | 1110813-31-4 | no |
| 32986-56-4 | 426-13-1 | no | 26787-78-0 | 557795-19-4 | no |
| 32986-56-4 | 52549-17-4 | no | 26787-78-0 | 318-98-9 | no |
| [7681-93-8](https://china.guidechem.com/15390/) | 426-13-1 | no | 26787-78-0 | 113665-84-2 | no |
| [7681-93-8](https://china.guidechem.com/15390/) | 56-75-7 | no | 26787-78-0 | 150322-43-3 | no |
| [7681-93-8](https://china.guidechem.com/15390/) | 32986-56-4 | no | 26787-78-0 | 656247-17-5 | no |
| [7681-93-8](https://china.guidechem.com/15390/) | 52549-17-4 | no | 26787-78-0 | [33069-62-4](https://china.guidechem.com/22415/) | no |
| [7681-93-8](https://china.guidechem.com/15390/) | 7385-67-3 | no | 26787-78-0 | [7689-03-4](https://china.guidechem.com/15395/detail.html) | no |
| [7681-93-8](https://china.guidechem.com/15390/) | 1073154-85-4 | no | 26787-78-0 | 7385-67-3 | no |
| [7681-93-8](https://china.guidechem.com/15390/) | 113665-84-2 | no | 26787-78-0 | 19685-09-7 | no |
| [7681-93-8](https://china.guidechem.com/15390/) | 1110813-31-4 | no | 100986-85-4 | 57808-66-9 | no |
| [7681-93-8](https://china.guidechem.com/15390/) | 150322-43-3 | no | 100986-85-4 | 81103-11-9 | no |
| [7681-93-8](https://china.guidechem.com/15390/) | 557795-19-4 | no | 100986-85-4 | 3599-32-4 | no |
| [7681-93-8](https://china.guidechem.com/15390/) | 380843-75-4 | no | 100986-85-4 | [934660-93-2](https://china.guidechem.com/1556922/detail.html) | no |
| [7681-93-8](https://china.guidechem.com/15390/) | [14459-29-1](https://china.guidechem.com/114399/detail.html) | no | 100986-85-4 | 184475-35-2 | no |
| [7681-93-8](https://china.guidechem.com/15390/) | 379231-04-6 | no | 100986-85-4 | [183321-74-6](https://china.guidechem.com/1554622/) | no |
| [7681-93-8](https://china.guidechem.com/15390/) | [918504-65-1](https://china.guidechem.com/1173516/) | no | 100986-85-4 | 379231-04-6 | no |
| [7681-93-8](https://china.guidechem.com/15390/) | 71125-38-7 | no | 100986-85-4 | 103577-45-3 | no |
| [7681-93-8](https://china.guidechem.com/15390/) | 81103-11-9 | no | 100986-85-4 | 23214-92-8 | no |
| [7681-93-8](https://china.guidechem.com/15390/) | 57-62-5 | no | 100986-85-4 | [14459-29-1](https://china.guidechem.com/114399/detail.html) | no |
| [7681-93-8](https://china.guidechem.com/15390/) | 117976-89-3 | no | 100986-85-4 | [58-32-2](https://china.guidechem.com/217/) | no |
| [7681-93-8](https://china.guidechem.com/15390/) | 76824-35-6 | no | 100986-85-4 | 150322-43-3 | no |
| [7681-93-8](https://china.guidechem.com/15390/) | 103577-45-3 | no | 100986-85-4 | 113665-84-2 | no |
| [7681-93-8](https://china.guidechem.com/15390/) | 57808-66-9 | no | 100986-85-4 | 318-98-9 | no |
| [7681-93-8](https://china.guidechem.com/15390/) | 525-66-6 | no | 100986-85-4 | 557795-19-4 | no |
| [7681-93-8](https://china.guidechem.com/15390/) | 67-45-8 | no | 100986-85-4 | 1110813-31-4 | no |
| [7681-93-8](https://china.guidechem.com/15390/) | 26787-78-0 | no | 100986-85-4 | 380843-75-4 | no |
| [7681-93-8](https://china.guidechem.com/15390/) | 443-48-1 | no | 67-45-8 | 150322-43-3 | no |
| 525-66-6 | [934660-93-2](https://china.guidechem.com/1556922/detail.html) | no | 67-45-8 | 113665-84-2 | no |
| 525-66-6 | 3599-32-4 | no | 67-45-8 | 318-98-9 | no |
| 525-66-6 | [14459-29-1](https://china.guidechem.com/114399/detail.html) | no | 67-45-8 | 557795-19-4 | no |
| 525-66-6 | 19660-77-6 | no | 67-45-8 | 1110813-31-4 | no |
| 525-66-6 | 113665-84-2 | no | 67-45-8 | 380843-75-4 | no |
| 525-66-6 | 50-78-2 | no | 67-45-8 | 379231-04-6 | no |
| 525-66-6 | 81103-11-9 | no | 67-45-8 | [183321-74-6](https://china.guidechem.com/1554622/) | no |
| 525-66-6 | 86386-73-4 | no | 67-45-8 | 184475-35-2 | no |
| 525-66-6 | 63610-08-2 | no | 67-45-8 | [65277-42-1](https://china.guidechem.com/26080/) | no |
| 525-66-6 | [7689-03-4](https://china.guidechem.com/15395/detail.html) | no | 67-45-8 | [934660-93-2](https://china.guidechem.com/1556922/detail.html) | no |
| 525-66-6 | 318-98-9 | no | 67-45-8 | 3599-32-4 | no |
| 525-66-6 | 71125-38-7 | no | 67-45-8 | 23214-92-8 | no |
| 525-66-6 | [95058-81-4](https://china.guidechem.com/28627/detail.html) | no | 67-45-8 | 81103-11-9 | no |
| 525-66-6 | 56-75-7 | no | 67-45-8 | 32986-56-4 | no |
| 525-66-6 | 426-13-1 | no | 67-45-8 | 13292-46-1 | no |
| 525-66-6 | 85622-93-1 | no | 67-45-8 | [58-32-2](https://china.guidechem.com/217/) | no |
| 525-66-6 | 73963-72-1 | no | 56-75-7 | 57808-66-9 | no |
| 525-66-6 | 76824-35-6 | no | 56-75-7 | 81103-11-9 | no |
| 525-66-6 | 57808-66-9 | no | 56-75-7 | 23214-92-8 | no |
| 525-66-6 | 13292-46-1 | no | 56-75-7 | 184475-35-2 | no |
| 525-66-6 | 103577-45-3 | no | 56-75-7 | [183321-74-6](https://china.guidechem.com/1554622/) | no |
| 525-66-6 | 184475-35-2 | no | 56-75-7 | [7689-03-4](https://china.guidechem.com/15395/detail.html) | no |
| 525-66-6 | 57-62-5 | no | 56-75-7 | 19685-09-7 | no |
| 525-66-6 | [58-32-2](https://china.guidechem.com/217/) | no | 56-75-7 | 379231-04-6 | no |
| 525-66-6 | 67-45-8 | no | 56-75-7 | 380843-75-4 | no |
| 525-66-6 | 150322-43-3 | no | 56-75-7 | 1110813-31-4 | no |
| 525-66-6 | 19685-09-7 | no | 56-75-7 | 557795-19-4 | no |
| 525-66-6 | [33069-62-4](https://china.guidechem.com/22415/) | no | 56-75-7 | 318-98-9 | no |
| 525-66-6 | [183321-74-6](https://china.guidechem.com/1554622/) | no | 56-75-7 | 113665-84-2 | no |
| 525-66-6 | 50-02-2 | no | 56-75-7 | 150322-43-3 | no |
| 525-66-6 | 274693-27-5 | no | 56-75-7 | [58-32-2](https://china.guidechem.com/217/) | no |
| 525-66-6 | 7385-67-3 | no | 56-75-7 | 103577-45-3 | no |
| 525-66-6 | [60-54-8](https://china.guidechem.com/279/) | no | 56-75-7 | [14459-29-1](https://china.guidechem.com/114399/detail.html) | no |
| 525-66-6 | 137234-62-9 | no | 56-75-7 | [33069-62-4](https://china.guidechem.com/22415/) | no |
| 525-66-6 | [65277-42-1](https://china.guidechem.com/26080/) | no | 13292-46-1 | 57808-66-9 | no |
| 73590-58-6 | [95058-81-4](https://china.guidechem.com/28627/detail.html) | no | 13292-46-1 | 51481-61-9 | no |
| 73590-58-6 | [918504-65-1](https://china.guidechem.com/1173516/) | no | 13292-46-1 | 103577-45-3 | no |
| 73590-58-6 | 71125-38-7 | no | 13292-46-1 | 50-02-2 | no |
| 73590-58-6 | [7681-93-8](https://china.guidechem.com/15390/) | no | 13292-46-1 | 76824-35-6 | no |
| 73590-58-6 | 525-66-6 | no | 13292-46-1 | 81103-11-9 | no |
| 73590-58-6 | 32986-56-4 | no | 13292-46-1 | 57-62-5 | no |
| 73590-58-6 | 114-07-8 | no | 13292-46-1 | 70458-96-7 | no |
| 73590-58-6 | 1195765-45-7 | no | 13292-46-1 | 117976-89-3 | no |
| 73590-58-6 | 81103-11-9 | no | 13292-46-1 | 1195765-45-7 | no |
| 73590-58-6 | [65277-42-1](https://china.guidechem.com/26080/) | no | 13292-46-1 | 137234-62-9 | no |
| 73590-58-6 | 184475-35-2 | no | 13292-46-1 | 21967-41-9 | no |
| 73590-58-6 | [183321-74-6](https://china.guidechem.com/1554622/) | no | 13292-46-1 | 871700-17-3 | no |
| 73590-58-6 | [934660-93-2](https://china.guidechem.com/1556922/detail.html) | no | 13292-46-1 | 19660-77-6 | no |
| 73590-58-6 | 318-98-9 | no | 13292-46-1 | 23214-92-8 | no |
| 73590-58-6 | 1073154-85-4 | no | 13292-46-1 | [14459-29-1](https://china.guidechem.com/114399/detail.html) | no |
| 73590-58-6 | 557795-19-4 | no | 13292-46-1 | 184475-35-2 | no |
| 73590-58-6 | 1110813-31-4 | no | 13292-46-1 | [183321-74-6](https://china.guidechem.com/1554622/) | no |
| 73590-58-6 | 380843-75-4 | no | 13292-46-1 | [918504-65-1](https://china.guidechem.com/1173516/) | no |
| 73590-58-6 | 379231-04-6 | no | 13292-46-1 | [33069-62-4](https://china.guidechem.com/22415/) | no |
| 73590-58-6 | 113665-84-2 | no | 13292-46-1 | [60-54-8](https://china.guidechem.com/279/) | no |
| 73590-58-6 | 150322-43-3 | no | 13292-46-1 | [7689-03-4](https://china.guidechem.com/15395/detail.html) | no |
| 73590-58-6 | 656247-17-5 | no | 13292-46-1 | 7385-67-3 | no |
| 73590-58-6 | [58-32-2](https://china.guidechem.com/217/) | no | 13292-46-1 | 85622-93-1 | no |
| 426-13-1 | 76824-35-6 | no | 13292-46-1 | 19685-09-7 | no |
| 426-13-1 | 51481-61-9 | no | 13292-46-1 | 71125-38-7 | no |
| 426-13-1 | 50-02-2 | no | 13292-46-1 | 379231-04-6 | no |
| 426-13-1 | 57808-66-9 | no | 13292-46-1 | 380843-75-4 | no |
| 426-13-1 | 26787-78-0 | no | 13292-46-1 | 1110813-31-4 | no |
| 426-13-1 | 3599-32-4 | no | 13292-46-1 | 557795-19-4 | no |
| 426-13-1 | 81103-11-9 | no | 13292-46-1 | 1073154-85-4 | no |
| 426-13-1 | 70458-96-7 | no | 13292-46-1 | 50-78-2 | no |
| 13292-46-1 | 113665-84-2 | no | 51481-61-9 | 113665-84-2 | no |
| 13292-46-1 | 150322-43-3 | no | 51481-61-9 | [58-32-2](https://china.guidechem.com/217/) | no |
| 13292-46-1 | 274693-27-5 | no | 51481-61-9 | 150322-43-3 | no |
| 13292-46-1 | 73963-72-1 | no | 51481-61-9 | 379231-04-6 | no |
| 13292-46-1 | 63610-08-2 | no | 51481-61-9 | 557795-19-4 | no |
| 13292-46-1 | 656247-17-5 | no | 51481-61-9 | [65277-42-1](https://china.guidechem.com/26080/) | no |
| 13292-46-1 | [58-32-2](https://china.guidechem.com/217/) | no | 51481-61-9 | [14459-29-1](https://china.guidechem.com/114399/detail.html) | no |
| 57808-66-9 | 19660-77-6 | no | 51481-61-9 | 318-98-9 | no |
| 57808-66-9 | [14459-29-1](https://china.guidechem.com/114399/detail.html) | no | 51481-61-9 | 3599-32-4 | no |
| 57808-66-9 | [60-54-8](https://china.guidechem.com/279/) | no | 318-98-9 | 50-78-2 | no |
| 57808-66-9 | 7385-67-3 | no | 318-98-9 | 656247-17-5 | no |
| 57808-66-9 | 51481-61-9 | no | 318-98-9 | [58-32-2](https://china.guidechem.com/217/) | no |
| 57808-66-9 | 50-02-2 | no | 318-98-9 | 150322-43-3 | no |
| 57808-66-9 | 32986-56-4 | no | 318-98-9 | 63610-08-2 | no |
| 57808-66-9 | 52549-17-4 | no | 23214-92-8 | 50-78-2 | no |
| 57808-66-9 | 86386-73-4 | no | 23214-92-8 | 1110813-31-4 | no |
| 57808-66-9 | 81103-11-9 | no | 23214-92-8 | 113665-84-2 | no |
| 57808-66-9 | 57-62-5 | no | 23214-92-8 | 70458-96-7 | no |
| 57808-66-9 | 117976-89-3 | no | 23214-92-8 | [58-32-2](https://china.guidechem.com/217/) | no |
| 57808-66-9 | 1195765-45-7 | no | 23214-92-8 | 150322-43-3 | no |
| 57808-66-9 | 137234-62-9 | no | 23214-92-8 | 557795-19-4 | no |
| 57808-66-9 | 21967-41-9 | no | 23214-92-8 | 274693-27-5 | no |
| 57808-66-9 | 59-05-2 | no | 23214-92-8 | 318-98-9 | no |
| 57808-66-9 | 871700-17-3 | no | 1195765-45-7 | 23214-92-8 | no |
| 57808-66-9 | 23214-92-8 | no | 1195765-45-7 | [183321-74-6](https://china.guidechem.com/1554622/) | no |
| 57808-66-9 | 3599-32-4 | no | 1195765-45-7 | 380843-75-4 | no |
| 57808-66-9 | [934660-93-2](https://china.guidechem.com/1556922/detail.html) | no | 1195765-45-7 | 1110813-31-4 | no |
| 57808-66-9 | [65277-42-1](https://china.guidechem.com/26080/) | no | 1195765-45-7 | 85622-93-1 | no |
| 57808-66-9 | 184475-35-2 | no | 1195765-45-7 | 184475-35-2 | no |
| 57808-66-9 | [918504-65-1](https://china.guidechem.com/1173516/) | no | 1195765-45-7 | 59-05-2 | no |
| 57808-66-9 | [7689-03-4](https://china.guidechem.com/15395/detail.html) | no | 1195765-45-7 | [934660-93-2](https://china.guidechem.com/1556922/detail.html) | no |
| 57808-66-9 | 85622-93-1 | no | 1195765-45-7 | 113665-84-2 | no |
| 57808-66-9 | 19685-09-7 | no | 1195765-45-7 | 656247-17-5 | no |
| 57808-66-9 | 71125-38-7 | no | 1195765-45-7 | [58-32-2](https://china.guidechem.com/217/) | no |
| 57808-66-9 | 379231-04-6 | no | 1195765-45-7 | 150322-43-3 | no |
| 57808-66-9 | 380843-75-4 | no | 1195765-45-7 | 19685-09-7 | no |
| 57808-66-9 | 1110813-31-4 | no | 1195765-45-7 | 379231-04-6 | no |
| 57808-66-9 | 557795-19-4 | no | 1195765-45-7 | 557795-19-4 | no |
| 57808-66-9 | 1073154-85-4 | no | 1195765-45-7 | 274693-27-5 | no |
| 57808-66-9 | 318-98-9 | no | 1195765-45-7 | [65277-42-1](https://china.guidechem.com/26080/) | no |
| 57808-66-9 | 50-78-2 | no | 1195765-45-7 | [14459-29-1](https://china.guidechem.com/114399/detail.html) | no |
| 57808-66-9 | 113665-84-2 | no | 1195765-45-7 | 318-98-9 | no |
| 57808-66-9 | 274693-27-5 | no | 137234-62-9 | [183321-74-6](https://china.guidechem.com/1554622/) | no |
| 57808-66-9 | 73963-72-1 | no | 137234-62-9 | 1110813-31-4 | no |
| 57808-66-9 | 63610-08-2 | no | 137234-62-9 | 113665-84-2 | no |
| 57808-66-9 | [58-32-2](https://china.guidechem.com/217/) | no | 137234-62-9 | [58-32-2](https://china.guidechem.com/217/) | no |
| 103577-45-3 | 57-62-5 | no | 137234-62-9 | 150322-43-3 | no |
| 103577-45-3 | 19685-09-7 | no | 137234-62-9 | 379231-04-6 | no |
| 103577-45-3 | 71125-38-7 | no | 137234-62-9 | 557795-19-4 | no |
| 103577-45-3 | 50-78-2 | no | 137234-62-9 | [65277-42-1](https://china.guidechem.com/26080/) | no |
| 103577-45-3 | [58-32-2](https://china.guidechem.com/217/) | no | 137234-62-9 | [14459-29-1](https://china.guidechem.com/114399/detail.html) | no |
| 103577-45-3 | 656247-17-5 | no | 137234-62-9 | 318-98-9 | no |
| 103577-45-3 | 63610-08-2 | no | 137234-62-9 | 3599-32-4 | no |
| 103577-45-3 | 73963-72-1 | no | 86386-73-4 | 113665-84-2 | no |
| 103577-45-3 | 274693-27-5 | no | 86386-73-4 | 656247-17-5 | no |
| 103577-45-3 | 150322-43-3 | no | 86386-73-4 | [58-32-2](https://china.guidechem.com/217/) | no |
| 103577-45-3 | 113665-84-2 | no | 86386-73-4 | 150322-43-3 | no |
| 103577-45-3 | 318-98-9 | no | 86386-73-4 | 318-98-9 | no |
| 103577-45-3 | 1073154-85-4 | no | 86386-73-4 | 19660-77-6 | no |
| 103577-45-3 | 557795-19-4 | no | 86386-73-4 | [14459-29-1](https://china.guidechem.com/114399/detail.html) | no |
| 103577-45-3 | 1110813-31-4 | no | 86386-73-4 | [33069-62-4](https://china.guidechem.com/22415/) | no |
| 103577-45-3 | 380843-75-4 | no | 21967-41-9 | 23214-92-8 | no |
| 103577-45-3 | 379231-04-6 | no | 21967-41-9 | [183321-74-6](https://china.guidechem.com/1554622/) | no |
| 103577-45-3 | 7385-67-3 | no | 21967-41-9 | 380843-75-4 | no |
| 103577-45-3 | [7689-03-4](https://china.guidechem.com/15395/detail.html) | no | 21967-41-9 | 1110813-31-4 | no |
| 103577-45-3 | [60-54-8](https://china.guidechem.com/279/) | no | 21967-41-9 | 184475-35-2 | no |
| 103577-45-3 | [918504-65-1](https://china.guidechem.com/1173516/) | no | 21967-41-9 | [934660-93-2](https://china.guidechem.com/1556922/detail.html) | no |
| 103577-45-3 | [183321-74-6](https://china.guidechem.com/1554622/) | no | 21967-41-9 | 113665-84-2 | no |
| 103577-45-3 | [95058-81-4](https://china.guidechem.com/28627/detail.html) | no | 21967-41-9 | [58-32-2](https://china.guidechem.com/217/) | no |
| 103577-45-3 | 184475-35-2 | no | 21967-41-9 | 379231-04-6 | no |
| 103577-45-3 | [65277-42-1](https://china.guidechem.com/26080/) | no | 21967-41-9 | 3599-32-4 | no |
| 103577-45-3 | [934660-93-2](https://china.guidechem.com/1556922/detail.html) | no | 21967-41-9 | 19660-77-6 | no |
| 103577-45-3 | [14459-29-1](https://china.guidechem.com/114399/detail.html) | no | 21967-41-9 | [14459-29-1](https://china.guidechem.com/114399/detail.html) | no |
| 103577-45-3 | 23214-92-8 | no | 21967-41-9 | [7689-03-4](https://china.guidechem.com/15395/detail.html) | no |
| 103577-45-3 | 871700-17-3 | no | 21967-41-9 | 19685-09-7 | no |
| 103577-45-3 | 59-05-2 | no | 59-05-2 | 380843-75-4 | no |
| 103577-45-3 | 21967-41-9 | no | 59-05-2 | 379231-04-6 | no |
| 103577-45-3 | 137234-62-9 | no | 59-05-2 | [65277-42-1](https://china.guidechem.com/26080/) | no |
| 103577-45-3 | 1195765-45-7 | no | 57-62-5 | [183321-74-6](https://china.guidechem.com/1554622/) | no |
| 103577-45-3 | 117976-89-3 | no | 57-62-5 | 380843-75-4 | no |
| 103577-45-3 | 70458-96-7 | no | 57-62-5 | 1195765-45-7 | no |
| 103577-45-3 | 81103-11-9 | no | 57-62-5 | 1110813-31-4 | no |
| 103577-45-3 | 86386-73-4 | no | 57-62-5 | 1073154-85-4 | no |
| 103577-45-3 | 52549-17-4 | no | 57-62-5 | 184475-35-2 | no |
| 103577-45-3 | 32986-56-4 | no | 57-62-5 | [934660-93-2](https://china.guidechem.com/1556922/detail.html) | no |
| 103577-45-3 | 50-02-2 | no | 57-62-5 | 113665-84-2 | no |
| 103577-45-3 | 51481-61-9 | no | 57-62-5 | [58-32-2](https://china.guidechem.com/217/) | no |
| 51481-61-9 | 81103-11-9 | no | 57-62-5 | 150322-43-3 | no |
| 51481-61-9 | 32986-56-4 | no | 57-62-5 | 379231-04-6 | no |
| 50-78-2 | 150322-43-3 | no | 57-62-5 | 557795-19-4 | no |
| 1073154-85-4 | 50-78-2 | no | 57-62-5 | [65277-42-1](https://china.guidechem.com/26080/) | no |
| 1073154-85-4 | 113665-84-2 | no | 57-62-5 | 318-98-9 | no |
| 1073154-85-4 | [58-32-2](https://china.guidechem.com/217/) | no | 57-62-5 | 19660-77-6 | no |
| 1073154-85-4 | 150322-43-3 | no | 57-62-5 | 23214-92-8 | no |
| 1073154-85-4 | 656247-17-5 | no | 57-62-5 | [14459-29-1](https://china.guidechem.com/114399/detail.html) | no |
| 50-02-2 | [183321-74-6](https://china.guidechem.com/1554622/) | no | [934660-93-2](https://china.guidechem.com/1556922/detail.html) | 71125-38-7 | no |
| 50-02-2 | 380843-75-4 | no | [934660-93-2](https://china.guidechem.com/1556922/detail.html) | [58-32-2](https://china.guidechem.com/217/) | no |
| 50-02-2 | 1110813-31-4 | no | [934660-93-2](https://china.guidechem.com/1556922/detail.html) | 274693-27-5 | no |
| 50-02-2 | 1073154-85-4 | no | [934660-93-2](https://china.guidechem.com/1556922/detail.html) | 73963-72-1 | no |
| 50-02-2 | 184475-35-2 | no | [934660-93-2](https://china.guidechem.com/1556922/detail.html) | 318-98-9 | no |
| 50-02-2 | [934660-93-2](https://china.guidechem.com/1556922/detail.html) | no | [934660-93-2](https://china.guidechem.com/1556922/detail.html) | 63610-08-2 | no |
| 50-02-2 | 81103-11-9 | no | 81103-11-9 | 19660-77-6 | no |
| 50-02-2 | 113665-84-2 | no | 81103-11-9 | 23214-92-8 | no |
| 50-02-2 | [58-32-2](https://china.guidechem.com/217/) | no | 81103-11-9 | [14459-29-1](https://china.guidechem.com/114399/detail.html) | no |
| 50-02-2 | 150322-43-3 | no | 81103-11-9 | 50-78-2 | no |
| 50-02-2 | 379231-04-6 | no | 81103-11-9 | [183321-74-6](https://china.guidechem.com/1554622/) | no |
| 50-02-2 | 557795-19-4 | no | 81103-11-9 | 1195765-45-7 | no |
| 50-02-2 | [65277-42-1](https://china.guidechem.com/26080/) | no | 81103-11-9 | 1110813-31-4 | no |
| 50-02-2 | 32986-56-4 | no | 81103-11-9 | 1073154-85-4 | no |
| 50-02-2 | 318-98-9 | no | 81103-11-9 | 85622-93-1 | no |
| 50-02-2 | 3599-32-4 | no | 81103-11-9 | 137234-62-9 | no |
| 50-02-2 | 23214-92-8 | no | 81103-11-9 | 21967-41-9 | no |
| 50-02-2 | [14459-29-1](https://china.guidechem.com/114399/detail.html) | no | 81103-11-9 | 184475-35-2 | no |
| 50-02-2 | [33069-62-4](https://china.guidechem.com/22415/) | no | 81103-11-9 | [95058-81-4](https://china.guidechem.com/28627/detail.html) | no |
| 76824-35-6 | 23214-92-8 | no | 81103-11-9 | 59-05-2 | no |
| 76824-35-6 | [183321-74-6](https://china.guidechem.com/1554622/) | no | 81103-11-9 | 57-62-5 | no |
| 76824-35-6 | 1110813-31-4 | no | 81103-11-9 | [934660-93-2](https://china.guidechem.com/1556922/detail.html) | no |
| 76824-35-6 | 184475-35-2 | no | 81103-11-9 | 117976-89-3 | no |
| 76824-35-6 | [934660-93-2](https://china.guidechem.com/1556922/detail.html) | no | 81103-11-9 | 113665-84-2 | no |
| 76824-35-6 | 81103-11-9 | no | 81103-11-9 | 71125-38-7 | no |
| 76824-35-6 | 113665-84-2 | no | 81103-11-9 | 656247-17-5 | no |
| 76824-35-6 | [58-32-2](https://china.guidechem.com/217/) | no | 81103-11-9 | 7385-67-3 | no |
| 76824-35-6 | 150322-43-3 | no | 81103-11-9 | 70458-96-7 | no |
| 76824-35-6 | 379231-04-6 | no | 81103-11-9 | [58-32-2](https://china.guidechem.com/217/) | no |
| 76824-35-6 | 557795-19-4 | no | 81103-11-9 | 150322-43-3 | no |
| 76824-35-6 | [65277-42-1](https://china.guidechem.com/26080/) | no | 81103-11-9 | 871700-17-3 | no |
| 76824-35-6 | [14459-29-1](https://china.guidechem.com/114399/detail.html) | no | 81103-11-9 | 379231-04-6 | no |
| 76824-35-6 | 318-98-9 | no | 81103-11-9 | 557795-19-4 | no |
| 76824-35-6 | 3599-32-4 | no | 81103-11-9 | [60-54-8](https://china.guidechem.com/279/) | no |
| 86386-73-4 | 23214-92-8 | no | 81103-11-9 | 274693-27-5 | no |
| 86386-73-4 | [183321-74-6](https://china.guidechem.com/1554622/) | no | 81103-11-9 | [65277-42-1](https://china.guidechem.com/26080/) | no |
| 86386-73-4 | 1110813-31-4 | no | 81103-11-9 | [918504-65-1](https://china.guidechem.com/1173516/) | no |
| 86386-73-4 | 184475-35-2 | no | 81103-11-9 | 73963-72-1 | no |
| 86386-73-4 | [934660-93-2](https://china.guidechem.com/1556922/detail.html) | no | 81103-11-9 | 318-98-9 | no |
| 86386-73-4 | 81103-11-9 | no | 81103-11-9 | 63610-08-2 | no |
| 86386-73-4 | 379231-04-6 | no | 81103-11-9 | 3599-32-4 | no |
| 86386-73-4 | 557795-19-4 | no | 81103-11-9 | [33069-62-4](https://china.guidechem.com/22415/) | no |
| 86386-73-4 | 3599-32-4 | no | 81103-11-9 | [7689-03-4](https://china.guidechem.com/15395/detail.html) | no |
| 52549-17-4 | 23214-92-8 | no | 81103-11-9 | 19685-09-7 | no |
| 52549-17-4 | [183321-74-6](https://china.guidechem.com/1554622/) | no | 117976-89-3 | 19660-77-6 | no |
| 52549-17-4 | 184475-35-2 | no | 117976-89-3 | 23214-92-8 | no |
| 52549-17-4 | [934660-93-2](https://china.guidechem.com/1556922/detail.html) | no | 117976-89-3 | [183321-74-6](https://china.guidechem.com/1554622/) | no |
| 52549-17-4 | 81103-11-9 | no | 117976-89-3 | 380843-75-4 | no |
| 52549-17-4 | 113665-84-2 | no | 117976-89-3 | 1195765-45-7 | no |
| 52549-17-4 | [58-32-2](https://china.guidechem.com/217/) | no | 117976-89-3 | 1110813-31-4 | no |
| 52549-17-4 | 150322-43-3 | no | 117976-89-3 | 1073154-85-4 | no |
| 52549-17-4 | 379231-04-6 | no | 117976-89-3 | 184475-35-2 | no |
| 52549-17-4 | [14459-29-1](https://china.guidechem.com/114399/detail.html) | no | 117976-89-3 | [95058-81-4](https://china.guidechem.com/28627/detail.html) | no |
| 52549-17-4 | 318-98-9 | no | 117976-89-3 | 59-05-2 | no |
| 52549-17-4 | 3599-32-4 | no | 117976-89-3 | [934660-93-2](https://china.guidechem.com/1556922/detail.html) | no |
| 557795-19-4 | 50-78-2 | no | 117976-89-3 | 113665-84-2 | no |
| 557795-19-4 | 1073154-85-4 | no | 117976-89-3 | 656247-17-5 | no |
| 557795-19-4 | 113665-84-2 | no | 117976-89-3 | [58-32-2](https://china.guidechem.com/217/) | no |
| 557795-19-4 | [58-32-2](https://china.guidechem.com/217/) | no | 117976-89-3 | 150322-43-3 | no |
| 557795-19-4 | 150322-43-3 | no | 117976-89-3 | 379231-04-6 | no |
| 557795-19-4 | 63610-08-2 | no | 117976-89-3 | 557795-19-4 | no |
| 32986-56-4 | 1195765-45-7 | no | 117976-89-3 | [65277-42-1](https://china.guidechem.com/26080/) | no |
| 32986-56-4 | 137234-62-9 | no | 117976-89-3 | 318-98-9 | no |
| 32986-56-4 | 57-62-5 | no | 117976-89-3 | 3599-32-4 | no |
| 32986-56-4 | 81103-11-9 | no | 117976-89-3 | [14459-29-1](https://china.guidechem.com/114399/detail.html) | no |
| 32986-56-4 | 117976-89-3 | no | 117976-89-3 | [33069-62-4](https://china.guidechem.com/22415/) | no |
| 32986-56-4 | 656247-17-5 | no | 117976-89-3 | [7689-03-4](https://china.guidechem.com/15395/detail.html) | no |
| 32986-56-4 | 70458-96-7 | no | 117976-89-3 | 19685-09-7 | no |
| 32986-56-4 | 557795-19-4 | no | 70458-96-7 | 23214-92-8 | no |
| 32986-56-4 | 63610-08-2 | no | 70458-96-7 | [183321-74-6](https://china.guidechem.com/1554622/) | no |
| 32986-56-4 | 76824-35-6 | no | 70458-96-7 | 380843-75-4 | no |
| 32986-56-4 | 21967-41-9 | no | 70458-96-7 | 1195765-45-7 | no |
| 32986-56-4 | 59-05-2 | no | 70458-96-7 | 1110813-31-4 | no |
| 32986-56-4 | 871700-17-3 | no | 70458-96-7 | 1073154-85-4 | no |
| 32986-56-4 | 19660-77-6 | no | 70458-96-7 | 184475-35-2 | no |
| 32986-56-4 | 23214-92-8 | no | 70458-96-7 | [934660-93-2](https://china.guidechem.com/1556922/detail.html) | no |
| 32986-56-4 | [14459-29-1](https://china.guidechem.com/114399/detail.html) | no | 70458-96-7 | 113665-84-2 | no |
| 32986-56-4 | 3599-32-4 | no | 70458-96-7 | 7385-67-3 | no |
| 32986-56-4 | [934660-93-2](https://china.guidechem.com/1556922/detail.html) | no | 70458-96-7 | [58-32-2](https://china.guidechem.com/217/) | no |
| 32986-56-4 | 184475-35-2 | no | 70458-96-7 | 150322-43-3 | no |
| 32986-56-4 | [95058-81-4](https://china.guidechem.com/28627/detail.html) | no | 70458-96-7 | 379231-04-6 | no |
| 32986-56-4 | [183321-74-6](https://china.guidechem.com/1554622/) | no | 70458-96-7 | 557795-19-4 | no |
| 32986-56-4 | [918504-65-1](https://china.guidechem.com/1173516/) | no | 70458-96-7 | [65277-42-1](https://china.guidechem.com/26080/) | no |
| 32986-56-4 | [33069-62-4](https://china.guidechem.com/22415/) | no | 70458-96-7 | 318-98-9 | no |
| 32986-56-4 | [60-54-8](https://china.guidechem.com/279/) | no | 70458-96-7 | 21967-41-9 | no |
| 32986-56-4 | [7689-03-4](https://china.guidechem.com/15395/detail.html) | no | 70458-96-7 | 19660-77-6 | no |
| 32986-56-4 | 7385-67-3 | no | 70458-96-7 | [14459-29-1](https://china.guidechem.com/114399/detail.html) | no |
| 32986-56-4 | 85622-93-1 | no | 70458-96-7 | [33069-62-4](https://china.guidechem.com/22415/) | no |
| 32986-56-4 | 19685-09-7 | no | 70458-96-7 | [7689-03-4](https://china.guidechem.com/15395/detail.html) | no |
| 32986-56-4 | 71125-38-7 | no | 70458-96-7 | 19685-09-7 | no |
| 32986-56-4 | 379231-04-6 | no | [65277-42-1](https://china.guidechem.com/26080/) | 184475-35-2 | no |
| 32986-56-4 | 380843-75-4 | no | [65277-42-1](https://china.guidechem.com/26080/) | [95058-81-4](https://china.guidechem.com/28627/detail.html) | no |
| 32986-56-4 | 1110813-31-4 | no | 3599-32-4 | 50-78-2 | no |
| 32986-56-4 | 1073154-85-4 | no | 3599-32-4 | 150322-43-3 | no |
| 32986-56-4 | 318-98-9 | no | 3599-32-4 | 274693-27-5 | no |
| 32986-56-4 | 113665-84-2 | no | 3599-32-4 | [7689-03-4](https://china.guidechem.com/15395/detail.html) | no |
| 32986-56-4 | 274693-27-5 | no | 3599-32-4 | 318-98-9 | no |
| 32986-56-4 | 73963-72-1 | no | 3599-32-4 | 63610-08-2 | no |
| 32986-56-4 | [58-32-2](https://china.guidechem.com/217/) | no | 3599-32-4 | 7385-67-3 | no |
| 51481-61-9 | 23214-92-8 | no | 3599-32-4 | 1110813-31-4 | no |
| 51481-61-9 | [183321-74-6](https://china.guidechem.com/1554622/) | no | 3599-32-4 | 656247-17-5 | no |
| 51481-61-9 | 380843-75-4 | no | 59-05-2 | 1110813-31-4 | no |
| 51481-61-9 | 1110813-31-4 | no | 59-05-2 | 557795-19-4 | no |
| 51481-61-9 | 184475-35-2 | no | 59-05-2 | 1073154-85-4 | no |
| 51481-61-9 | [934660-93-2](https://china.guidechem.com/1556922/detail.html) | no | 59-05-2 | 318-98-9 | no |
| 59-05-2 | 113665-84-2 | no | 23214-92-8 | [7689-03-4](https://china.guidechem.com/15395/detail.html) | no |
| 59-05-2 | 150322-43-3 | no | 23214-92-8 | 73963-72-1 | no |
| 59-05-2 | [58-32-2](https://china.guidechem.com/217/) | no | 23214-92-8 | 63610-08-2 | no |
| 871700-17-3 | [65277-42-1](https://china.guidechem.com/26080/) | no | 184475-35-2 | [58-32-2](https://china.guidechem.com/217/) | no |
| 871700-17-3 | 380843-75-4 | no | 184475-35-2 | 85622-93-1 | no |
| 871700-17-3 | 557795-19-4 | no | 184475-35-2 | 71125-38-7 | no |
| 871700-17-3 | 19685-09-7 | no | 184475-35-2 | 318-98-9 | no |
| 19660-77-6 | [65277-42-1](https://china.guidechem.com/26080/) | no | 184475-35-2 | 50-78-2 | no |
| 19660-77-6 | 379231-04-6 | no | 184475-35-2 | 113665-84-2 | no |
| 19660-77-6 | 380843-75-4 | no | 184475-35-2 | 150322-43-3 | no |
| 19660-77-6 | 1110813-31-4 | no | 184475-35-2 | 274693-27-5 | no |
| 19660-77-6 | 557795-19-4 | no | 184475-35-2 | 7385-67-3 | no |
| 19660-77-6 | 1073154-85-4 | no | 184475-35-2 | 19685-09-7 | no |
| 19660-77-6 | 656247-17-5 | no | [14459-29-1](https://china.guidechem.com/114399/detail.html) | [65277-42-1](https://china.guidechem.com/26080/) | no |
| 19660-77-6 | [58-32-2](https://china.guidechem.com/217/) | no | [14459-29-1](https://china.guidechem.com/114399/detail.html) | [95058-81-4](https://china.guidechem.com/28627/detail.html) | no |
| 19660-77-6 | [14459-29-1](https://china.guidechem.com/114399/detail.html) | no | [14459-29-1](https://china.guidechem.com/114399/detail.html) | [60-54-8](https://china.guidechem.com/279/) | no |
| 19660-77-6 | 318-98-9 | no | [14459-29-1](https://china.guidechem.com/114399/detail.html) | [7689-03-4](https://china.guidechem.com/15395/detail.html) | no |
| 23214-92-8 | [65277-42-1](https://china.guidechem.com/26080/) | no | [14459-29-1](https://china.guidechem.com/114399/detail.html) | 7385-67-3 | no |
| 23214-92-8 | [95058-81-4](https://china.guidechem.com/28627/detail.html) | no | [14459-29-1](https://china.guidechem.com/114399/detail.html) | 85622-93-1 | no |
| 23214-92-8 | [60-54-8](https://china.guidechem.com/279/) | no | [14459-29-1](https://china.guidechem.com/114399/detail.html) | 71125-38-7 | no |
| 23214-92-8 | 71125-38-7 | no | [14459-29-1](https://china.guidechem.com/114399/detail.html) | 379231-04-6 | no |
| 23214-92-8 | 379231-04-6 | no | [14459-29-1](https://china.guidechem.com/114399/detail.html) | 380843-75-4 | no |
| 23214-92-8 | 380843-75-4 | no | [14459-29-1](https://china.guidechem.com/114399/detail.html) | 150322-43-3 | no |
| 23214-92-8 | [33069-62-4](https://china.guidechem.com/22415/) | no |  |  |  |

**Table S3.** **List of Non-asemblable LA-based Ternary Nanomedicine Combinations.** This table lists the drug combinations that failed to form stable nanoparticles (i.e., not meeting the criteria of particle size < 350 nm and PDI < 0.2) when attempted for co-assembly with LA. The columns indicate the CAS numbers of the two small molecule drugs (Drug1, Drug2) and the outcome (Yes/No for assembly, here always 'No')..

| Rank | Drug A | Drug B | Size (nm) | PDI |
| --- | --- | --- | --- | --- |
| 1 | 71125-38-7 | 63610-08-2 | 128.67±1.22 | 0.024±0.013 |
| 2 | 71125-38-7 | 60-54-8 | 191.50±1.91 | 0.037±0.02 |
| 3 | 60-54-8 | 63610-08-2 | 209.53±3.65 | 0.025±0.017 |
| 4 | 85622-93-1 | 73963-72-1 | 150.67±2.90 | 0.021±0.015 |
| 5 | 73963-72-1 | 63610-08-2 | 150.80±3.31 | 0.013±0.019 |
| 6 | 60-54-8 | 918504-65-1 | 207.67±3.16 | 0.012±0.015 |
| 7 | 60-54-8 | 67-45-8 | 104.50±2.36 | 0.015±0.015 |
| 8 | 85622-93-1 | 60-54-8 | 183.63±3.30 | 0.03±0.013 |
| 9 | 59277-89-3 | 63610-08-2 | 90.11±1.52 | 0.055±0.044 |
| 10 | 73963-72-1 | 71125-38-7 | 125.00±3.02 | 0.033±0.005 |

**Table S4. Hydrodynamic diameter and PDI of the top-ten predicted assemblies were determined.**
