# Supplementary figures and images for: Machine-Learning-Accelerated Design of Ternary Carrier-Free Nanomedicine for Intranasal Therapy of Brain Metastatic Non-small-cell Lung Cancer
Source: Research (Wash D C). 2026 Mar 13;9:1180. doi: 10.34133/research.1180 (PMC12982895; doi:10.34133/research.1180)

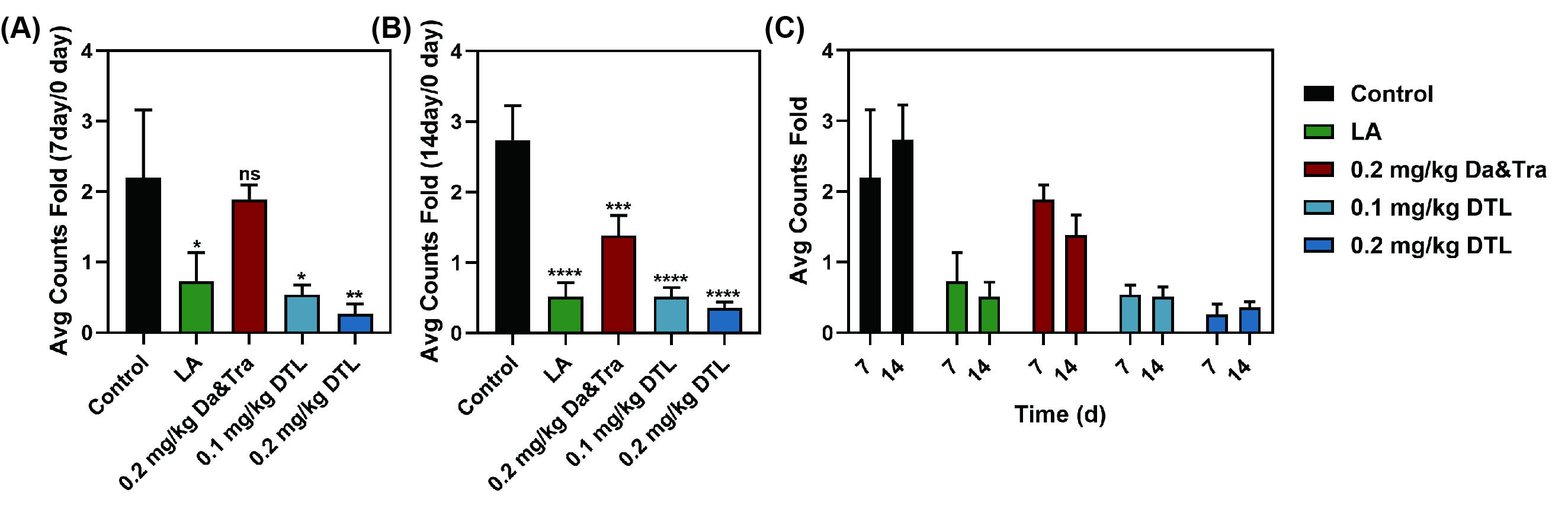

Supplement: Supplementary 1 — Experimental procedures Figs. S1 to S13 Tables S1 to S5 [file research.1180.f1.zip › Figure S10.jpg]

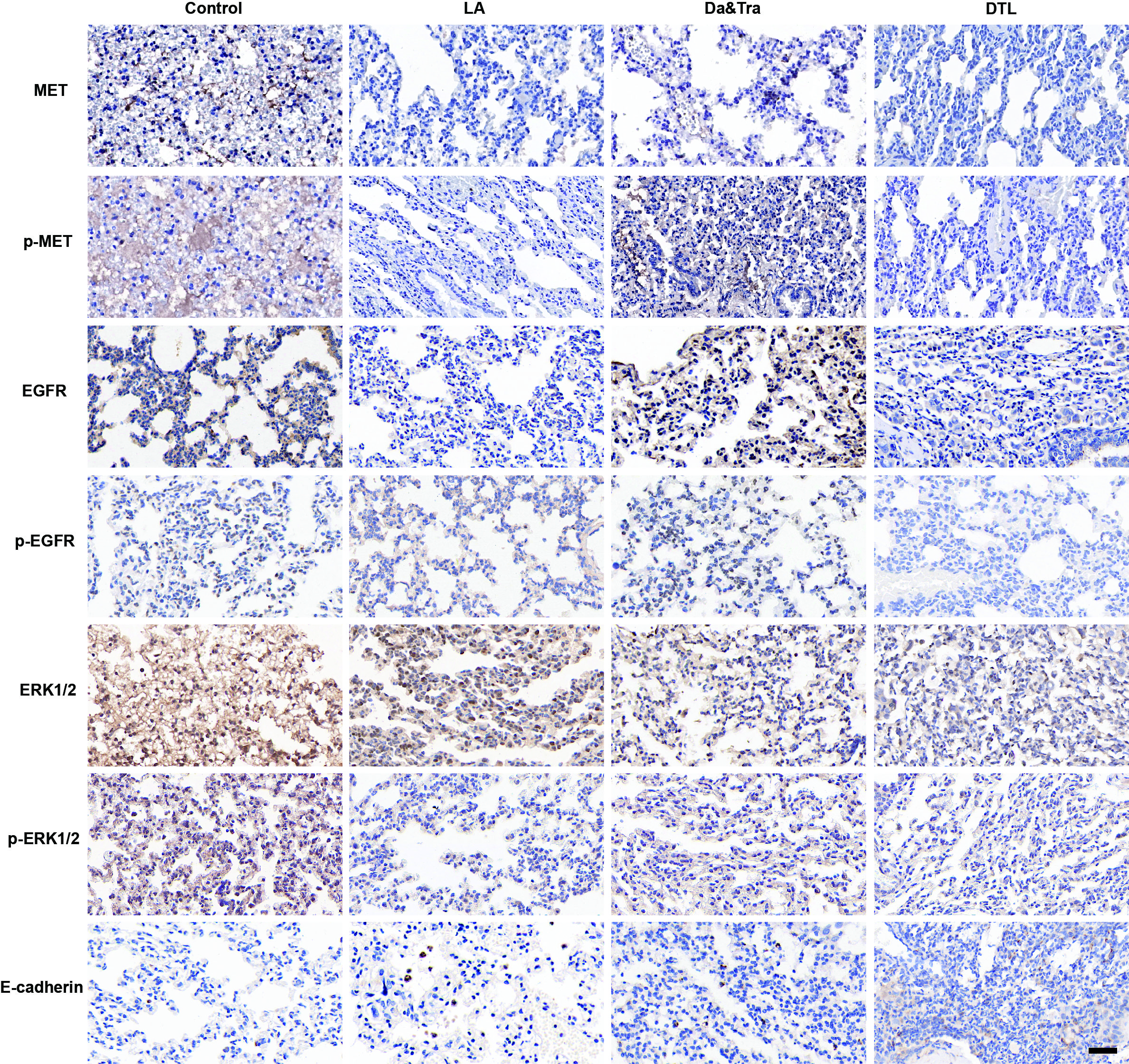

Supplement: Supplementary 1 — Experimental procedures Figs. S1 to S13 Tables S1 to S5 [file research.1180.f1.zip › Figure S11.jpg]

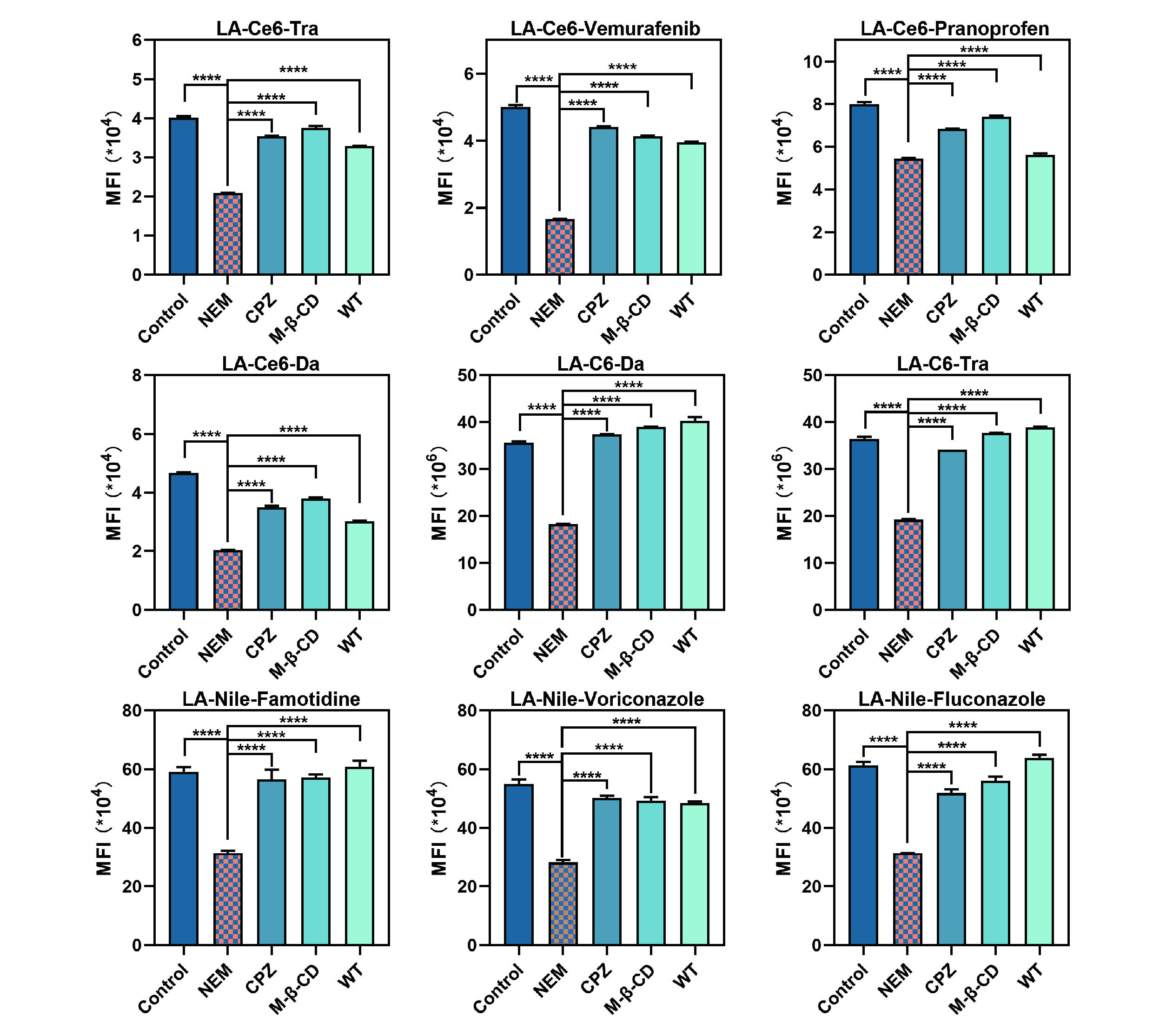

Supplement: Supplementary 1 — Experimental procedures Figs. S1 to S13 Tables S1 to S5 [file research.1180.f1.zip › Figure S1_ 1.jpg]

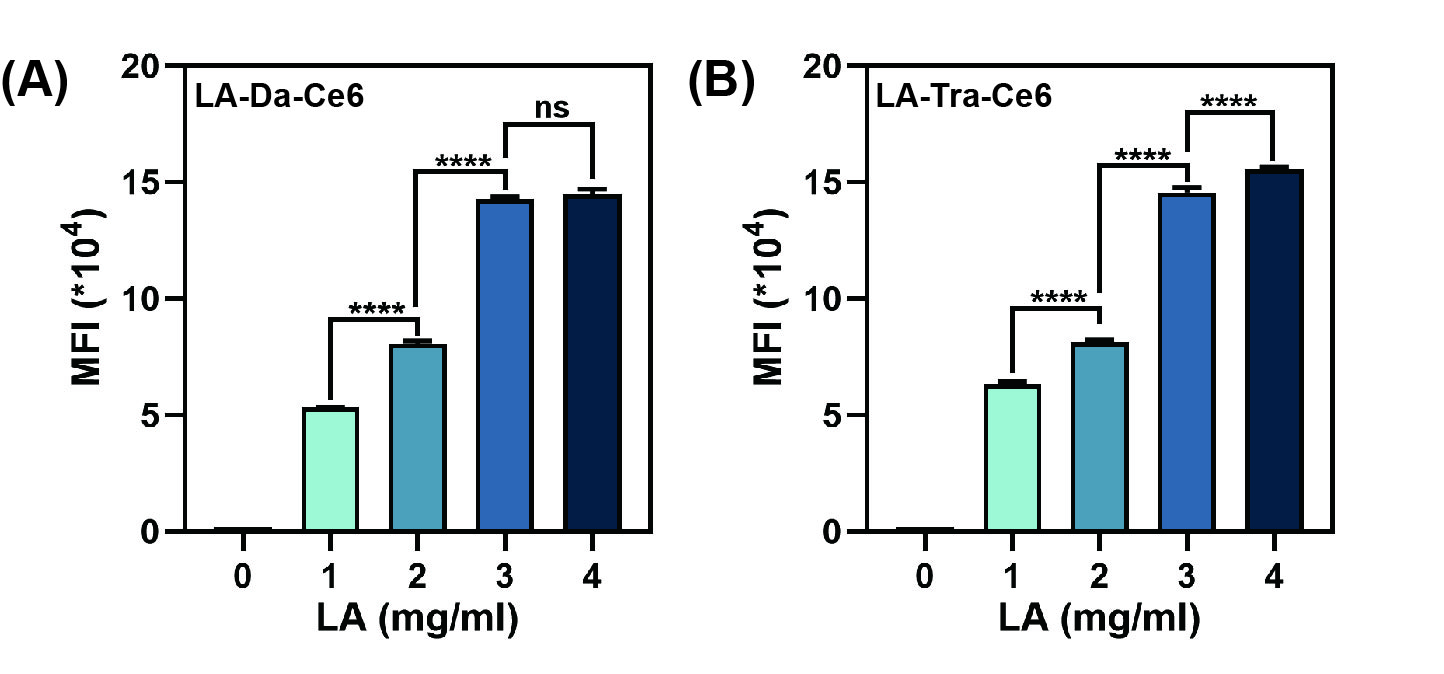

Supplement: Supplementary 1 — Experimental procedures Figs. S1 to S13 Tables S1 to S5 [file research.1180.f1.zip › Figure S2.jpg]

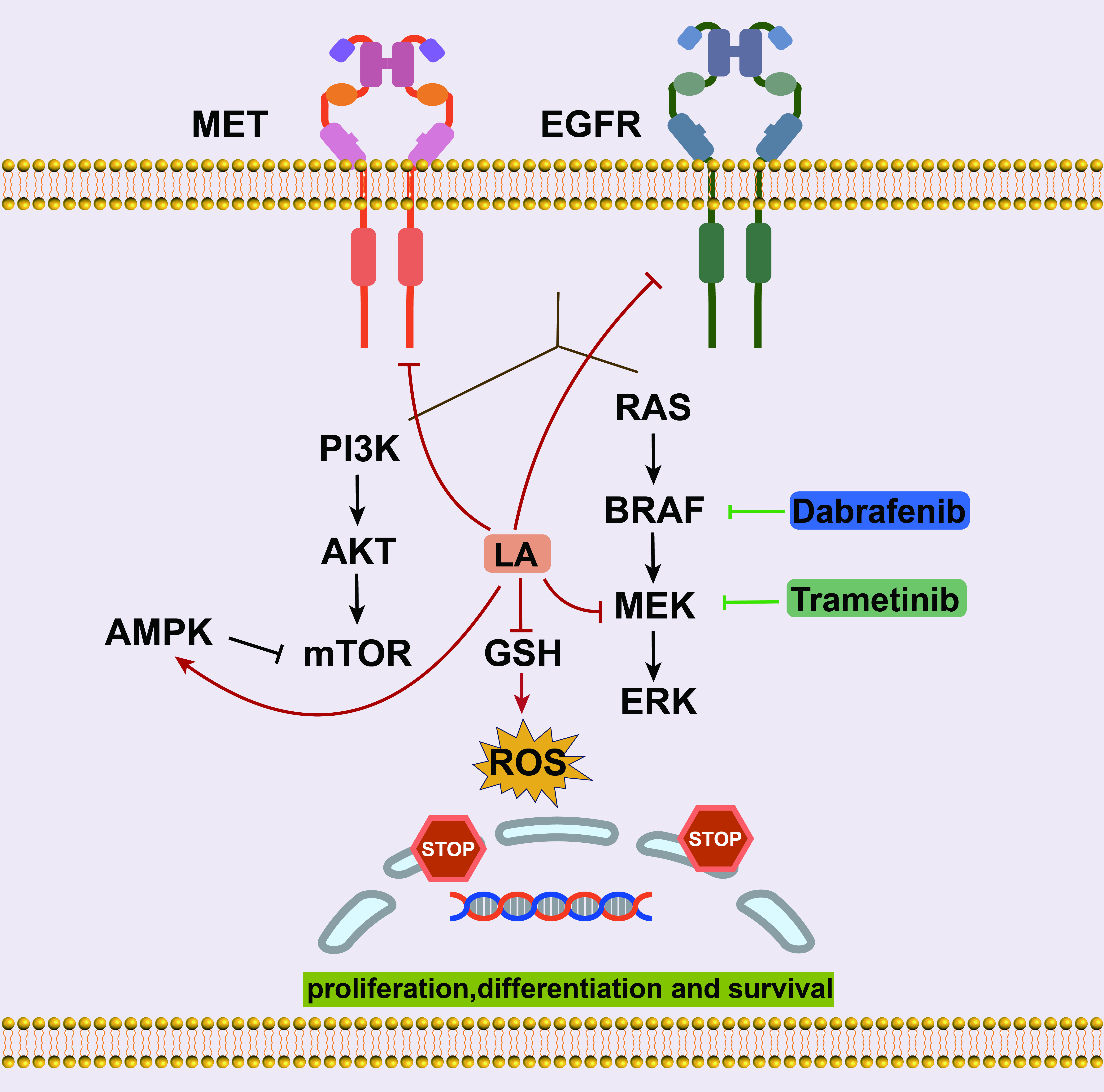

Supplement: Supplementary 1 — Experimental procedures Figs. S1 to S13 Tables S1 to S5 [file research.1180.f1.zip › Figure S3-0.jpg]

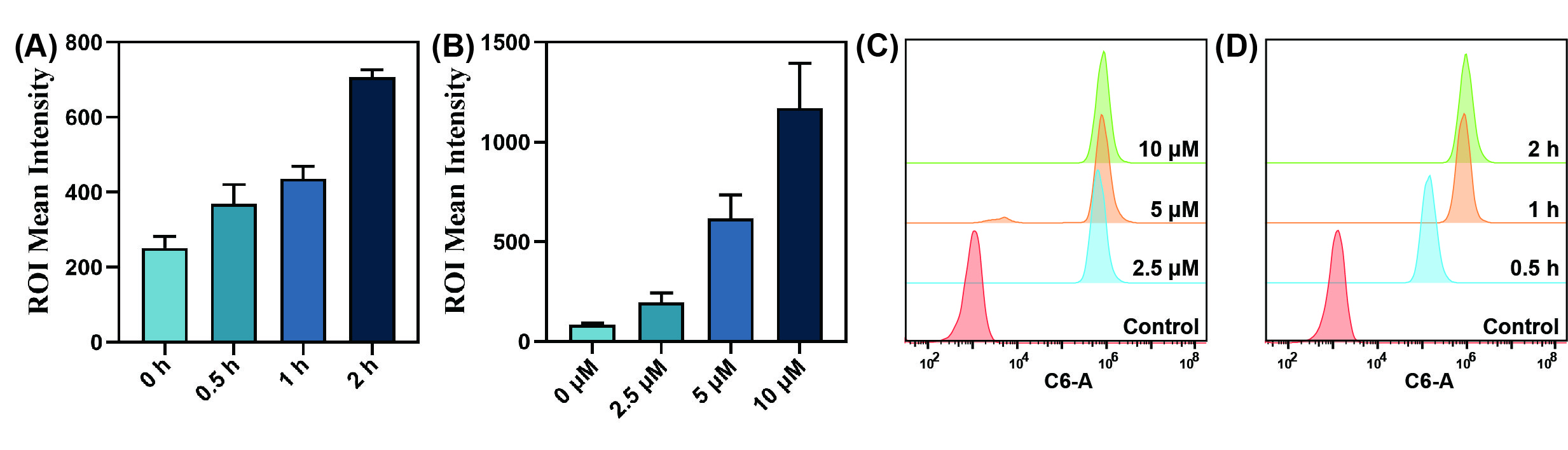

Supplement: Supplementary 1 — Experimental procedures Figs. S1 to S13 Tables S1 to S5 [file research.1180.f1.zip › Figure S3.jpg]

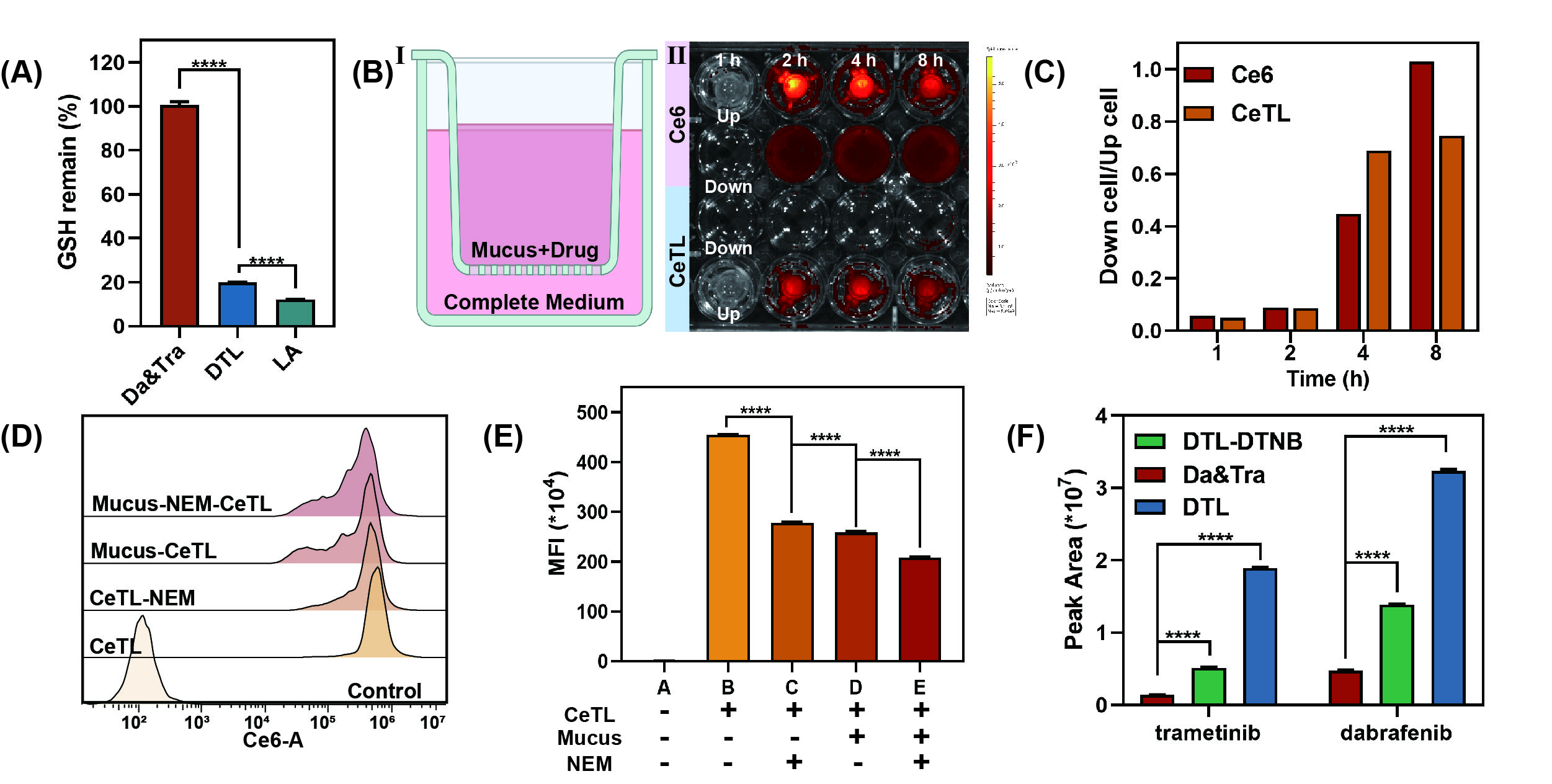

Supplement: Supplementary 1 — Experimental procedures Figs. S1 to S13 Tables S1 to S5 [file research.1180.f1.zip › Figure S4.jpg]

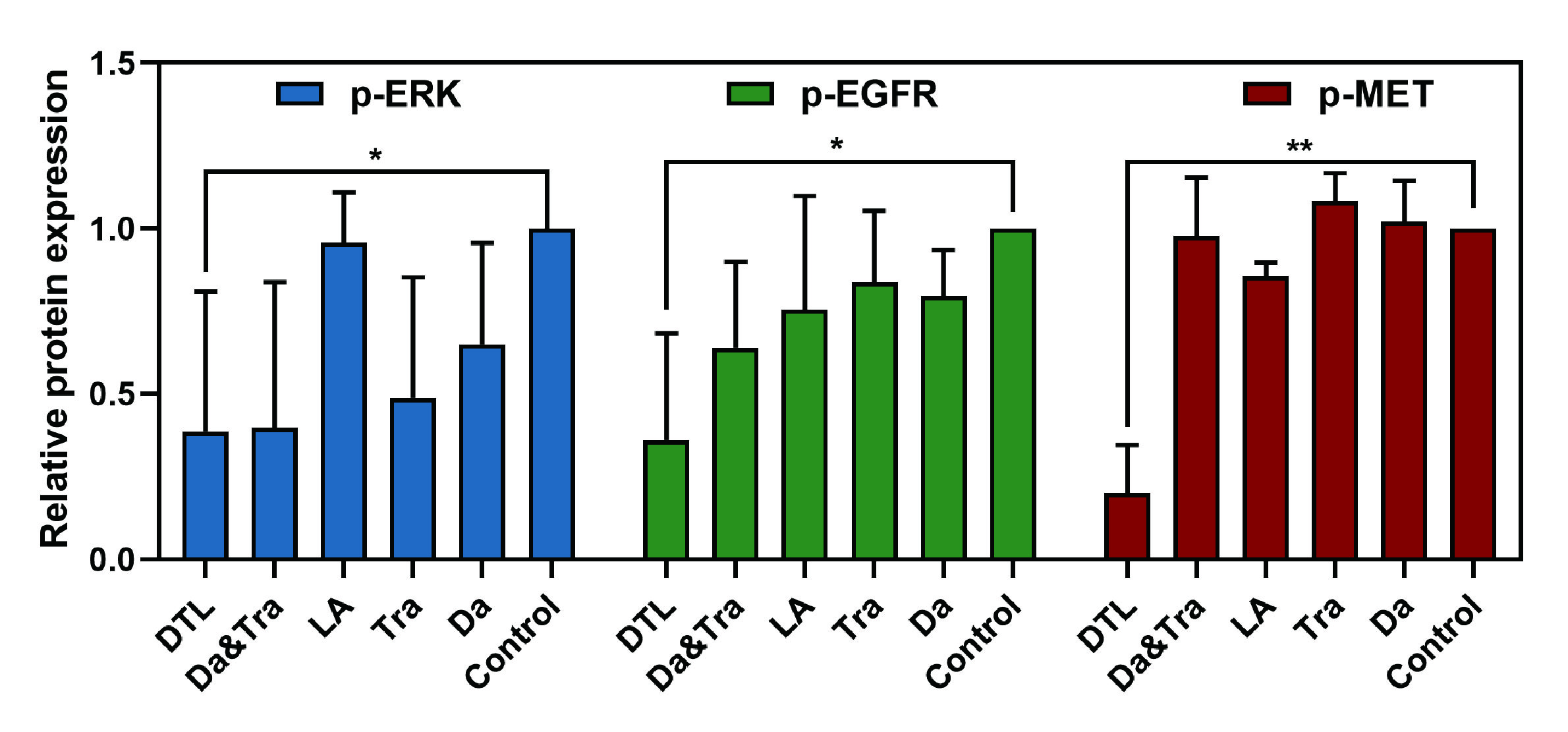

Supplement: Supplementary 1 — Experimental procedures Figs. S1 to S13 Tables S1 to S5 [file research.1180.f1.zip › Figure S5.jpg]

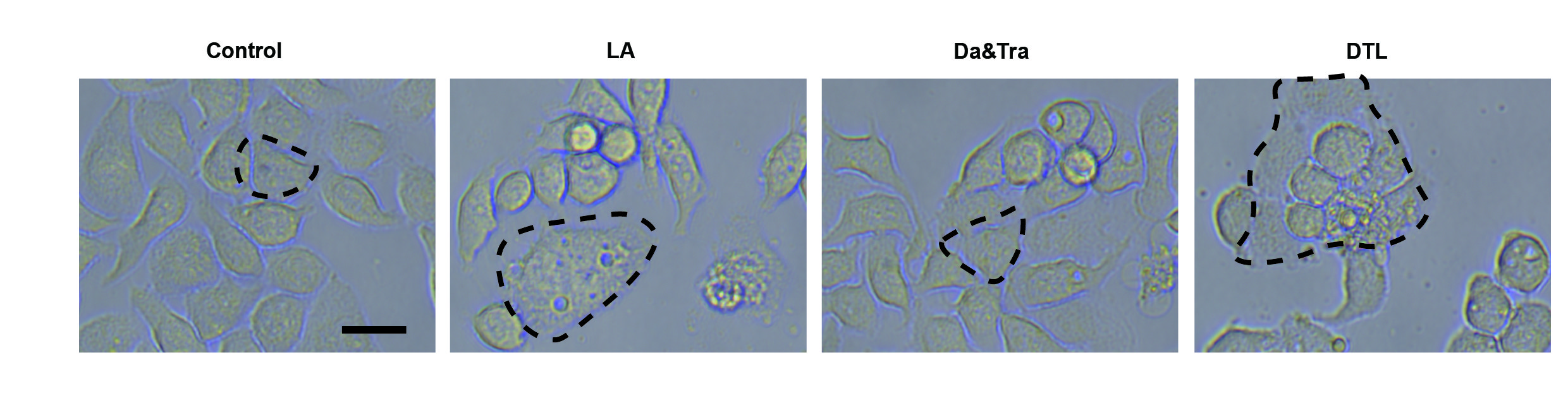

Supplement: Supplementary 1 — Experimental procedures Figs. S1 to S13 Tables S1 to S5 [file research.1180.f1.zip › Figure S6_ 1.jpg]

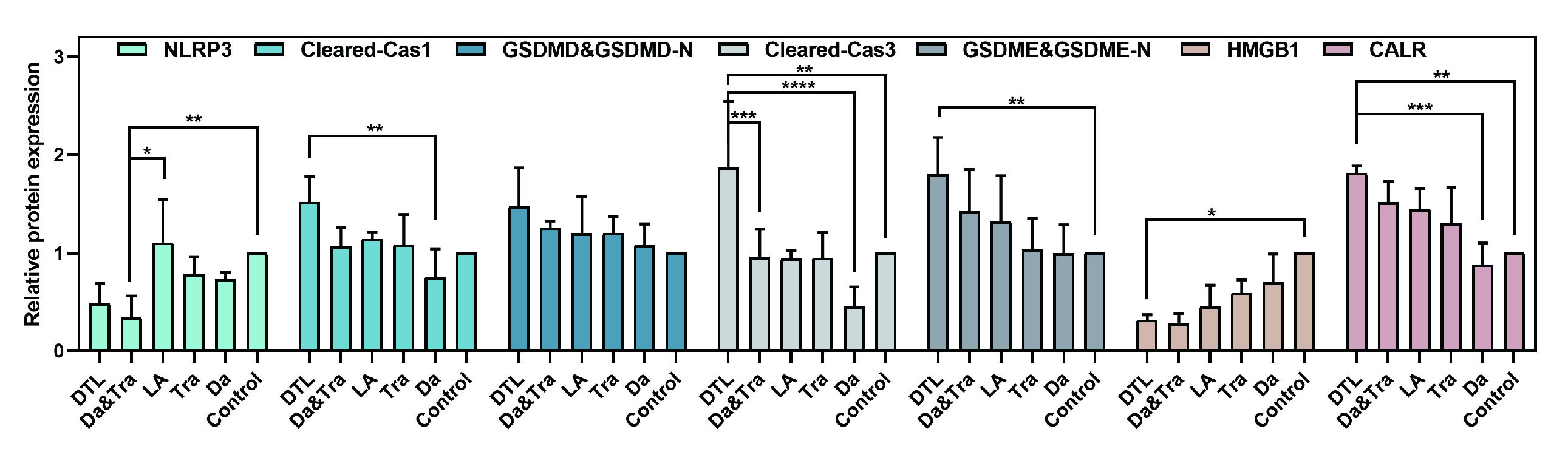

Supplement: Supplementary 1 — Experimental procedures Figs. S1 to S13 Tables S1 to S5 [file research.1180.f1.zip › Figure S7.jpg]

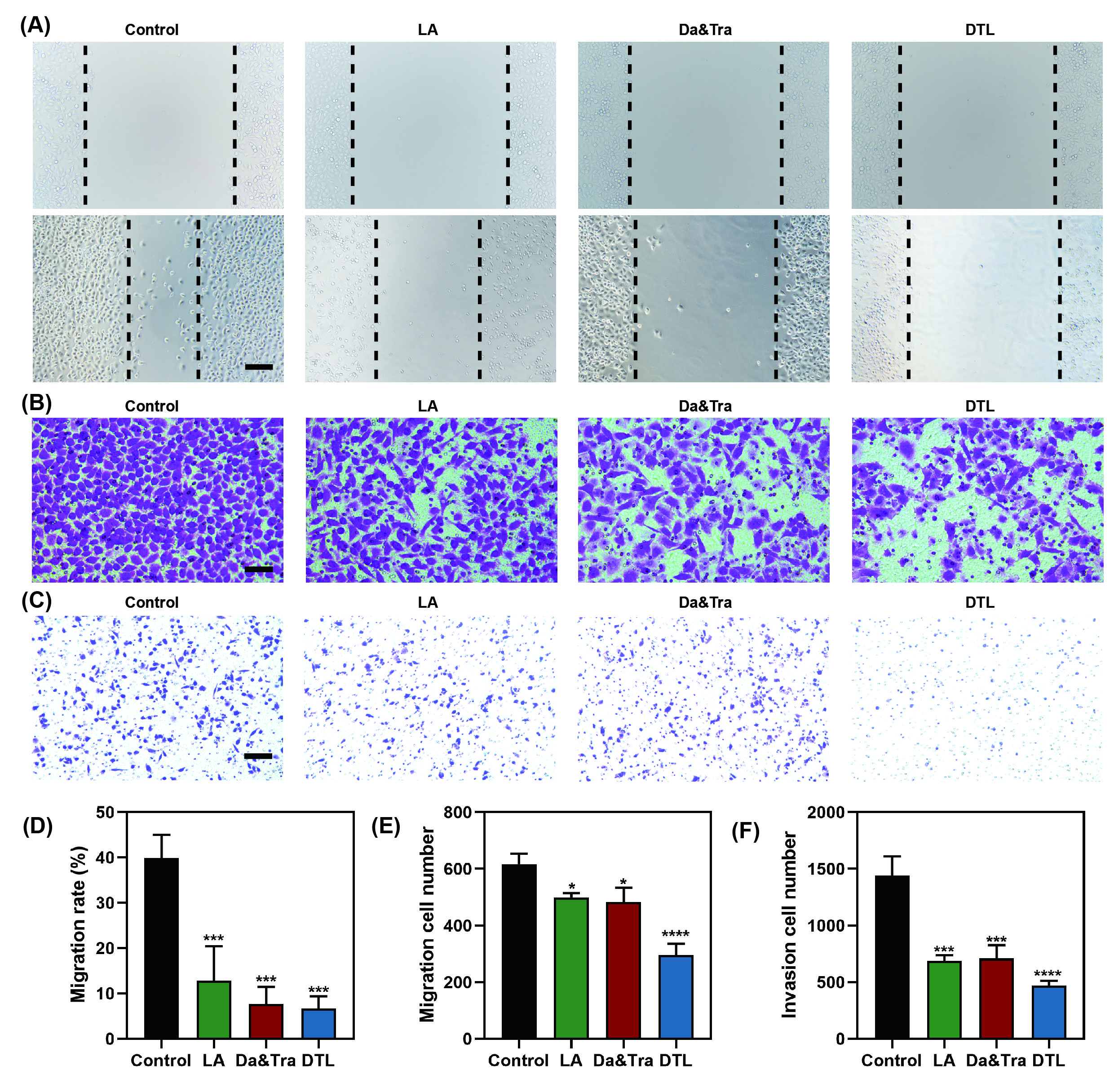

Supplement: Supplementary 1 — Experimental procedures Figs. S1 to S13 Tables S1 to S5 [file research.1180.f1.zip › Figure S8_ 1.jpg]

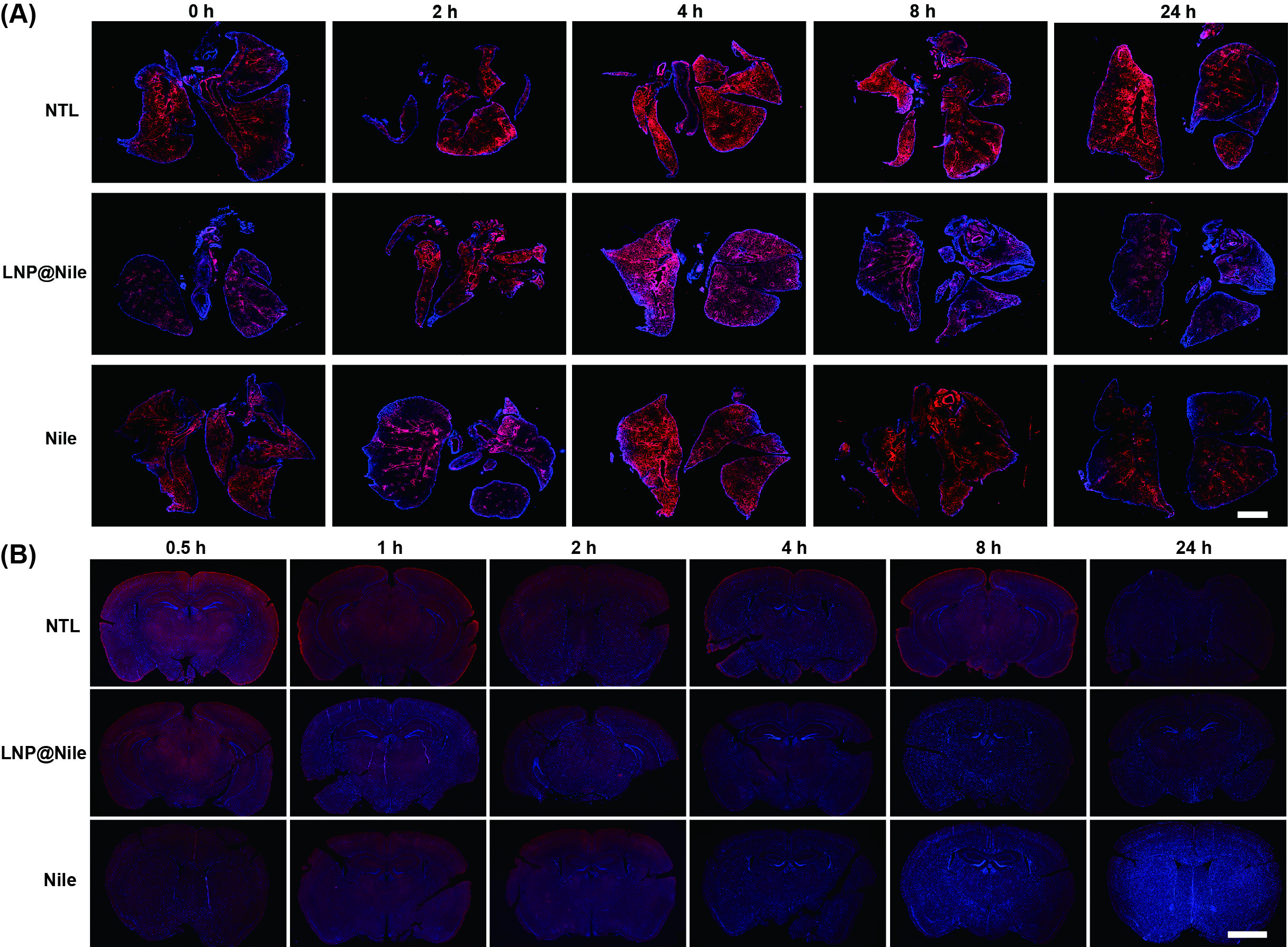

Supplement: Supplementary 1 — Experimental procedures Figs. S1 to S13 Tables S1 to S5 [file research.1180.f1.zip › Figure S9.jpg]

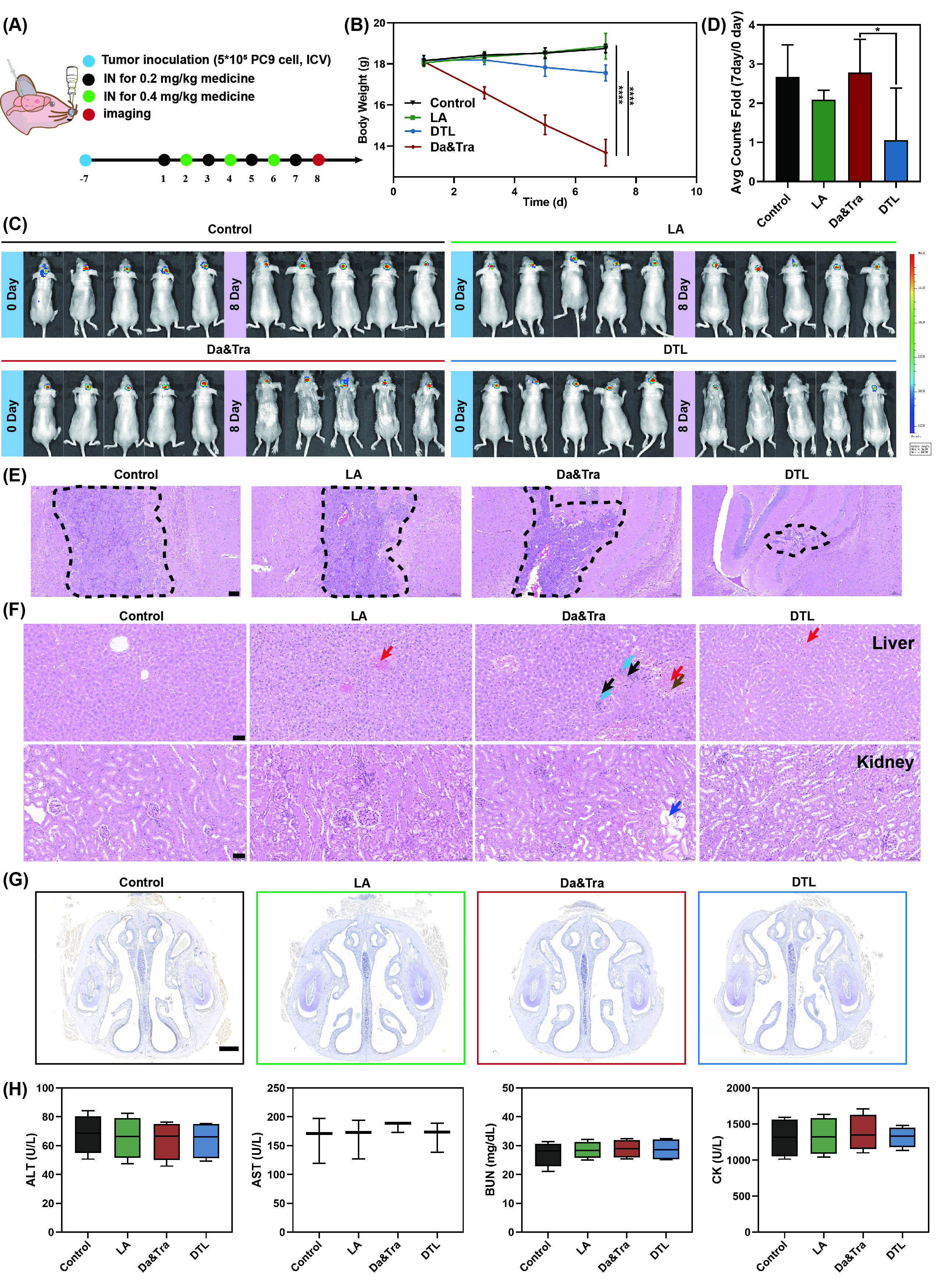

Supplement: Supplementary 1 — Experimental procedures Figs. S1 to S13 Tables S1 to S5 [file research.1180.f1.zip › Figure_9.jpg]

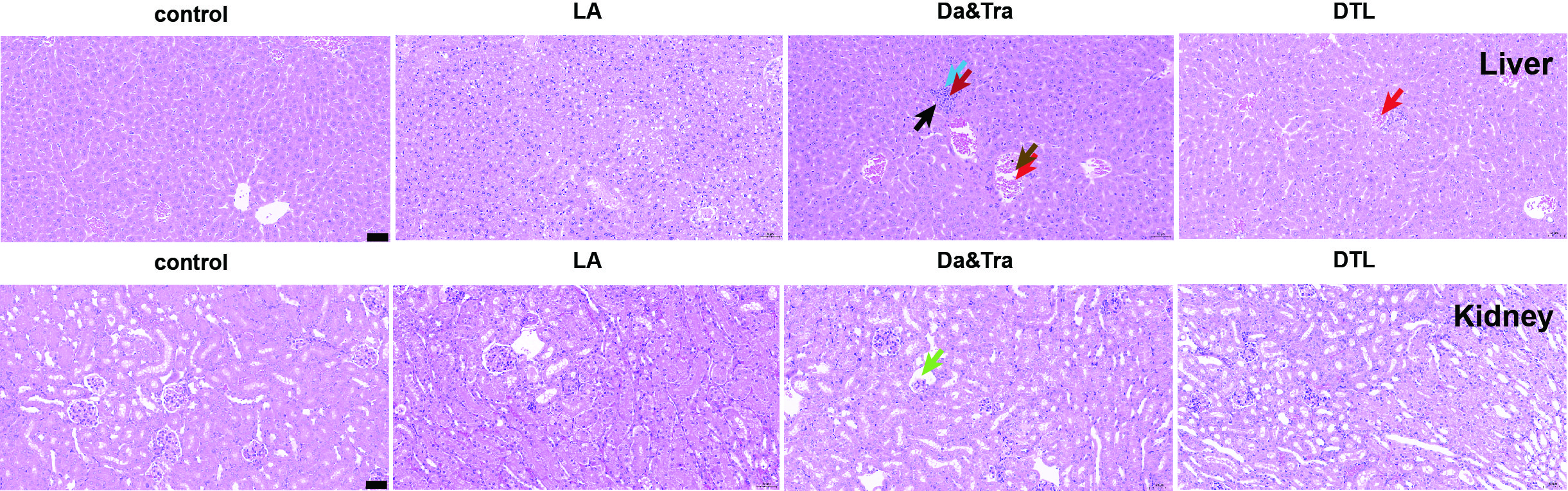

Supplement: Supplementary 1 — Experimental procedures Figs. S1 to S13 Tables S1 to S5 [file research.1180.f1.zip › Figure_S12.jpg]
